# Synthesis, Structural Studies, and Biological Evaluation of Copper(I) and Copper(II) Complexes Supported by Bis(pyrazol-1-yl)acetate Ligand Functionalized with Amantadine for the Treatment of Glioblastoma

**DOI:** 10.3390/ijms27031531

**Published:** 2026-02-04

**Authors:** Sofia Migani, Giuseppina Bozzuto, Annarica Calcabrini, Marisa Colone, Maria Luisa Dupuis, Miriam Caviglia, Cristina Aguzzi, Maria Beatrice Morelli, Fabio Del Bello, Wilma Quaglia, Maura Pellei, Carlo Santini, Chiara Battocchio, Giovanna Iucci, Iole Venditti, Carlo Meneghini, Simone Amatori, Annarita Stringaro

**Affiliations:** 1 School of Science and Technology, Chemistry Division, University of Camerino, via Madonna delle Carceri (ChIP), 62032 Camerino, Italy; sofia.migani@unicam.it (S.M.); miriam.caviglia@unicam.it (M.C.); maura.pellei@unicam.it (M.P.); 2 National Center for Drug Research and Evaluation, Italian National Institute of Health, Viale Regina Elena 299, 00161 Roma, Italy; giuseppina.bozzuto@iss.it (G.B.); annarica.calcabrini@iss.it (A.C.); marisa.colone@iss.it (M.C.); marialuisa.dupuis@iss.it (M.L.D.); 3 School of Pharmacy, Immunopathology and Molecular Medicine Unit, University of Camerino, via Madonna delle Carceri 9, 62032 Camerino, Italy; cristina.aguzzi@unicam.it (C.A.); mariabeatrice.morelli@unicam.it (M.B.M.); 4 School of Pharmacy, Medicinal Chemistry Unit, University of Camerino, via Madonna delle Carceri (ChIP), 62032 Camerino, Italy; fabio.delbello@unicam.it (F.D.B.); wilma.quaglia@unicam.it (W.Q.); 5 Department of Science, Roma Tre University, Via della Vasca Navale 79, 00146 Roma, Italy; chiara.battocchio@uniroma3.it (C.B.); giovanna.iucci@uniroma3.it (G.I.); iole.venditti@uniroma3.it (I.V.); carlo.meneghini@uniroma3.it (C.M.); simone.amatori@uniroma3.it (S.A.)

**Keywords:** copper, amantadine-conjugated ligands, phosphanes, SR-XPS, XAS, spectroscopy, glioblastoma, cytotoxicity, microscopy analyses

## Abstract

This paper reports the synthesis, structural characterization, and biological evaluation of a novel series of Cu^I^ and Cu^II^ complexes supported by an amantadine-functionalized bis(pyrazol-1-yl)acetate ligand (L^Ad^) as potential anticancer agents for the treatment of glioblastoma (GBM). Comprehensive spectroscopic and structural investigations, including SR-XPS, XANES/EXAFS, and DFT modeling, confirmed the successful coordination of L^Ad^ to copper centers in both oxidation states, affording well-defined molecular architectures with distinct coordination geometries. Among the synthesized compounds, the Cu^I^ complexes bearing triphenylphosphine co-ligands (compounds **4** and **5**) exhibited the strongest cytotoxicity against U87 MG and LN18 GBM cell lines, showing IC_50_ values lower than those of cisplatin. These complexes induced a pronounced redox imbalance through reactive oxygen species (ROS) overproduction and glutathione (GSH) depletion, leading to G2/M cell cycle arrest and cell death. Flow cytometry and Western blot analyses demonstrated that cell death occurs via caspase-dependent apoptosis in LN18 cells, as evidenced by PARP cleavage, downregulation of Bcl-xL, release of cytochrome c, and mitochondrial translocation of Bax. Altogether, these findings highlight the potential of lipophilic amantadine-functionalized Cu^I^ complexes as promising anticancer candidates targeting glioma cells through mitochondrial dysfunction and redox-mediated pathways.

## 1. Introduction

Metal-based chemotherapy is a fruitful area of research and application in oncology. In this context, one of the most prominent examples is provided by platinum-based drugs, including cisplatin, carboplatin, and oxaliplatin, which have been widely used to treat various types of cancer [1]. Despite their efficacy, the therapeutic potential of these agents is significantly limited by several factors, such as non-selective cytotoxicity leading to a range of side effects, and chemoresistance. The latter can arise through several mechanisms, including increased DNA repair, altered drug activation, drug efflux, and cellular signaling modifications [2]. A possible strategy consists in the development of platinum complexes bearing one or more bioactive ligands to overcome the above limitations, improve solubility and bioavailability, and/or increase drug potency by combining different mechanisms of action [3,4].

Another promising avenue in the search for alternatives to traditional platinum-based drugs involves the use of different essential ions, including copper [5]. This element plays a crucial role in biological processes and can be exploited to develop complexes targeting specific pathways involved in cancer cell proliferation and survival.

Copper-based complexes show significant antitumor and antimetastatic effects against various types of solid tumors through several mechanisms, such as generation of reactive oxygen species (ROS), DNA damage, glutathione (GSH) depletion, and proteasome inhibition, which differ from those of platinum-based drugs [6,7,8,9,10,11]. Moreover, since endogenous metal ions typically exhibit lower toxicity towards normal cells compared to non-endogenous ones, copper complexes could serve as promising alternatives to platinum-based compounds, potentially overcoming their limitations [12].

Over the years, we have described several copper-based complexes endowed with varying levels of antitumor activity, depending on the metal oxidation state (Cu^I^ or Cu^II^) and the peculiar features of the ligands (lipophilicity, stability, coordination properties, etc.) [6,13,14,15,16,17]. One of the most promising strategies we are pursuing concerns the development of innovative Cu^I^ and Cu^II^ complexes exhibiting significant cytotoxic activities against various human tumor cell lines. These complexes feature bifunctional ligands bearing two pyrazolyl coordinating groups conjugated to biologically active molecules, enabling binding to specific targets [18,19,20,21,22].

In this regard, we have recently reported a series of copper complexes in which the bis(3,5-dimethyl-pyrazol-1-yl) acetic acid conjugated to the drug amantadine has been used as a ligand. Amantadine is a known antiviral drug which disrupts the transmembrane domain of the viral M2 protein, preventing the virus’ genetic material from entering the host cell [23]. It is also used as an anti-Parkinson agent by acting as a weak non-competitive NMDA (N-methyl-D-aspartate) receptor antagonist and promoting dopamine release from neurons [24]. It was selected as a bioactive molecule because it has recently been shown to exert antiproliferative effects against several human tumor cell lines, including glioblastoma (GBM) [25,26,27,28]. Moreover, Pt-based complexes functionalized with amantadine have been reported to display promising cytotoxic activity against a variety of human tumor cell lines [29]. Finally, amantadine contains a primary amine group suitable for conjugation with the carboxylic group of the bifunctionalizable species and features a bioversatile adamantyl scaffold, a structural motif shared by several derivatives identified as anticancer agents [29,30]. Interestingly, two of the reported complexes significantly decrease cell viability, and affect cell proliferation and death of glioblastoma (GBM) cell lines [31]. These results are particularly relevant, considering that GBM is one of the most aggressive forms of malignant primary brain cancer in adults. Currently, radiotherapy, surgery, and available pharmacological treatments, such as temozolomide (TMZ), are not enough to effectively manage this condition. Mortality rates are very high, with a median survival of 12–15 months. A significant obstacle to successful treatments arises from the aggressive nature and high level of intra-tumor heterogeneity associated with this primary brain tumor [32,33].

**Scheme 1:**
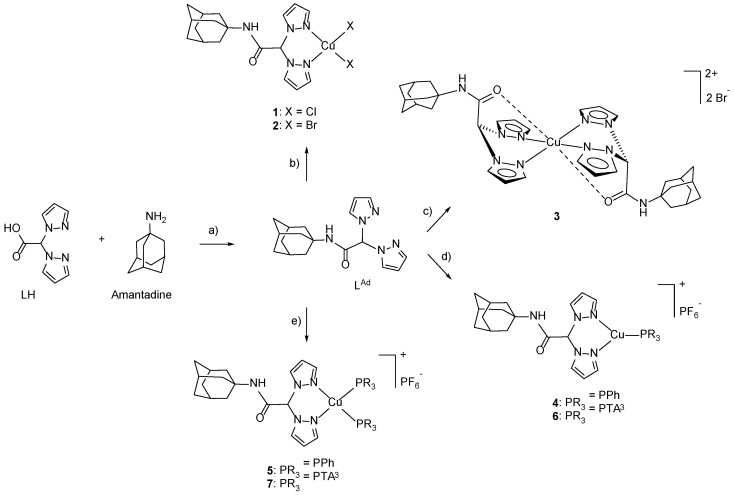
Reaction scheme for the synthesis of ligand L^Ad^ and complexes **1**–**7**. Reagents: (**a**) TBTU, DIPEA, DMF, rt 20 h; (**b**) 1 eq CuCl_2_ for **1**, 1 eq CuBr_2_ for **2**, CH_3_CN; (**c**) 0.5 eq CuBr_2_, CH_3_CN; (**d**) 1 eq PPh_3_ for **4**, 1 eq PTA for **6**, [Cu(CH_3_CN)_4_]PF_6_, CH_3_CN; (**e**) 2 eq PPh_3_ for **5**, 2 eq PTA for **7**, [Cu(CH_3_CN)_4_]PF_6_, CH_3_CN.

Encouraged by these promising results, we prepared and studied a novel series of Cu^I^ and Cu^II^ complexes bearing amantadine, aiming to further elucidate the potential of such compounds as anti-GBM agents. Specifically, amantadine was conjugated to the bifunctional species bis(pyrazol-1-yl)acetic acid (LH), selected as the coordinating agent due to its κ^3^-NNO coordination mode and the presence of a carboxylic group suitable for conjugation with the primary NH_2_ group of amantadine, yielding the ligand L^Ad^. This ligand was subsequently used as a chelating agent to prepare the novel Cu^II^ complexes **1**–**3** and Cu^I^ complexes **4**–**7** (Scheme 1). To stabilize copper in the +1 oxidation state of compounds **4**–**7**, triphenylphosphine (PPh_3_) and 1,3,5-triaza-7-phosphaadamantane (PTA) were used as lipophilic and hydrophilic co-ligands, respectively, imparting different solubility profiles to the corresponding complexes.

## 2. Results and Discussion

### 2.1. Synthesis and Characterization

Amantadine was reacted with LH in the presence of TBTU and DIPEA at room temperature for 20 h to give the ligand L^Ad^ after separation and purification by column chromatography in 86% yield (Scheme 1). Its purity was confirmed by combustion analysis and its elemental composition agreed to within ± 0.4% of the calculated value (elemental analysis (%) calculated for C_18_H_23_N_5_O: C 66.44, H 7.12, N 21.52; found: C 66.73, H 7.00, N 21.31).

The IR spectrum of a solid sample of L^Ad^ showed all the expected bands for the ligand: weak CH stretching absorptions were observed in the range 2890–3146 cm^−1^, while a broad peak corresponding to the N-H amide stretching appeared at 3281 cm^−1^. Moreover, the asymmetric stretching of the C=O group was detected as a very strong peak at 1670 cm^−1^, within the typical range for the amide groups. The ^1^H- and ^13^C-NMR spectra of L^Ad^ in CDCl_3_ solution showed all the expected signals for the ligand, and the two-dimensional (2D) Heteronuclear Single Quantum Coherence (HSQC) experiment, used to determine proton-carbon single bond correlations, enabled the complete assignment of all ^1^H- and ^13^C-NMR signals. A single set of resonances appears for the pyrazole rings in the ^1^H-NMR spectrum, indicating that they are equivalent: a triplet at 6.36 ppm and two doublets at 7.64 and 7.74 ppm, attributable to the 4-C*H*_Pz_, 5-C*H*_Pz_, and 3-C*H*_Pz_, respectively. The ESI-MS study was conducted by dissolving the ligand in CH_3_OH and recording the spectra in positive- and negative-ion mode. The molecular structure of L^Ad^ is confirmed by the presence in the positive-ions spectrum of the molecular peaks at *m*/*z* 326, 348, and 673, due to the [L^Ad^ + H]^+^, [L^Ad^ + Na]^+^, and [2L^Ad^ + Na]^+^ adducts, respectively.

The complexes [Cu(L^Ad^)Cl_2_] (**1**) and [Cu(L^Ad^)Br_2_] (**2**) were synthetized by reacting the ligand L^Ad^ with copper(II) acceptors CuCl_2_·2H_2_O and CuBr_2_, respectively, in a 1:1 stoichiometric ratio in CH_3_CN (Scheme 1). The synthesis of complex **1** was performed under reflux, whereas complex **2** was prepared at room temperature. [Cu(L^2Ad^)_2_]Br_2_ (**3**) was obtained using L^Ad^ and CuBr_2_ in the stoichiometric ratio 2:1 in CH_3_CN at room temperature (Scheme 1). Bromide is less coordinative than chloride, allowing the coordination of copper with two ligands. FT-IR spectra of the complexes display all expected bands. Notably, strong absorptions for the asymmetric C=O stretch are observed in the range 1663–1665 cm^−1^, showing a slight shift from the corresponding band in free L^Ad^ (1670 cm^−1^). In the far-IR spectrum of **1**, a very strong absorption at 281 cm^−1^ is assigned to the Cu–Cl stretching frequency. Similarly, strong absorptions in the range 220–242 cm^−1^ in the far-IR spectra of complexes **2** and **3** are attributed to Cu–Br stretching modes [34]. ESI-MS spectra were recorded for complexes **1**–**3**, with **1** dissolved in CH_3_CN and **2** and **3** in CH_3_OH. In the positive-ion mode spectrum of **1**, peaks at *m*/*z* 423 and 748 correspond to the species [(L^Ad^)CuCl]^+^ and [(L^Ad^)_2_CuCl]^+^, respectively. The ESI-MS(+) spectra of complexes **2** and **3** show a peak at *m*/*z* 469 for the fragment [(L^Ad^)CuBr]^+^. For complex **3**, additional signals at *m*/*z* 358, 712, and 794, assigned to the fragments [(L^Ad^)_2_Cu]^2+^, [(L^Ad^)_2_Cu]^+^, and [(L^Ad^)_2_CuBr]^+^, further confirm the coordination of two ligands to the copper center.

Both triphenylphosphine copper(I) complexes [Cu(L^Ad^)(PPh_3_)]PF_6_ (**4**) and [Cu(L^Ad^)(PPh_3_)_2_]PF_6_ (**5**) were synthetized in CH_3_CN using, as starting materials, the ligand L^Ad^, the metal acceptor [Cu(CH_3_CN)_4_]PF_6_, and the PPh_3_ co-ligands in stoichiometric ratios 1:1:1 and 1:1:2, respectively. Analogously, 1,3,5-triaza-phosphaadamantane copper(I) complexes [Cu(L^Ad^)(PTA)]PF_6_ (**6**) and [Cu(L^Ad^)(PTA)_2_]PF_6_ (**7**) were synthetized in CH_3_CN using PTA as the co-ligands in the stoichiometric ratio metal:phosphane 1:1 and 1:2, respectively. Elemental analyses and spectroscopic studies such as FT-IR, ^1^H-, ^13^C-, ^31^P-NMR, and ESI-MS confirm the different stoichiometries of the synthesized complexes **4**–**7**. All the expected absorption bands were observed in the FT-IR spectra. In particular, in the range 2853–3149 cm^−1^, the complexes exhibit weak bands typical of C-H stretching, while weak broad peaks attributable to the N-H stretching are visible at 3318–3393 cm^−1^. The strong absorptions at 1670–1701 cm^−1^ are assigned to the asymmetric stretching of the carbonyl groups, while very intense absorptions in the range 833–837 cm^−1^ correspond to the stretching of the PF_6_^−^ counterion. The ^1^H-NMR spectra, recorded in CD_3_CN for complexes **4**–**7**, confirm the stoichiometric ratio between the heteroscorpionate ligand and the phosphane co-ligands. They showed a single set of resonances for the pyrazole rings, indicating that the pyrazole protons are equivalent. The signals of adamantane are clearly visible in the range 1.65–2.22 ppm, while the signal of the C*H*CO protons is visible at δ 6.94–7.04 ppm, with a slight shift relative to the signal of the free ligand due to the coordination to the copper acceptor. The aromatic hydrogens of the triphenylphosphine co-ligands of **4** and **5** are detectable in the range 7.32–7.54 ppm, while in the spectra of compounds **6** and **7**, the NC*H*_2_P protons of the PTA co-ligands are visible as singlets at δ 4.08–4.10 ppm, and the corresponding NC*H*_2_N protons show characteristic AB quartets in the range 4.49–4.62 ppm. The ^31^P-NMR spectra of complexes **4** and **5**, recorded in CD_3_CN, give broad singlet peaks at −0.81 and −0.47 ppm, respectively, downfield-shifted relative to the value of the free triphenylphosphine in the same solvent (δ = −4.85 ppm). Analogously, in the spectra of the PTA complexes **6** and **7**, recorded in CD_3_CN, singlets are visible at −94.53 and −89.91 ppm, respectively, downfield-shifted relative to the value of the free PTA in CD_3_CN (δ = −102.07 ppm). Moreover, the spectra of complexes **4**–**7** show the distinctive septets due to the presence of the PF_6_^−^ counterion at about −144.6 ppm. The ESI-MS study was performed by dissolving complexes **4**–**7** in acetonitrile and recording the spectra in both positive- and negative-ion modes. ESI-MS(+) spectra of complexes **4** and **5** showed peaks at *m*/*z* 357, 587 and 650, which can be attributed to the species [Cu(PPh_3_)]^+^, [Cu(PPh_3_)_2_]^+^, and [Cu(L^Ad^)(PPh_3_)]^+^, respectively. Analogously, the formation and stability of complexes **6** and **7** are confirmed by the presence in their ESI-MS(+) spectra of the species [Cu(L^Ad^)(PTA)]^+^ as a major peak at *m*/*z* 545. In the negative-ion spectra of complexes **4**–**7**, [PF_6_]^−^ was observed as the major peak at *m*/*z* 145.

The spectroscopic characterization of complexes **1**–**7** (FT-IR, ^1^H-, ^13^C{^1^H}-, and ^31^P{^1^H}-NMR spectra) are available in the Supporting Information Figures S1–S22.

### 2.2. X-Ray Photoelectron Spectroscopy

Both series of Cu^I^ and Cu^II^ coordination compounds were investigated by SR-XPS to assess the ligand’s molecular stability upon copper ion coordination and to investigate the complex electronic structures, with particular attention devoted to the copper ion oxidation state stability. All SR-XPS spectra collected on the seven copper samples are reported in the Supporting Information Figures S23–S26; complete SR-XPS data analysis results (Binding Energy—BE (eV), Full Width Half Maximum—FWHM (eV)), atomic percentages, and proposed assignments for all measured signal components are summarized in Table S1.

**Figure 1:**
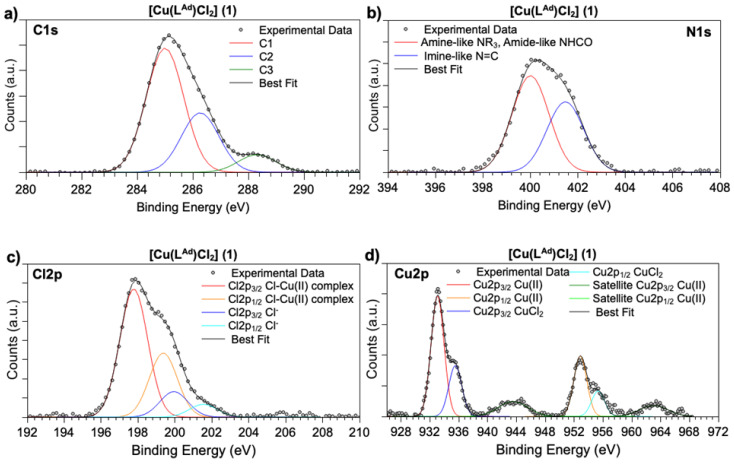
(**a**) C1s, (**b**) N1s, (**c**) Cl2p, and (**d**) Cu2p spectra collected on Cu^II^ coordination compound **1**, representative also for **2** and **3**. C1s, N1s, Br3d, and Cu2p spectra of **2** and **3** are reported in the Supporting Information Figures S23 and S24.

For the Cu^II^ coordination compounds **1**–**3**, SR-XPS measurements were performed at C1s, N1s, O1s, Br3d, Cl2p, and Cu2p core levels, consistent with their proposed molecular structures. Given the high similarity of the spectral shapes across the three samples, the data collected on complex **1** are presented as a representative example in Figure 1.

C1s spectra (Figure 1a) appear asymmetric; peak-fitting reveals at least three components associated with aliphatic C-C bonds (285.0 eV BE), C-N bonds (286.3 eV BE), and the N-C=O group (about 288.3 eV BE), confirming the ligand’s stability. N1s spectra (Figure 1b) show three components at about 400 and 401.2 eV BE, attributed to amine- or amide-like N atoms (NR_3_, NHC=O), whose position cannot be distinguished within the experimental resolution, and imine-like (N=C) nitrogen atoms. Cu2p spectra (Figure 1d) display two components at about 932.5 and 935.5 eV BE, corresponding to Cu^II^ in the coordination compounds [35,36] and to CuCl_2_ or CuBr_2_ [37], respectively. The Cl2p spectrum of **1** (Figure 1c) is composite, showing two spin orbit pairs: one at lower binding energy indicative of Cl-Cu in the coordination complex, and another at higher binding energy assigned to CuCl_2_ [36,38]. The Br3d spectrum of **2** (left side, Figure S24, middle) also consists of two spin-orbit pairs with Br3d_5/2_ components around 68.4 eV and 70 eV BE, indicative of Br-Cu in the coordination complex and CuBr_2_, respectively [36]. In contrast, the Br3d spectrum of **3** (left side, Figure S24, bottom) shows a single spin-orbit pair associated with bromine atoms bonded to Cu^II^ ions in the coordination compound (Br3d_5/2_ BE = 68.3 eV).

**Figure 2:**
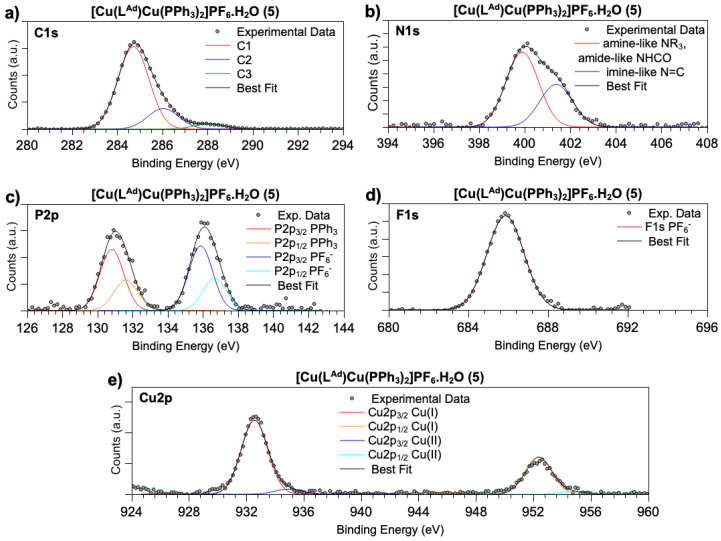
(**a**) C1s, (**b**) N1s, (**c**) P2p, (**d**) F1s, and (**e**) Cu2p spectra collected on the Cu^I^ coordination compound **5**, representative also for **4**, **6**, and **7**. C1s, N1s, P2p, F1s, and Cu2p spectra of **4, 6,** and **7** are reported in the Supporting Information Figures S25 and S26.

Similarly to Cu^II^ complexes, SR-XPS spectra of the four Cu^I^ coordination compounds **4**–**7** were collected at C1s, N1s, F1s, O1s, P2p, and Cu2p core levels and have analogous shapes in each complex; spectra collected for complex **5** are presented as a representative example in Figure 2.

The C1s spectra of **4** and **5** (Figure 2a and Figure S25) show three components at about 284.7, 286.2, and 288.0 eV BE, associated with aromatic C=C (arising by the PPh_3_ ligands contribution), C-N and N-C=O, respectively, consistent with the ligand structure. The C1s spectra of **6** and **7** (Figure S25, left side, last two rows) exhibit a similar pattern, with three peaks at 285.0, 286.3, and 288.0 eV BE due to aliphatic C-C, C-N and N-C=O, respectively. Similarly to the Cu^II^ complexes, all N1s spectra display two components at about 400 and 401.5 eV, attributed to amine/amide-like and imine-like nitrogen (Figure 2b). P2p spectra (Figure 2c) show two components: the low BE spin-orbit pair has the P2p_3/2_ feature at about 131 eV, as expected for phosphane ligands (PPh_3_ in **4**, **5** and PTA in **6**, **7**), while the high-BE signal (P2p_3/2_ BE = 136–136.5 eV) is characteristic of phosphorus in the PF_6_^−^ counterion. F1s spectra (Figure 2d) show a single component at about 686 eV BE, confirming the presence of PF_6_^−^. As for Cu2p spectra (Figure 2e), samples **4**–**6** exhibit two pairs of spin-orbit components arising by a main signal at about 933.0 and a smaller contribution at 935.0 eV, suggesting the presence of a large amount of Cu^I^ [37] (about 93%, see Table S1) and a small fraction of Cu^II^ (about 7%), probably due to spontaneous oxidation of Cu^I^ ions on the solid sample surface during the deposition procedure or under the SR X-ray beam. However, it is noteworthy that XPS is a surface-sensitive technique, having sampling depth of a few nm (3–5 nm) [39] and that the incertitude on semi-quantitative analysis carried out by XPS is about 5% [40], then the detection of a very small amount of Cu^II^ is, in our opinion, not representative of the copper ion oxidation state in the compound bulk, and complexes **4**–**6** are to be considered as Cu^I^ coordination compounds, as confirmed by XANES. In complex **7**, only the spectral component related to Cu^I^ ions is observed (Figure S26).

**Figure 3:**
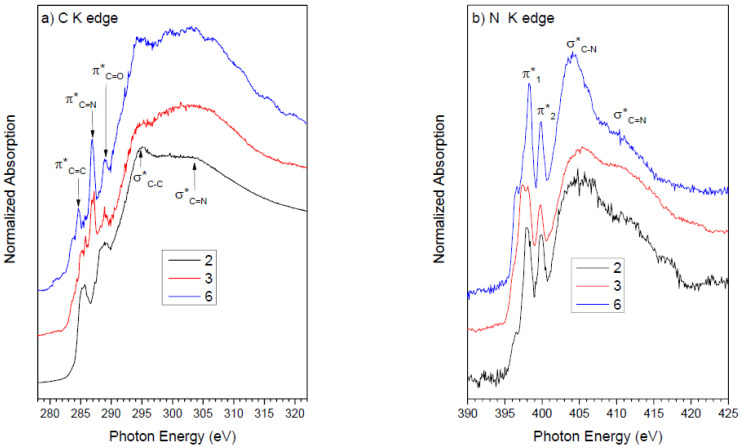
C K edge (**a**) and N K edge (**b**) NEXAFS spectra of complexes **2**, **3**, and **6**.

NEXAFS spectra at the C and N K edges were also recorded for all the investigated samples. Due to their high similarity, only the spectra of complexes **2**, **3**, and **6** are presented as examples in Figure 3a,b.

The two prominent peaks below the edge in the C K edge spectra can be assigned to C1s⟶π* transitions arising from C=C (π*_C=C_ at about 284.6–285.0 eV) and C=N (π*_C=N_ at about 286.9 eV) functions of the pyrazole rings in the L^Ad^ ligand, as previously reported for similar systems [31]; the π*_C=N_ resonance is very clear in the spectra of complexes **3** and **6**, but appears as a shoulder in the spectrum of complex **2**. The third C1s⟶π* transition at 289.0 eV (π*_C=O_) arises from the C=O bond of the amide function in L^Ad^ [41]. The two broad σ* resonances above the edge are mainly due to C-C (σ*_C-C_ at about 294 eV) and C=N (σ*_C=N_ at about 304 eV) bonds [42].

In the N K edge spectra of the investigated samples, the two peaks detected below the edge, labelled π*_1_ and π*_2_ in Figure 3b and located at about 398.0 and 399.8 eV, respectively, are assigned to N1s⟶π* transitions arising from nitrogen atoms of the pyrazole rings. Above the edge, two broad σ* resonances arising from C-N (σ*_C-N_ located at 404–405 eV) and C=N (σ*_C=N_ at about 410 eV) bonds are detected.

### 2.3. X-Ray Absorption Spectroscopy

X-ray Absorption Fine Structure spectroscopy (XAFS) was applied at the Cu K edge to determine the average valence state, coordination chemistry, and local atomic structure around the Cu sites. Analysis of the XAFS data was performed in both the near edge (XANES) and extended (EXAFS) regions of the spectra, providing complementary information on the valence state and coordination geometry of the absorber, as well as describing the average local atomic coordination around the absorber [43]. Quantitative analysis of the EXAFS data was performed using realistic atomic clusters representing the expected structure of the samples around the absorber atom. These models enabled the computation of the amplitude and phase functions required to calculate the theoretical EXAFS signals. Geometry optimization methods paired with Density Functional Theory (DFT) were used to prepare the atomistic structures following an established approach from similar studies [15], as briefly described in the following paragraph.

### 2.4. Structural Models for XAS Data Analysis: DFT Calculations

**Figure 4:**
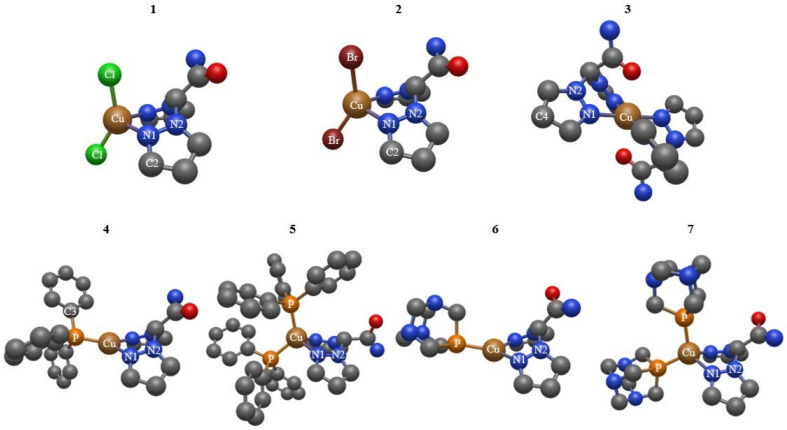
DTF-optimized models for coordination compounds **1**–**7**: Cu^II^ (**1**–**3**) and Cu^I^ (**4**–**7**). The neighboring atoms involved in the most relevant scattering paths for EXAFS analysis are highlighted in the image. Numbering of the neighbors follows the increase in radial distance from the absorber. Hydrogen atoms removed for clarity.

Realistic atomic models of complexes **1**–**7** around the Cu sites were obtained by a Quasi Newton optimization method using DFT. A reasonable guess geometry was prepared for each complex using the 3D open-source software Avogadro [44]. DFT calculations were performed using the open-source software ORCA 5.0.1 (using BP86 energy functional) [45]. Karlsruhe orbital basis sets were used, specifically def2-SVP (Valence Double Zeta) for lighter atoms (H, C, N, O, P) and def2-TZVP (Valence Triple Zeta) for Cu atoms. The Cu compound structural models were relaxed to an absolute minimum of energy, and the obtained structures are shown in Figure 4. The atomic clusters were then used to calculate the theoretical amplitude and scattering functions required to build the theoretical EXAFS functions, using the FEFF8.4 program [46]. The main scattering paths (single scattering, SS, or multiple scattering, MS) were identified and used for analysis. The computational procedures follow an established approach previously applied to similar systems [47].

**Figure 5:**
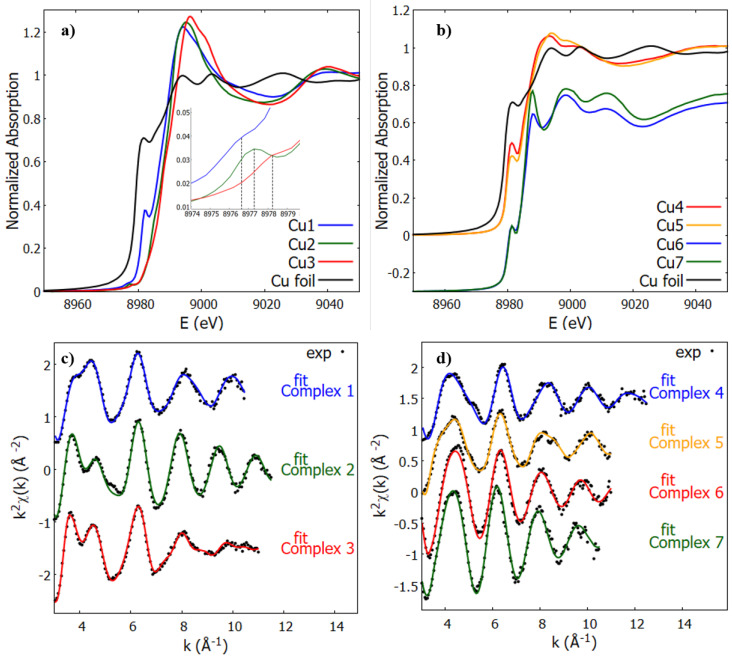
Cu K-edge XANES region for Cu^II^ (**a**) and Cu^I^ (**b**) complexes. Comparison between experimental data and fit results in K space for Cu^II^ (**c**) and Cu^I^ (**d**) complexes. Inset in panel (**a**) highlights the pre-edge region of the spectra.

The Cu^II^ (**1**–**3**) and Cu^I^ (**4**–**7**) complexes were probed using X-ray Absorption Spectroscopy (XAS) at the Cu K edge. Measurements were carried out at the European Synchrotron Radiation Facility (ESRF) on beamline BM08 [48] to determine their local atomic structure around the absorber. The normalized XANES spectra for Cu^II^ complexes **1**–**3** (shown in comparison with the signal of metallic Cu as a reference) are presented in Figure 5a. The edge positions for these three samples at 8981, 8984, and 8987 eV, respectively, as well as the shapes of the XANES region, are compatible with Cu^II^ oxidation state [49]. Analysis of the pre-edge region reveals a 1s→3d transition with different intensities (Figure 5a, inset), indicating a different coordination for the three complexes. Complex **1** exhibits intermediate intensity between **2** (highest) and **3** (lowest). This electric dipole transition is forbidden and usually found with low intensity due to the contribution of only an electric quadrupole. The difference between **1** and **2** (both with coordination number 4) points to two different configurations, with complex **1** in a square planar environment, while complex **2** adopts a tetrahedral geometry. The latter shows an increased intensity due to an electric dipole contribution resulting from p-d orbital mixing [50,51]. Complex **3** (coordination number 6) shows a weak pre-peak at higher energy, that is consistent with the presence of an octahedral configuration where p-d mixing is absent and only electric quadrupole contributions to this transition are allowed [51]. Complex **1** also features a characteristic edge peak at 8984 eV that is reduced to a shoulder in complex **2** and is completely missing in complex **3**. This peak is related to a dipole allowed 1s→4p transition, supporting the square planar assignment for **1** and tetrahedral geometry for **2**. The lack of this transition in **3** is consistent with an octahedral coordination, as no edge peak is observed in the literature for similar systems.

The XANES spectra for Cu^I^ complexes **4**–**7** are shown alongside metallic Cu for comparison (and vertically shifted for clarity) in Figure 5b. All complexes show no peaks in the pre-edge region and an absorption edge position characteristic of Cu^I^ (8989.8, 8979.3, 8979.7, and 8980.2 eV, respectively). The intense peak centered at 8982 eV is due to the dipole allowed photoelectron transition 1s→4p. The peak position and shape of the XANES region are consistent with the Cu^I^ oxidation state, especially in the presence of the characteristic edge peak, reported in literature for most Cu^I^ compounds [49]. This series of complexes is characterized by the presence of two different phosphane ligands, triphenylphosphine for complexes **4** and **5** and PTA for complexes **6** and **7**, as well as different coordination numbers (3 for **4** and **6** and 4 for **5** and **7**). Nevertheless, the edge features are very similar across the samples. This is due to the electronic structure of Cu^I^ which features a complete 3d shell (3d^10^). Hence, on one hand, no possible transitions to this level can be observed, and no stabilization of the complex structure can occur, while the only coordination geometries achievable by these systems are trigonal planar and tetrahedral. A key difference between the two groups of complexes appears after the edge, with complexes **4** and **5** showing a smooth XANES region, while **6** and **7** show an additional peak at 8988 eV. This difference between the two pairs of complexes may reflect a distortion in the local coordination environment. Rather than ideal trigonal planar or tetrahedral geometries, complexes **6** and **7** likely adopt slightly distorted structures: a T-shaped configuration for **6** and a compressed tetrahedron for **7** [49]. It is also possible that this contribution to the XANES region is related to the presence of the unique PTA ligand, with an enhanced intensity of the second peak correlating with the increased ligand density around the metal center.

EXAFS data analysis at the Cu K edge was achieved for complexes **1**–**7** by selecting the most relevant contributions (single scattering SS or multiple scattering MS) from the DFT-optimized local atomic structure as previously described [15,18,22,31,47,52]. The k^2^-weighted experimental data k^2^χ(k) and best fit results are shown for copper complexes **1**–**3** and **4**–**7** in Figure 5c,d, respectively. After a trial-and-error procedure to minimize the number of free variables, three single scattering (SS) and one multiple scattering (MS) contributions were found to be statistically significant for each of the complexes **1**–**5**. For complexes **6** and **7**, MS were found not statistically relevant, so only three SS contributions were included in the analysis.

In all samples, the local Cu coordination involves the bidentate bis(pyrazol-1-yl)acetate ligand, resulting in common scattering paths among the samples, namely the single scattering from the first and second nitrogen neighbors (N1 and N2), respectively, around 2.0 Å and 3.0 Å from the Cu absorber. Key differences arose in the EXAFS refinement of the samples due to the different ligand coordination, with complex **1** featuring the signal (SS) from two Cl neighbors at a distance of 2.14 Å. Instead, complex **2** presented the signal (SS) from the two Br neighbors at 2.38 Å, and complex **3** showed the contribution (SS) from two O neighbors at 2.43 Å. Cu^I^ complexes **4–7** showed an SS signal with P, with a distance around 2.15 Å for complexes **4** and **5** and around 2.25 Å for complexes **6** and **7**. Additionally, the analysis of complexes **1**, **2**, and **5** required a triple scattering contribution due to the almost collinear scattering between the first nitrogen neighbor and the carbon atom directly bound to it in the pyrazole ring (N1-C2) with a distance of ~4.35 Å. Complex **3** required a quadruple scattering contribution involving the first neighboring nitrogen and the carbon atom opposite in the pyrazole ring (N1-C5-N1) at a distance of 4.27 Å. Lastly, complex **4** required a multiple scattering path characteristic of the triphenylphosphine ligand, involving the phosphorous neighbor in the first shell of coordination and the carbon atom directly bound to that, in the ipso position in the aromatic ring (P-C3) at a distance of 3.53 Å. Multiplicities across all scattering contributions were found to be in agreement with theoretical models and were fixed throughout the analysis to reduce parameter correlation. Best fit results are reported in Table 1.

### 2.5. Stability Analysis for Complexes ***1**–**7*** and Ligand L^Ad^ in DMSO Solution

Complexes **1**–**7** and related ligand L^Ad^ were analyzed by UV-Vis spectroscopy at 250–700 nm to assess their stability in DMSO. This study aimed to evaluate changes in absorbance over time in order to determine whether the complexes were suitable for subsequent cytotoxicity tests. The analysis was conducted by dissolving 2 mmol of each compound in 25 mL of DMSO and spectra were recorded at time 0 and after 24, 48, and 72 h. The collected spectra for all compounds are reported in the Supporting Information Figure S27, and their analysis showed that they remained stable in DMSO solution up to 72 h.

### 2.6. Biological Studies

#### 2.6.1. Cell Viability

The effect of the newly synthesized copper complexes on cell viability was preliminarily assessed in U87 MG and LN18 GBM cell lines using the MTT assay. Both cell lines were treated for up to 72 h with seven increasing concentrations (0–50 µM) of each compound, the uncoordinated ligand L^Ad^ and cisplatin, as a reference metal-based drug. IC_50_ values, determined after 72 h of treatment, are reported in Table 2.

**Figure 6:**
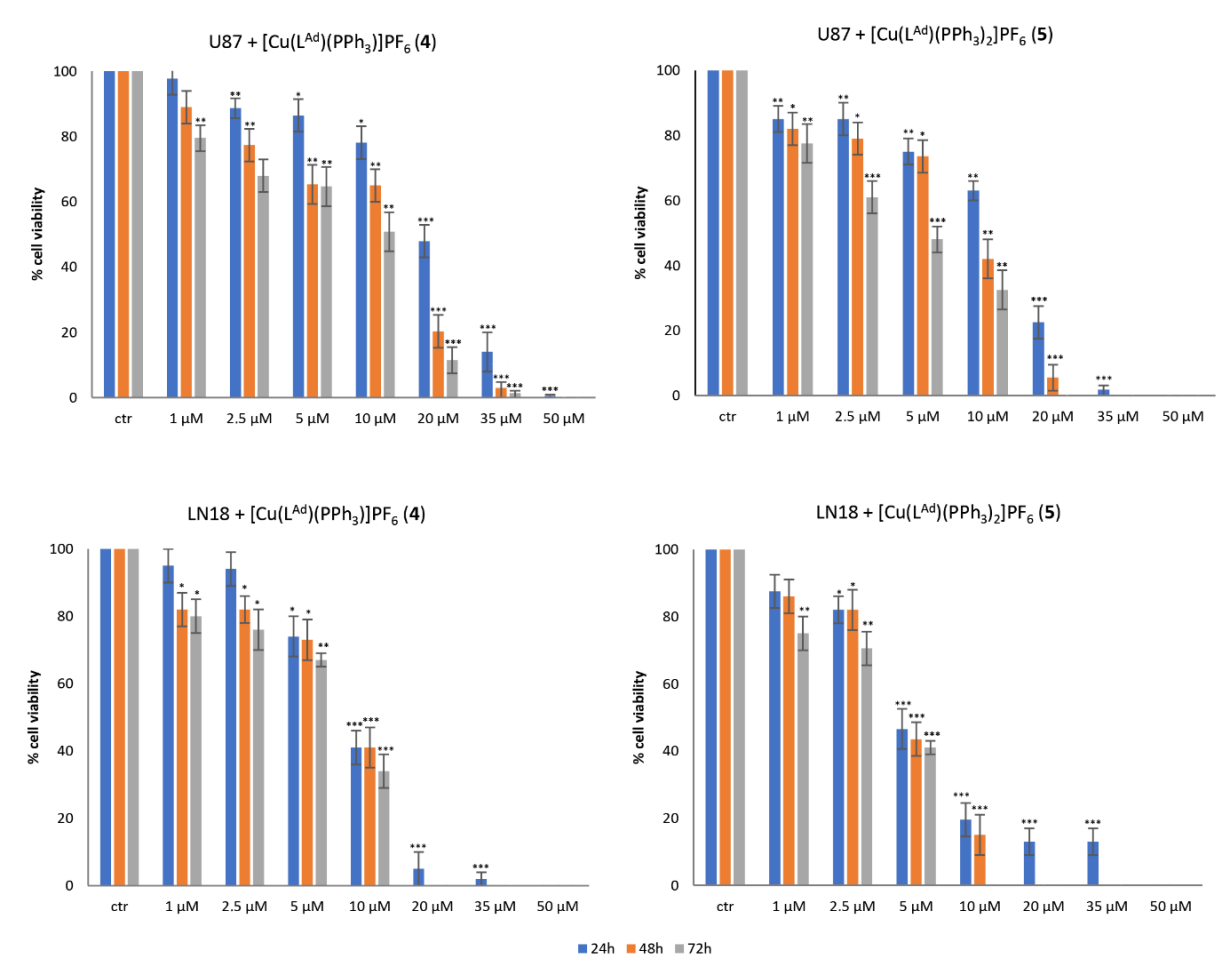
U87 MG and LN18 cell viability evaluated by MTT test. Cells were incubated with copper compounds **4** and **5** at different concentrations (up to 50 µM) for 24, 48, and 72 h. Data are expressed as % of cell viability with respect to the control (set at 100% value). Results are expressed as mean ± SD values from three independent experiments. Statistical analysis was performed comparing the % of cell viability of each treated vs. control sample. * *p* < 0.05, ** *p* < 0.01, *** *p* < 0.001 (two-tailed Student’s *t*-test).

Cu^II^ complexes **1**–**3** and Cu^I^ complexes with PTA co-ligands (**6** and **7**) did induce no or very low cell viability reduction (≤ 30%, Figure S28). In contrast, compounds **4** and **5** exerted a pronounced cytotoxic effect at micromolar concentrations, lower than cisplatin, in both cell lines (Figure 6). This enhanced activity might be attributed to the high cell permeability of these complexes, likely related to the presence of lipophilic PPh_3_ co-ligands in their structures.

#### 2.6.2. Intracellular Reactive Oxygen Species (ROS) Level Evaluation

**Figure 7:**
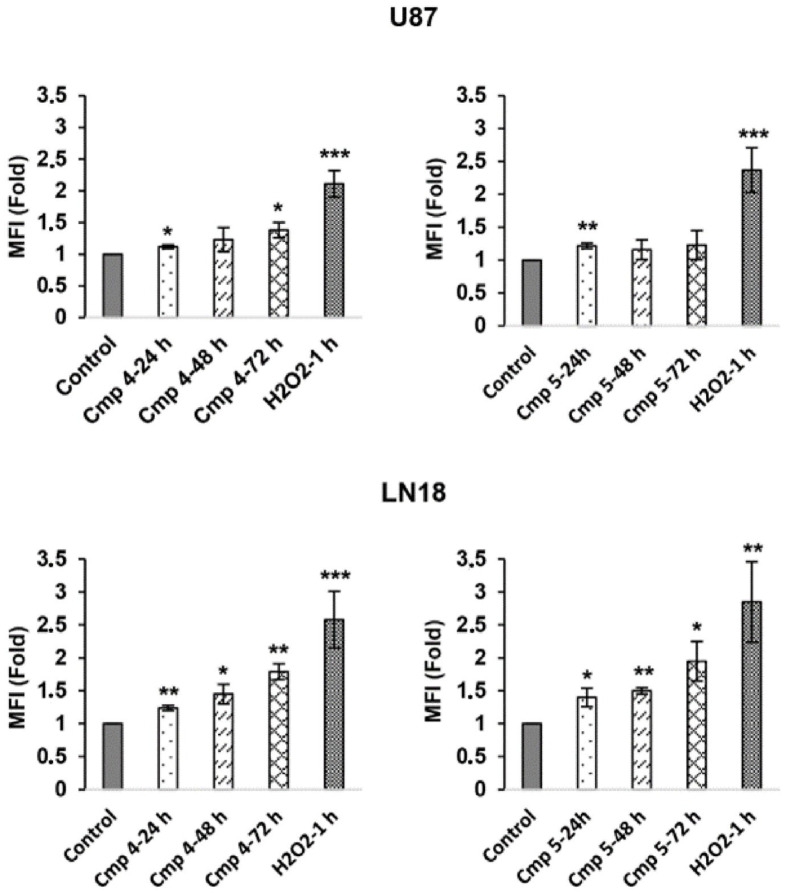
Evaluation of ROS production by flow cytometry. U87 MG and LN18 cell lines were treated with the IC_50_ concentration of compounds **4** (10.0 or 8.0 µM for U87 MG and LN18 cells, respectively) and **5** (5.0 and 3.5 µM for U87 MG and LN18 cells, respectively) for 24, 48, and 72 h. As a positive control, cells were treated with H_2_O_2_ (final concentration 100 µM) for 1 h. Samples were then pulsed with DCFDA and immediately analyzed. Data are expressed as fold obtained by the ratio of mean fluorescence intensity (MFI) values from treated cells and those from control ones. Results represent the mean ± SD of three independent experiments. Statistical analysis was performed comparing the MFI values of each treated vs. control sample. * *p* < 0.05, ** *p* < 0.01, *** *p* < 0.001 (two-tailed Student’s *t*-test).

Copper ions are known to play a direct role in ROS generation and oxidative stress response [53,54]. Thus, intracellular ROS levels were analyzed in U87 MG and LN18 cell lines treated with compounds **4** and **5** at their respective IC_50_ doses after 24, 48, and 72 h. Carboxy-2′,7′-dichlorofluorescein diacetate (DCFDA) was used to detect intracellular ROS levels following its cleavage by intracellular esterases and oxidation to green, fluorescent DCF by ROS. Incubation with Cu compounds led to increased ROS levels in both cell lines, with a more pronounced and sustained effect observed in LN18 cells at all time points (Figure 7).

#### 2.6.3. Intracellular GSH Level Evaluation

**Figure 8:**
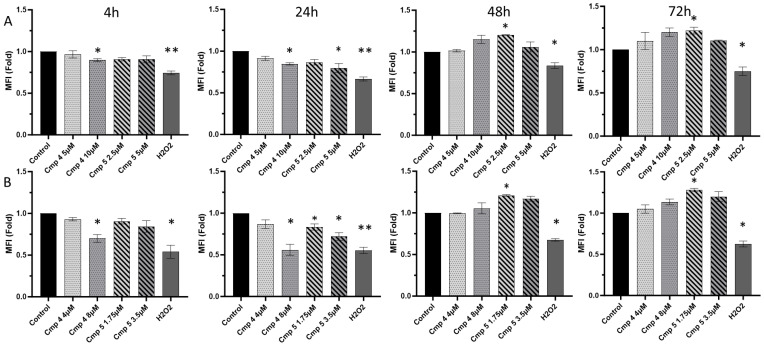
Intracellular GSH levels in U87 MG (**A**) and LN18 cells (**B**). Cells were treated with compounds **4** (5 and 10 μM for U87 MG; 4 and 8 μM for LN18) and **5** (2.5 and 5 μM for U87 MG; 1.75 and 3.5 μM for LN18) for 4, 24, 48, and 72 h. As a positive control, cells were treated with H_2_O_2_ (final concentration 100 µM) for 1 h. GSH levels were measured using MCB staining, and mean fluorescence intensity (MFI) values were calculated with CytExpert v2.4 software. At least 10,000 events were acquired per sample. Data are expressed as fold change relative to control cells as mean ± SD from three independent experiments. Statistical significance vs. untreated control: * *p* < 0.05, ** *p* < 0.01 (two-tailed Student’s *t*-test). Cells were gated on FSC-A vs. SSC-A to exclude debris, followed by doublet discrimination and exclusion of non-viable cells prior to analysis. The detailed gating strategy is shown in Figure S29.

To further investigate the redox imbalance induced by Cu compounds **4** and **5**, intracellular levels of reduced glutathione (GSH) were assessed using monochlorobimane (MCB), a well-established fluorescent probe for GSH quantification [55]. U87 MG and LN18 cell lines were treated with compounds **4** (5 and 10 μM for U87 MG; 4 and 8 μM for LN18) and **5** (2.5 and 5 μM for U87 MG; 1.75 and 3.5 μM for LN18) for 4, 24, 48, and 72 h. In U87 MG cells (Figure 8, panel A), a statistically significant reduction in GSH levels was observed after 4 h of treatment with compound **4** at its IC_50_ dose (10 μM). At 24 h, GSH depletion remained significant for compound **4** (10 μM) and a significant reduction was also observed for compound **5** at its IC_50_ dose (5 μM). No significant changes were detected at later time points, when GSH levels tended to stabilize or slightly increase. In contrast, LN18 cells (Figure 8, panel B) exhibited a significant and time-dependent decrease in GSH levels in response to both compounds. Compound **4** induced a statistically significant reduction as early as 4 h, which became more pronounced at 24 h, especially at the higher concentration (8 μM, IC_50_ dose). Similarly, compound **5** caused a slight decrease in GSH levels at 4 h, followed by a statistically significant reduction at 24 h at both 1.75 and 3.5 μM concentrations. Partial recovery of GSH levels was observed at 48 and 72 h, suggesting the activation of compensatory antioxidant mechanisms. These findings correlate with the observed ROS accumulation and support the conclusion that LN18 cells are more sensitive to redox imbalance than U87 MG cells upon exposure to compounds **4** and **5**.

#### 2.6.4. Cell Cycle Assay

**Figure 9:**
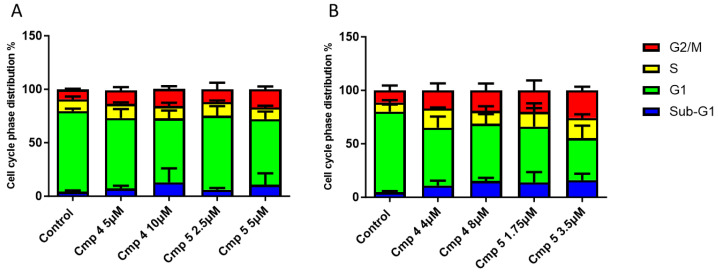
Effects on cell cycle distribution in U87 MG (**A**) and LN18 (**B**) cells. Cell cycle profiles were determined by DNA content analysis using flow cytometry with propidium iodide (PI) staining. Cells were treated with compounds **4** (5 and 10 μM for U87 MG; 4 and 8 μM for LN18) and **5** (2.5 and 5 μM for U87 MG; 1.75 and 3.5 μM for LN18) for 72 h. The percentages of cells in G0/G1, S, and G2/M phases were quantified using Kaluza Analysis software version 2.4. At least 10,000 events were acquired per sample. Values represent the mean ± SD of three independent experiments. Cells were gated on FSC-A vs. SSC-A to exclude debris, followed by doublet discrimination and exclusion of non-viable cells prior to analysis. The detailed gating strategy is shown in Figure S29.

We performed cell cycle analysis to further investigate the mechanism by which copper compounds affect GBM cell viability. U87 MG and LN18 cells were treated with compounds **4** (5 and 10 μM for U87 MG; 4 and 8 μM for LN18) and **5** (2.5 and 5 μM for U87 MG; 1.75 and 3.5 μM for LN18) for 24, 48, and 72 h. No significant changes were observed at 24 h. At 48 h of treatment with the highest concentrations of compounds **4** and **5** caused a moderate increase in the G2/M population in both U87 MG (17% vs. 10% in controls) and LN18 cells (23% vs. 15% in controls), as reported in the Supporting Information Figure S30. At 72 h, both cell lines showed a marked accumulation in the G2/M phase at the highest tested doses of compounds **4** and **5** (Figure 9). In U87 MG cells, the G2/M population increased to approximately 19% compared with 9% in untreated controls. In LN18 cells, the effect was more pronounced, with about 32% of cells in G2/M compared with 10% in controls.

#### 2.6.5. Cell Death Assessment

**Figure 10:**
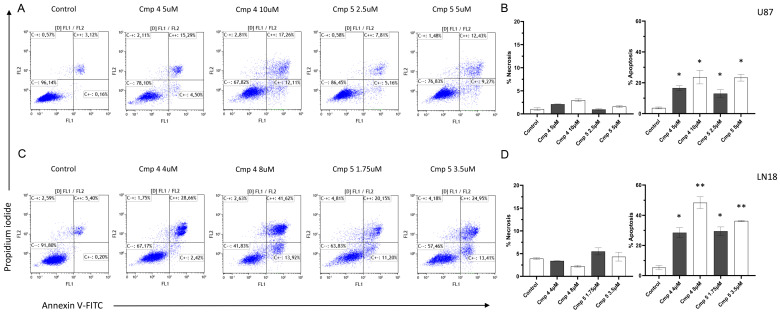
Cell death assessment on U87 MG and LN18 cells. Apoptosis was analyzed by flow cytometry after 72 h of treatment with compounds **4** and **5** in U87 MG (**A**,**B**) and LN18 (**C**,**D**) cells, using Annexin V (AV) and propidium iodide PI staining. Quantification of apoptotic cells is presented as mean ± SD from at least three independent experiments in panels (**B**,**D**), while representative dot plots are shown in panels (**A**,**C**). The numbers indicate the percentages of AV-negative/PI-positive (necrotic cells, upper left quadrant), AV-positive/PI-negative (early apoptotic cells, lower right quadrant), and AV-positive/PI-positive (late apoptotic cells, upper right quadrant). At least 10,000 events were acquired per sample. Statistical significance vs. untreated control was evaluated using a two-tailed Student’s *t*-test (* *p* < 0.05, ** *p* < 0.01). Cells were gated on FSC-A vs. SSC-A to exclude debris, followed by doublet discrimination and exclusion of non-viable cells prior to analysis. The detailed gating strategy is shown in Figure S29.

To further investigate the nature of the cytotoxic response, we next examined cell death induction. U87 MG and LN18 cells were treated with selected concentrations of both compounds (compound **4**: 5 and 10 μM for U87 MG; 4 and 8 μM for LN18; compound **5**: 2.5 and 5 μM for U87 MG; 1.75 and 3.5 μM for LN18) for 24, 48, and 72 h. Cell death was assessed by Annexin V-FITC/PI staining followed by flow cytometry analysis. As shown in Figure 10, after 72 h of treatment, the time point at which the apoptotic effect was most pronounced, both U87 MG (panels A, B) and LN18 cells (panels C, D) displayed significant increases in the apoptotic cell population compared with untreated controls (5%). In U87 MG cells, compound **4** induced apoptosis in 20% and 29% of the population at 5 and 10 μM, respectively, while compound **5** led to 13% and 22% apoptosis at 2.5 and 5 μM, respectively. In LN18 cells, compound **4** induced apoptosis in 31% and 56% of cells at 4 and 8 μM, respectively, while compound **5** induced 31% and 38% apoptosis at 1.75 and 3.5 μM, respectively. Overall, these results suggest that the cytotoxic effects of compounds **4** and **5** involve both cell cycle arrest and induction of cell death.

#### 2.6.6. Western Blot Analysis

**Figure 11:**
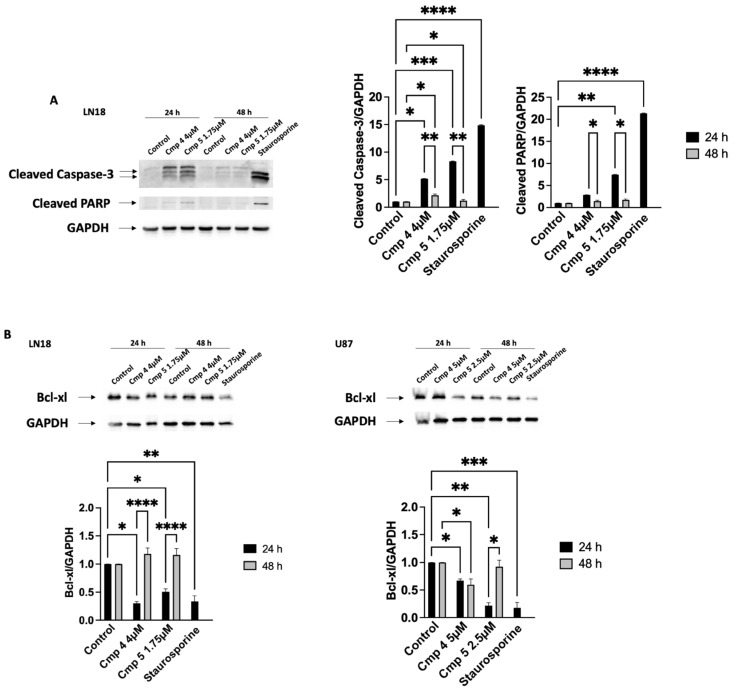
Western blot analysis of key proteins involved in apoptotic signaling and its regulation. Protein expression analysis after 24 and 48 h of treatment with compounds **4** and **5** in LN18 and U87 MG cells. (**A**) indicates the expression level of cleaved caspase-3, and cleaved PARP-1 in LN18 cells. Data were normalized to the level of GAPDH protein expression. (**B**) indicates the expression level of Bcl-xl in LN18 and U87 MG cells. Folds (mean ± SD of three experiments) represent changes compared with control cells. Statistical significance was assessed by two-way ANOVA followed by Tukey’s multiple comparisons test: * *p* < 0.05, ** *p* < 0.01, *** *p* < 0.001, **** *p* < 0.0001.

Western blot analysis was performed to determine the levels of cleaved caspases and cleaved PARP-1 in GBM cells cultured with or without both compounds (compound **4**: 5 μM for U87 MG; 4 μM for LN18; compound **5**: 2.5 μM for U87 MG; 1.75 μM for LN18) for 24 and 48 h. Cells treated with 250 nM staurosporine served as positive control. Data were normalized by the level of GAPDH expression used as the internal control. In LN18 cells, treatment with Cu compounds increased the levels of cleaved caspase-3 and cleaved PARP-1 after 24 h, whereas this effect was less evident after 48 h (Figure 11A).

Bcl-xL levels were significantly reduced at 48 h, following a trend similar to that observed for the other apoptotic markers (Figure 11B). These results indicate that compounds **4** and **5** induced caspase-dependent apoptosis in LN18 cells. In contrast, in U87 MG cells, although Bcl-xL decreased in a dose-dependent manner (Figure 11B), no cleavage of caspase-3 or PARP was observed (Figure S31), and other effector caspases, such as caspase-6 and -7, also showed no evidence of activation. These results suggest that both complexes induce cell death in U87 MG cells through a caspase-independent pathway. Differences in drug response are not uncommon in glioma treatment due to tumor heterogeneity [56].

#### 2.6.7. Cell Morphological Studies

To evaluate the effect of compounds **4** and **5** on GBM cell morphology, we performed scanning electron microscopy (SEM), immunofluorescence (IF), and phase-contrast microscopy on U87 MG and LN18 cells after 24 h of treatment at IC_50_ concentrations (compound **4**: 10 μM for U87 MG; 8 μM for LN18; compound **5**: 5 μM for U87 MG; 3.5 μM for LN18).

**Figure 12:**
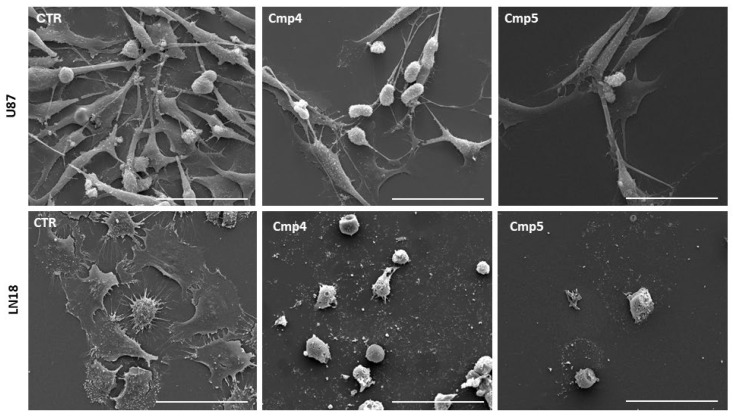
SEM analysis of U87 and LN18 cells treated with compounds **4** and **5**. After 24 h of treatment at IC_50_ doses, GBM cells showed rounding and shrinkage, detachment and loss of filopodia compared to untreated controls. Scale bar: 50 µm.

SEM analysis (Figure 12) showed that control cells had a flat, spread morphology with extended filopodia and lamellipodia. In contrast, cells treated with compounds **4** and **5** exhibited typical signs of cytotoxic stress, including shrinkage, membrane blebbing, and loss of adhesion. LN18 cells treated with compound **5** showed more severe alterations, with most cells detached or rounded in shape.

Phase-contrast microscopy (Figure S32) confirmed these morphological alterations. Control cells displayed normal shape and confluency, while treated cells showed rounding, detachment, and reduced density, more evident after compound **5** treatment, especially in LN18.

Particularly, in compound **5**-treated LN18 cells, we observed loss of stress fibers and alterations in cytoskeletal architecture, accompanied by fewer adherent cells and early signs of nuclear condensation, consistent with early apoptotic events (Figure S33).

Overall, these morphological analyses align with cell viability, flow cytometry, and confocal microscopy data, suggesting that compounds **4** and **5** can disrupt cytoskeletal integrity and induce apoptosis-like changes, with LN18 cells showing higher sensitivity.

#### 2.6.8. Laser Scanning Confocal Microscopy Analysis

To further investigate the mechanism underlying the cytotoxic effects of Cu-based complexes, confocal microscopy analysis was carried out in LN18 cells treated with compound **5** at a sub-cytotoxic concentration (0.9 µM) for 6 h. LN18 cells were selected based on their higher sensitivity to treatment and their pronounced activation of the intrinsic, caspase-dependent apoptotic pathway, as indicated by caspase-3 and PARP-1 cleavage (Figure 11) and persistent oxidative stress. These features suggest significant mitochondrial involvement, making LN18 cells a relevant model to investigate mitochondrial events such as BAX translocation and cytochrome c release. Short incubation times and low-dose conditions were used to study early apoptotic events preceding cell death.

**Figure 13:**
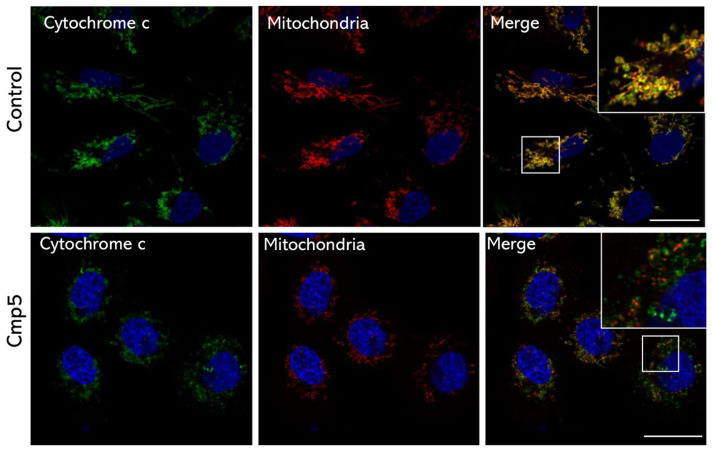
Confocal microscopy analysis of cytochrome c release in LN18 cells. LN18 cells were treated with compound **5** (0.9 µM) for 6 h and stained for cytochrome c (green), mitochondria (red), and nuclei (blue). In control cells, cytochrome co-localizes with mitochondria, as shown by the strong yellow signal in the merged images. Upon treatment with compound **5**, cytochrome c is released from mitochondria, indicated by a loss of co-localization and diffuse cytoplasmic staining. Insets show magnified views of selected areas. Scale bar: 20 µm.

In control samples, cytochrome c displayed a clear mitochondrial localization pattern, as indicated by strong overlap between the green (cytochrome c) and red (mitochondria) fluorescence channels, resulting in a clear yellow signal (Figure 13 insert in upper panel merge). Upon treatment with compound **5** (0.9 µM), cytochrome c markedly delocalized from mitochondria to the cytoplasm, as shown by loss of colocalization and separation of the green and red signals (insert). This redistribution is consistent with mitochondrial outer membrane permeabilization [57,58].

**Figure 14:**
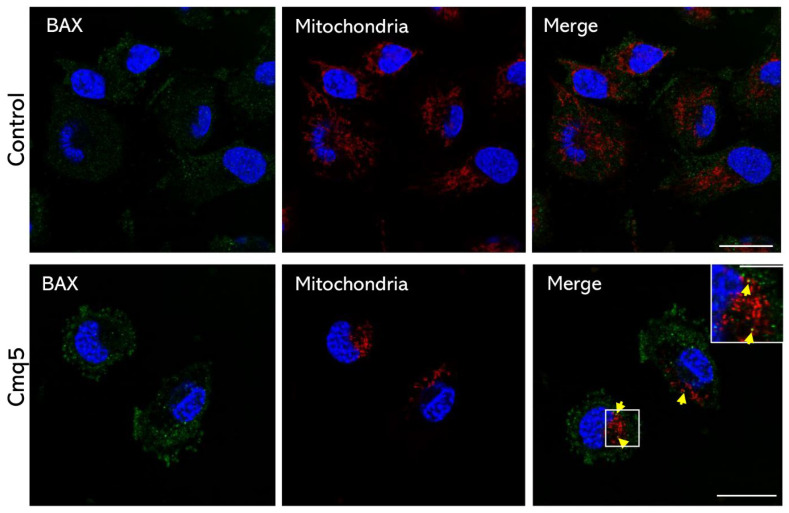
Confocal microscopy analysis of BAX mitochondrial translocation in LN18 cells. LN18 cells were treated with compound **5** (0.9 µM) for 6 h and immunostained for BAX (green), mitochondria (red), and nuclei (blue). In control cells, BAX displays a diffuse cytoplasmic distribution. After treatment with compound **5**, BAX translocates to the mitochondria, evidenced by increased co-localization (yellow signal in the merged image, highlighted by arrowheads). Insets show magnified areas of BAX colocalization with mitochondria. Scale bar: 20 µm.

Consistently, analysis of BAX localization revealed a diffuse cytoplasmic distribution in control cells (Figure 14). After 6 h of exposure to compound **5**, BAX partially translocated to mitochondria, as indicated by discrete yellow puncta (Figure 14 merge arrows and insert), reflecting colocalization with mitochondrial markers. Although less pronounced than cytochrome c release, this pattern suggests an early activation of the mitochondrial apoptotic cascade, in line with previous studies on BAX-mediated apoptosis [59,60].

Nuclear morphology further supported these findings. Hoechst staining of control nuclei showed intact, uniform structures, whereas treated cells exhibited early signs of nuclear condensation [61]. Although these alterations were not extensive at the 6-h timepoint, they are consistent with early apoptotic processes. Collectively, these microscopy results corroborate the MTT cytotoxicity and flow cytometry results, reinforcing the hypothesis that compound **5** induces cell death via mitochondrial disruption. Specifically, mitochondrial clustering and rounding, together with oxidative stress, likely contribute to loss of mitochondrial integrity [62]. The observed cytochrome c release into the cytosol indicates the mitochondrial membrane permeabilization and activation of apoptosis. Likewise, partial mitochondrial localization of BAX suggests that compound **5** activates early upstream signals of the intrinsic apoptotic pathway. These results are consistent with reports describing copper complexes as modulators of oxidative stress and mitochondrial function in cancer cells [60,63]. Overall, our data strongly suggest that short exposure of LN18 cells with sub-cytotoxic concentrations of compound **5** triggers early mitochondrial dysfunction, BAX activation, and cytochrome c release, key events in apoptosis induction. These observations align with Annexin V staining and Western blot data, which also support early apoptotic commitment following treatment. These insights shed light on the mechanism of action of Cu-based complexes and support their potential as promising agents in GBM therapy.

#### 2.6.9. Overall Discussion of the Biological Results

GBM is a poorly differentiated and highly proliferative primary brain tumor characterized by marked heterogeneity, invasive growth, and poor prognosis [64]. Systemic therapy mainly consists of TMZ-based chemotherapy. The alkylating agent TMZ induces its cytotoxic effect mainly through the methylation of guanine residues in DNA at the O6 position. This adduct can be repaired by O6-methylguanine-DNA-methyltransferase (MGMT), which is heterogeneously expressed in GBM. Methylation of the MGMT promoter leads to epigenetic transcription silencing, and MGMT hypermethylation can confer sensitivity to TMZ treatment [65]. Unfortunately, GMB prognosis remains poor due to low sensitivity to TMZ treatments, tumor recurrence, and stem cell population, among the main resistance mechanisms, with a median survival of about 15 months [66]. Therefore, additional research is needed to develop new therapeutic strategies to improve outcomes for GBM patients.

Copper ions are increasingly recognized as regulators of cancer cell survival through modulation of redox balance and activation of cell-death pathways such as cuproptosis [10], offering promising opportunities for the development of redox-targeted therapies. In this study, we investigated the effects of Cu compounds **4** and **5** in two GBM cell lines, U87 MG and LN18, which differ in molecular features and sensitivity to radio- or chemotherapy. In addition to differences in MGMT expression (low level of MGMT due to partial methylation of the promoter in TMZ-sensitive U87 cells; upregulation of MGMT in TMZ-resistant LN18 cells) [67], recent studies identified additional resistance-associated pathways in GBM, including ROS detoxification or ferroptosis/autophagy-related regulatory mechanisms [68].

Our results indicate that both Cu compounds **4** and **5** induced oxidative stress, cell cycle arrest, and cell death in GBM cell models used in this study. Interestingly, LN18 cells exhibited a significantly stronger response across assays compared with U87 MG cells. Regarding ROS and GSH analyses, both compounds induced ROS accumulation, although to different extents. LN18 cells exhibited gradual ROS accumulation that remained elevated throughout the observation period while U87 MG cells showed less sustained increase. Consistent with ROS generation, both cell lines showed an initial decrease in GSH levels at 4 and 24 h, suggesting early oxidative stress and depletion of antioxidant capacity. At later time points (48 h), levels increased again, suggesting upregulation of antioxidant enzymes and/or de novo GSH synthesis. However, LN18 cells maintained lower GSH levels than U87 MG cells, mainly at 24 h, indicating reduced tolerance to copper-induced oxidative stress. The accumulation of ROS and the slower recovery of GSH in LN18 cells suggest limited redox adaptability. Conversely, ROS induction followed by GSH restoration in U87 MG cells is consistent with a more effective antioxidant response. These different redox behaviors may reflect intrinsic genetic differences between the two models, such as their distinct PTEN status. PTEN deficiency, as in U87 MG cells, has been associated with increased tolerance to oxidative stress and may contribute to their higher resistance compared with LN18 [69]. In addition, U87 MG cells have been reported to express elevated levels of COX-2, a key mediator of cellular antioxidant defense through upregulation of antioxidant signaling pathways, such as Nrf2. These mechanisms may contribute to partial resistance of U87 MG cells to TMZ, despite their low MGMT, and to other oxidative stress-inducing agents [70,71]. Cell cycle analysis also revealed a greater effect in LN18 than U87 MG cells, with both compounds inducing G2/M arrest. This observation suggests that oxidative stress-induced DNA damage may lead to the G2/M arrest more effectively in LN18 than in U87 MG cells. In addition, Annexin V assays confirmed dose-dependent induction of cell death by both compounds, with significantly higher apoptotic rates in LN18 cells. Western blot analysis confirmed the differential apoptotic responses observed in LN18 and U87 MG cells: in LN18 cells, both copper compounds induced significant caspase-3 activation and PARP-1 cleavage after 24 h, consistent with the engagement of a caspase-dependent apoptotic pathway [59,60]. This effect was less pronounced after 48 h, potentially reflecting adaptive cellular responses or partial loss/elimination of apoptotic cells. The concomitant decrease in Bcl-xL further supports involvement of the intrinsic mitochondrial pathway. In contrast, U87 MG cells showed only a slight reduction in Bcl-xL levels, with no detectable caspase-3 or PARP-1 cleavage or activation of other effector caspases (6 and 7), suggesting induction of cell death through a caspase-independent pathway.

Morphological analyses further confirmed the different effects produced by compounds **4** and **5** on the two cell lines. SEM, phase contrast, and confocal microscopies highlighted that LN18 cells were more sensitive to cytotoxic stress than U87 MG, with rounding, detachment, cytoskeletal disruption, and early nuclear condensation. After brief exposure to sub-cytotoxic doses of compound **5**, confocal imaging revealed early apoptosis mechanisms, including partial BAX translocation into the mitochondria and cytochrome c release [59,60], hallmark events of the intrinsic apoptotic pathway [72,73]. Together with oxidative stress, these results suggest that the intrinsic apoptotic pathway is robustly triggered only in LN18 cell, in agreement with the observed caspase-3 and PARP-1 cleavage and the known ability of copper to disrupt mitochondrial membrane potential, modulate oxidative phosphorylation, and induce ROS-dependent cell death.

The selection of LN18 cells for detailed confocal studies was supported by their pronounced activation of the mitochondrial, caspase-dependent apoptotic pathway, including caspase-3 and PARP-1 cleavage, persistent ROS accumulation, limited GSH recovery, and pronounced G2/M arrest, which together make this model particularly suitable for investigating upstream mitochondrial events using confocal microscopy. Moreover, their higher resistance of LN18 cells to standard therapies, such as TMZ, reinforces their translational relevance for studying novel redox-targeting agents in GBM [57,58,74,75,76].

Overall, our results indicate that Cu compounds **4** and **5** induced G2/M phase arrest and mitochondrial dysfunction-associated oxidative stress with more pronounced effects, in LN18 cells than U87 MG cells. Moreover, caspase-dependent intrinsic apoptosis was observed only in LN18 cells. The differential sensitivity of LN18 and U87 MG likely reflects differences in antioxidant capacity, mitochondrial function, and apoptotic signaling. These data highlight the potential of Cu complexes to selectively target redox imbalances in resistant GBM phenotypes. The heightened sensitivity of the chemoresistant phenotype to copper-induced redox stress underlines a critical therapeutic principle: cancer cells that evade conventional DNA-damaging agents may be vulnerable to alternative mechanisms.

## 3. Materials and Methods

### 3.1. Chemistry

#### 3.1.1. Materials and General Methods

All reagents used for the synthesis of ligands and complexes were purchased from Sigma–Aldrich (St. Louis, MO, USA) and used without further purification. Elemental analyses (C, H, N, S) were performed in-house with the Fisons THERMO Fisher Flash 2000 instrument. Melting points were taken on an SMP3 Stuart Scientific Instrument (Stuart Scientific, North Tyneside, UK). IR spectra were recorded from 4000 to 200 cm^−1^ with a Perkin–Elmer Frontier FT-IR Instrument (Perkin–Elmer, Waltham, MA, USA). IR annotations used: br = broad, m = medium, s = strong, sh = shoulder, vs. = very strong; vw = very weak; w = weak. ^1^H-,^31^P-, and^13^C-NMR spectra were recorded on an Ascend 500 Bruker spectrometer (500.1 MHz for ^1^H, 202.5 MHz for ^31^P, 125.8 MHz for ^13^C). Chemical shifts in ppm for ^1^H- and ^13^C-NMR spectra are relative to internal standard Me_4_Si. ^31^P-NMR chemical shifts were referenced to an 85% H_3_PO_4_ standard. The ^31^P-NMR chemical shifts were acquired with ^1^H decoupling. NMR annotations used: br = broad, d = doublet, m = multiplet, q = quartet, s = singlet, sept = septet. Electrospray ionization-mass spectra (ESI-MS) were obtained in positive- and negative-ion (ESI-MS(+) and ESI-MS(-)) mode on a Series 1100 MSD detector HP spectrometer (Agilent Technologies, Santa Clara, CA, USA), using a methanol or acetonitrile mobile phase. The compounds were added to reagent grade CH_3_OH or CH_3_CN to give solutions of approximate concentration 0.1 mM. These solutions were injected (1 µL) into the spectrometer via an HPLC HP 1090 Series II fitted with an autosampler. The pump delivered the solutions to the mass spectrometer source at a flow rate of 300 µL min^−1^, and nitrogen was employed both as a drying and nebulizing gas. Capillary voltages were typically 4000 V and 3500 V for the positive- and negative-ion mode, respectively. Confirmation of all major species in this ESI-MS study was aided by comparison of the observed and predicted isotope distribution patterns, the latter calculated using the IsoPro 3.0 computer program.

#### 3.1.2. Synthesis of [Cu(L^Ad^)Cl_2_] (**1**)

The ligand L^Ad^ (0.500 mmol, 0.163g) was added to a solution of copper chloride (0.500 mmol, 0.085 g) in CH_3_CN (30 mL). The reaction was stirred for 24 h at room temperature, and for the last 2 h, it was refluxed. The light blue solution obtained was filtered, and the mother liquors were evaporated at reduced pressure. The residue was washed with diethyl ether and filtered, giving the light blue complex [(L^Ad^)CuCl_2_] in 55% yield (0.126 g). Solubility: CH_3_OH, DMSO. Mp: 200–204 °C. FT-IR (cm^−1^): 3284wbr (N-H); 3117w, 3106w, 2904m (C-H); 2854w; 1664vs (C=O); 1566s (C=C/C=N); 1516w, 1451w, 1410m, 1375w, 1359m, 1332m, 1284s, 1251w, 1235m, 1205w, 1114m, 1065s, 989m, 915w, 901w, 857m, 834m, 818w, 768vs, 717w, 667w, 639wbr, 612vs, 599m, 572m, 448wbr, 387mbr, 372wbr, 348wbr, 336wbr, 320sbr, 306sbr, 291sbr; 281s (CuCl); 274mbr, 252m, 246s, 231s, 225s, 202vs. ESI-MS(+) (major positive ions, CH_3_CN): *m*/*z* (%), 423 (30) [(L^Ad^)CuCl]^+^, 748 (20) [(L^Ad^)_2_CuCl]^+^. ESI-MS(-) (major negative ions, CH_3_CN): *m*/*z* (%): 360 (100) [L^Ad^ + Cl]^−^, 495 (5) [(L^Ad^)CuCl_2_ + Cl]^−^. Elemental analysis (%) calculated for C_18_H_23_Cl_2_CuN_5_O: C 47.01, H 5.04, N 15.23; found: C 47.19, H 5.04, N 15.42.

#### 3.1.3. Synthesis of [Cu(L^Ad^)Br_2_] (**2**)

The ligand L^Ad^ (0.500 mmol, 0.163 g) was added to a solution of copper bromide (0.500 mmol, 0.110 g) in CH_3_CN (30 mL), and the reaction was stirred for 24 h at room temperature. The lime green solution was filtered, giving a brownish complex [(L^Ad^)CuBr_2_] in 90% yield (0.247 g). Mp.: 268–270 °C. Solubility: CH_3_OH, EtOH, CH_3_CN, DMSO. FT-IR (cm^−1^): 3265mbr (N-H); 3113w, 3089w, 2905m (C-H); 2853m; 1663vs (C=O); 1555s (C=C/C=N); 1516m, 1451m, 1408s, 1360m, 1330w, 1310m, 1283s, 1252w, 1236m, 1207m, 1098s, 1065s, 989s, 916m, 857m, 832m, 818w, 757vs, 645w, 613vs, 598s, 573m, 450m, 408mbr, 387mbr, 339sbr, 324mr, 247s, 231mbr; 220vs (CuBr). ESI-MS(+) (major positive ions, CH_3_OH): *m*/*z* (%): 135 (70) [ADM]^+^, 469 (30) [(L^Ad^)CuBr]^+^, 794 (30) [(L^Ad^)_2_CuBr]^+^. ESI-MS(-) (major negative ions, CH_3_OH), *m*/*z* (%): 304 (100) [CuBr_3_]^−^. Elemental analysis (%) calculated for C_18_H_23_Br_2_CuN_5_O: C 40.48, H 4.65, N 12.42; found: C 40.11, H 4.30, N 12.49.

#### 3.1.4. Synthesis of [Cu(L^Ad^)_2_Br_2_] (**3**)

Copper bromide (0.500 mmol, 0.110 g) and the ligand L^Ad^ (1.000 mmol, 0.326 g) were dissolved in CH_3_CN (30 mL); the reaction was stirred for 24 h at room temperature. The dark grey solution was filtered, giving the grey precipitate [(L^Ad^)_2_CuBr_2_] in 88% yield (0.385 g). Mp: 240–245 °C. Solubility: CH_3_OH, CH_2_Cl_2_, CHCl_3_, CH_3_CN, DMSO. FT-IR (cm^−1^): 3302w (N-H); 3184wbr, 3116w, 3014wbr, 2903mbr, 2851w (C-H); 2664w, 2249w; 1665vs (C=O); 1561m (C=C/C=N); 1513wbr, 1458wbr, 1407m, 1379w, 1358w, 1331w, 1290m, 1273w, 1236m, 1206w, 1190w, 1099m, 1089m, 1064m, 993m, 916w, 855m, 837m, 816w, 780vs, 773vs, 717w, 681m, 655w, 639w, 614s, 604m, 574m, 555vw, 521wbr, 496vw, 480vw, 480w, 451w, 426vw, 415w, 398m, 377m, 355m, 303m, 286m, 274m; 242s (CuBr); 227w. ESI-MS(+) (major positive ions, CH_3_OH) *m*/*z* (%): 135 (90) [2Pz]^+^, 358 (100) [(L^Ad^)_2_Cu]^2+^, 348 (5) [L^Ad^ + Na]^+^, 469 (30) [(L^Ad^)CuBr]^+^, 712 (30) [(L^Ad^)_2_Cu]^+^, 794 (100) [(L^Ad^)_2_CuBr]^+^. ESI-MS(-) (major negative ions, CH_3_OH) *m*/*z* (%): 304 (100) [CuBr_3_]^−^. Elemental analysis (%) calculated for C_36_H_46_Br_2_CuN_10_O_2_: C 49.46, H 5.30, N 16.02; found: 49.59, H 5.27, N 16.11.

#### 3.1.5. Synthesis of [Cu(L^Ad^)(PPh_3_)]PF_6_ (**4**)

Tetrakis(acetonitrile)copper(I)hexafluorophosphate (1.000 mmol, 0.372 g) and triphenylphosphine (1.000 mmol, 0.260 g) were dissolved in CH_3_CN (30 mL). The reaction was stirred for 3 h at room temperature, and then the ligand L^Ad^ (1.000 mmol, 0.325 g) was added. The reaction was stirred for 24 h at room temperature. The white solution was filtered, and the mother liquor was evaporated at reduced pressure, obtaining a solid that was washed with diethyl ether (10 mL) and n-hexane (10 mL). The solution was filtered, giving complex [Cu(L^Ad^)(PPh_3_)]PF_6_ in 59% yield (0.470 g). Mp: 238–240 °C. Solubility: CH_3_OH, THF, CH_2_Cl_2_, CHCl_3_, ethyl acetate, CH_3_CN, DMSO. FT-IR (cm^−1^): 3382wbr (N-H); 3134wbr, 3055wbr, 2909w, 2853w (C-H); 1670s (C=O); 1542mbr, 1481w (C=C/C=N); 1436m (C=C/C=N); 1403m, 1361w, 1309m, 1289m, 1229w, 1096m, 1056w, 1028w, 998w, 978w, 918w; 834vs (P-F); 745s, 693s, 615s, 601m, 573m, 556vs, 531s, 505s, 492s, 448m. ^1^H-NMR (CD_3_CN, 293K): δ 1.65–2.18 (m, 15H, C*H*_ADM_), 6.38 (t, 2H, 4-C*H*_Pz_), 6.78 (s, 1H, N*H*), 6.98 (s, 1H, C*H*CO), 7.39–7.54 (m, 15H, C*H*_Ar_), 7.60 (d, 2H, C*H*_Pz_), 7.85 (d, 2H, C*H*_Pz_). ^13^C{^1^H}-NMR (CD_3_CN, 293K): 29.3, 35.8, 40.6 (*C*H_ADM_), 74.1 (*C*HCO), 106.8 (4-*C*H_Pz_), 128.9, 129.0, 130.4, 131.5, 132.3, 132.7, 133.3, 133.5 (*C*H_Pz_ and *C*H_Ar_), 141.5 (*C*H_Pz_), 162.0 (*C*=O). ^31^P{^1^H}-NMR (CD_3_CN, 293K): δ −144.62 (sept, J_F-P_ = 706 Hz), −0.81 (sbr). ESI-MS(+) (major positive ions, CH_3_CN) *m*/*z* (%): 587 (60) [Cu(PPh_3_)_2_]^+^, 650 (100) [Cu(L^Ad^)(PPh_3_)]^+^. ESI-MS(-) (major negative ions, CH_3_CN) *m*/*z* (%): 145 (100) [PF_6_]^−^. Elemental analysis (%) calculated for C_36_H_38_CuF_6_N_5_OP_2_: C 54.31, H 4.81, N 8.80; found: C 54.16, H 4.95, N 8.50.

#### 3.1.6. Synthesis of [Cu(L^Ad^)(PPh_3_)_2_]PF_6_ (**5**)

Triphenylphosphine (1.000 mmol, 0.260 g) was added to a solution of tetrakis(acetonitrile)copper(I)hexafluorophosphate (0.500 mmol, 0.186 g) in CH_3_CN (30 mL). The reaction was stirred for 3 h at room temperature, and then the ligand L^Ad^ (0.500 mmol, 0.163 g) was added. The reaction was stirred for 24 h at room temperature. The solvent was evaporated at reduced pressure, giving an oil that was washed with diethyl ether and n-hexane to give complex [Cu(L^Ad^)(PPh_3_)_2_]PF_6_ in 66% yield (0.349 g). Mp: 295–297 °C. Solubility: CH_3_OH, EtOH, diethyl ether, THF, CH_2_Cl_2_, CH_3_CN, DMSO. FT-IR (cm^−1^): 3405sh, 3384wbr (N-H); 3149wbr, 3055wbr, 2912m, 2853w (C-H); 1673m (C=O); 1543wbr, 1482w, 1437w (C=C/C=N); 1406w, 1361w, 1310w, 1289w, 1180wbr, 1119w, 1097m, 1073m, 1053w, 997w, 918w; 837vs (P-F); 769s, 746s, 722s, 695s, 613m, 600m, 556s, 532s, 510m, 485m, 443m. ^1^H-NMR (CDCl_3_, 293K): δ 1.22–2.07 (m, 15H, C*H*_ADM_), 6.38 (s, 2H, 4-C*H*_Pz_), 7.13 (s, 1H, C*H*CO), 7.28–7.51 (m, 33H, C*H*_Ar_, N*H* and C*H*_Pz_), 8.10 (s, 2H, C*H*_Pz_). ^1^H-NMR (CD_3_CN, 293K): δ 1.65–2.04 (m, 15H, C*H*_ADM_), 6.38 (s, 2H, 4-C*H*_Pz_), 6.88 (s, 1H, N*H*), 7.04 (s, 1H, C*H*CO), 7.32–7.51 (m, 30H, C*H*_Ar_), 7.59 (s, 2H, C*H*_Pz_), 7.87 (s, 2H, C*H*_Pz_). ^13^C{^1^H}-NMR (CD_3_CN, 293K): 29.3, 35.8, 40.7 (*C*H_ADM_), 74.7 (*C*HCOO), 106.7 (4-*C*H_Pz_), 128.9, 129.0, 130.9 (*C*H_Ar_), 132.3 (*C*H_Pz_), 132.6, 133.3, 133.4 (*C*H_Ar_), 141.1 (*C*H_Pz_), 162.2 (*C*=O). ^31^P{^1^H}-NMR (CDCl_3_, 293K): δ −143.82 (sept, J_F-P_ = 713 Hz), 1.98 (sbr). ^31^P{^1^H}-NMR (CD_3_CN, 293K): δ −144.63 (sept, J_F-P_ = 706 Hz), −0.47 (sbr). ESI-MS(+) (major positive ions, CH_3_CN) *m*/*z* (%): 145 (80) [Cu + CH_3_CN]^+^, 357 (100) [Cu(PPh_3_)]^+^, 650 (50) [Cu(L^Ad^)(PPh_3_)]^+^. ESI-MS(-) (major negative ions, CH_3_CN) *m*/*z* (%): 145 (100) [PF_6_]^−^. Elemental analysis (%) calculated for C_54_H_53_CuF_6_N_5_OP_3_: C 61.27, H 5.05, N 6.62; found: 60.99, H 4.98, N 6.81.

#### 3.1.7. Synthesis of [Cu(L^Ad^)(PTA)]PF_6_ (**6**)

Tetrakis(acetonitrile)copper(I)hexafluorophosphate (0.500 mmol, 0.186 g) and 1,3,5-triaza-phosphaadamantane (0.500 mmol, 0.157 g) were dissolved in CH_3_CN (30 mL). The reaction was stirred for 3 h at room temperature, and then the ligand L^Ad^ (0.500 mmol, 0.163 g) was added to the solution. The reaction was stirred for 24 h at room temperature. The solvent was evaporated at reduced pressure, giving an oil that was washed with diethyl ether (30 mL) and then filtered, giving complex [Cu(L^Ad^)(PTA)]PF_6_ in 58% yield (0.200 g). Mp: 220–225 °C. Solubility: CH_3_CN, DMSO. FT-IR (cm^−1^): 3393wbr (N-H); 3135wbr, 2908m (C-H); 1701m (C=O); 1522m, 1451m (C=C/C=N); 1403m, 1361w, 1293m, 1241m, 1099m, 1056w, 1016m, 971s, 950m; 834vs (P-F); 761s, 613m, 573vs, 556vs, 450m. ^1^H-NMR (CD_3_CN, 293K): δ 1.69–2.22 (m, 15H, C*H*_ADM_), 4.10 (s, 6H, NC*H*_2_P), 4.49–4.57 (AB q, 6H, NC*H*_2_N), 6.42 (s, 2H, 4-C*H*_Pz_), 6.57 (s, 1H, N*H*), 7.00 (s, 1H, C*H*CO), 7.66 (s, 2H, C*H*_Pz_), 7.88 (s, 2H, C*H*_Pz_). ^13^C{^1^H}-NMR (CDCl_3_, 293K): 29.2, 36.0, 40.5, (*C*H_ADM_), 51.4 (N*C*H_2_P), 69.6 (N*C*H_2_N), 73.5 (*C*HCOO), 107.1 (4-*C*H_Pz_), 132.7 (*C*H_Pz_), 142.3 (*C*H_Pz_), 162.6 (*C*=O). ^31^P{^1^H}-NMR (CD_3_CN, 293K): δ −144.60 (sept, J_F-P_ = 706 Hz), −94.53 (sbr). ESI-MS(+) (major positive ions, CH_3_CN) *m*/*z* (%): 545 (100) [Cu(L^Ad^)(PTA)]^+^. ESI-MS(-) (major negative ions, CH_3_CN) *m*/*z* (%): 145 (100) [PF_6_]^−^. Elemental analysis (%) calculated for C_24_H_35_CuF_6_N_8_OP_2_: C 41.71, H 5.10, N 16.21; found: C 41.42, H 5.27, N 15.97.

#### 3.1.8. Synthesis of [Cu(L^Ad^)(PTA)_2_]PF_6_ (**7**)

To a solution of tetrakis(acetonitrile)copper(I)hexafluorophosphate (0.500 mmol, 0.186 g) in CH_3_CN (30 mL), 1,3,5-triaza-phosphaadamantane (1.100 mmol, 0.173 g) was added. The reaction was stirred for 3 h at room temperature, and successively the ligand L^Ad^ (0.500 mmol, 0.163 g) was added. The reaction was stirred for 24 h at room temperature. The opalescent solution obtained was filtered, and the mother liquors were evaporated, giving complex [Cu(L^Ad^)(PTA)_2_]PF_6_ in 81% yield (0.344 g). Mp: 230–232 °C. Solubility: CH_2_Cl_2_, CH_3_CN, DMSO. FT-IR (cm^−1^): 3318wbr (N-H); 3112wbr, 2906mbr (C-H); 1687mbr (C=O); 1519wbr, 1449m (C=C/C=N); 1413m, 1293m, 1242s, 1102m, 1044m, 1014s, 969s, 947s, 893w; 833vs (P-F); 740s, 649m, 618m, 582vs, 556vs, 463s, 450s. ^1^H-NMR (CD_3_CN, 293K): δ 1.71–2.17 (m, 15H, C*H*_ADM_), 4.08 (s, 12H, NC*H*_2_P), 4.50–4.62 (AB q, 12H, NC*H*_2_N), 6.36 (t, 2H, 4-C*H*_Pz_), 6.87 (s, 1H, N*H*), 6.94 (s, 1H, C*H*CO), 7.59 (d, 2H, C*H*_Pz_), 7.80 (d, 2H, C*H*_Pz_). ^13^C{^1^H}-NMR (CD_3_CN, 293K): 29.4, 35.9, 40.8 (*C*H_Ad_), 52.6 (N*C*H_2_N), 72.5 (N*C*H_2_P), 75.1 (*C*HCOO), 106.8 (4-*C*H_Pz_), 130.6 (*C*H_Pz_), 140.9 (*C*H_Pz_), 162.0 (*C*=O). ^31^P{H}-NMR (CD_3_CN, 293K): δ −144.62 (sept, J_F-P_ = 706 Hz), −89.91 (sbr). ESI-MS(+) (major positive ions, CH_3_CN) *m*/*z* (%), 261 (80) [Cu(PTA) + CH_3_CN]^+^, 545 (100) [Cu(L^Ad^)(PTA)]^+^. ESI-MS(-) (major negative ions, CH_3_CN) *m*/*z* (%): 145 (100) [PF_6_]^−^. Elemental analysis (%) calculated for C_30_H_47_CuF_6_N_11_OP_3_: C 42.48, H 5.58, N 18.16; found: C 42.22, H 5.85, N 18.49.

### 3.2. Spectroscopic Techniques

#### 3.2.1. Synchrotron Radiation-Induced X-Ray Photoelectron Spectroscopy (XPS)

XPS measurements were performed on the five coordination compounds deposited as thick films onto Au/Si(111) wafer substrates using a drop-casting procedure. SR-XPS experiments were performed at the SuperESCA beamline at the ELETTRA synchrotron facility of Trieste (Italy), collecting the data in fixed analyzer transmission mode (pass energy = 20 eV), with the monochromator entrance and exit slits optimized at 30 and 20 μm, respectively. To optimize signals intensity and resolution, a Photon Energy (PE) value of 600 eV was used to collect data at C1s, O1s, and N1s core-levels, PE = 360 eV for C1s, Au4f (substrate reference), P2p, Cl2p, and Br3d spectral regions, and PE = 1100 eV for Cu2p, F1s, and C1s (calibration). The energy resolution was ΔE = 0.25 eV in the entire energy range. Calibration of the energy scale was made referencing the spectra to the C1s core level signal of aliphatic carbons, found at 285.0 eV, for all samples [77]. Curve-fitting analysis of the C1s, O1s, N1s, F1s, P2p, Cl2p, Br3d, and Cu2p spectra was carried out using Gaussian curves as fitting functions, after subtraction of a polynomial background. The Cl2p_3/2,1/2_, P2p_3/2,1/2_, and Cu2p_3/2,1/2_ doublets were fitted using the same full width at half-maximum (FWHM) for both components, a spin−orbit splitting of 1.6, 0.8, and 19.8 eV, respectively, and a branching ratio (2p_3/2_/2p_1/2_) of 2. For the Br3d_5/2,3/2_ doublets, a splitting of 6.6 eV, a branch ratio 3d_5/2_/3d_3/2_ of 3/2 and the same FWHM values for both spin−orbit components were applied. When several different species were identified in a spectrum, the same FWHM value was set for all individual photoemission bands.

#### 3.2.2. Near Edge X-Ray Absorption Fine Structure (NEXAFS) Spectroscopy

NEXAFS experiments were carried out at the BEAR beamline (Bending magnet for Emission Absorption and Reflectivity) at the ELETTRA storage ring, installed at the left exit of the 8.1 bending magnet exit. The apparatus is based on a bending magnet as a source and beamline optics delivering photons from 5 eV up to about 1600 eV; the UHV end station is equipped with a movable hemispherical electron analyzer. In these experiments, we used ammeters to measure drain current from the sample. Investigations were carried out on thick films deposited onto Au/Si(111) substrates (same samples used to collect XPS spectra); C and N K-edge spectra were recorded at grazing (20°) incidence angle of the linearly polarized photon beam with respect to the sample surface, to maximize signal intensity. The raw spectra were normalized to the incident photon flux by dividing the sample spectrum by the spectrum collected on a freshly sputtered gold surface; subsequently a straight line that fits the part of the spectrum below the edge was subtracted. The values at 330.00 and 420.00 eV for C and N, respectively, were assessed to 1.

In order to calibrate the energy scale of the C K edge spectra, the transition of the amide function in the side chain of the L^2Ad^ ligand was used as reference [42]; for the N K edge spectra, the transition of the pyrazole rings [52].

#### 3.2.3. X-Ray Absorption Spectroscopy (XAS)

XAS experiments were carried out at the LISA (BM08) beamline [48] (proposals ESRF IH-MD58 and CERIC-ERIC#20232011) at the European Synchrotron Radiation Facility (ESRF) in Grenoble. The beamline was equipped with a Si (111) double crystal monochromator (DCM), a couple of collimating (before the DCM) and a focusing (after the DCM) mirrors, coated with Si for removal of high order harmonics. Dry powders of copper complexes were mixed with cellulose (50 mg) and gently ground in a mortar to have a homogeneous mixture, which was then pressed into pellets suitable for handling. Cu K edge (8979 eV) was probed at ambient temperature (25 °C) in the vacuum sample holder chamber, in fluorescence geometry. The intensity of the incoming x-ray beam (I_o_) was measured using an N_2(g)_-filled ionization chamber placed before the sample. A 13-element HP-Ge ORTEC detector was used to collect the total x-ray fluorescence signal; the Cu K_α_ fluorescence was selected using the multichannel analyzer electronics. The energy calibration was achieved by measuring a metallic Cu foil as reference material after the sample. Two N_2(g)_-filled ionization chambers (I_1_ and I_2_, between the sample and foil and right after the foil, respectively) were used to measure the beam intensity before and after the reference simultaneously with sample acquisition. The raw absorption signals from samples (fluorescence geometry) and reference foil (transmission geometry) were, respectively, calculated as follows:

(1) $$\alpha_{fluo}=\sum_{i}\frac{I_{fi}}{I0}\;\alpha_{ref}=\ln\left(\frac{I1}{I2}\right)$$

where $\sum_{i}I_{fi}\;$ is the sum over the fluorescence signals of the elements of the detector.

For each sample six scans were acquired, checked for energy scale alignment, cleaned from spikes and glitches, and averaged up to improve signal-to-noise ratio. The raw experimental spectra were treated along the standard procedures for background subtraction (α’ = α_exp_ − α_pre_), edge jump normalization, and bare atom background subtraction (α_b_) [78] to extract the EXAFS structural signals $\chi_{exp}\left(k\right)=\frac{\alpha ’-\alpha_{b}}{\alpha_{b}}$. The edge energy (E_0_) defines the energy scale of the photoelectron wavenumber $k\left[{Å}^{-1}\right]=\hbar^{-1}\sqrt{2m_{e}\left(E-E_{0}\right)}$ (where m_e_ is the mass of the electron, and E and E_0_ in eV), and it was selected as the first inflection point (first maximum of the first derivative) for all spectra. Quantitative analysis of the EXAFS signals was achieved by fitting the k^2^-weighted theoretical curves $k^{2}\chi_{th}$ to the raw experimental data $k^{2}\chi_{exp}$. The theoretical curves χ_th_(k) were calculated as a sum of selected partial contributions χ_i_, obtained by calculation of the photoelectron scattering amplitudes and phase functions using the FEFF8.4 [46] software using atomic mini-clusters based on the complex structures, optimized by DFT calculations (see Section 2.4 above). The χ_i_ were calculated using the standard EXAFS formula with Gaussian disorder approximation [79,80,81], applying not linear least-square procedure implemented in the program FiteEXA [78]. For each sample, the relevant single (SS) and multiple scattering (MS) contributions to the EXAFS signal were identified, and those with similar path length and amplitude were grouped. A trial-and-error procedure was performed with the aim of minimizing the free variables within each sample.

### 3.3. Biological Studies

#### 3.3.1. Cell Lines

U87 MG and LN18 human glioblastoma cell lines were initially obtained from the American Tissue Culture Collection (ATCC, Manassas, VA, USA). All experiments were performed on cells between the 7th and 9th passages. Both cell lines were grown as monolayers in Dulbecco’s Modified Eagle Medium (DMEM) plus HAM’s F12 (in a 1:1 ratio), supplemented with 10% heat-inactivated fetal bovine serum (FBS), L-glutamine (2 mM), penicillin (100 IU/mL), and streptomycin (100 µg/mL), and maintained at 37 °C in a 5% CO_2_ humidified atmosphere. Upon reaching confluency (>85%), for passaging, cells were detached with a 0.05% trypsin and 0.002% EDTA solution. All media and supplements for cell cultures were acquired from Gibco (Thermo Fisher Scientific, Inc., Waltham, MA, USA).

#### 3.3.2. Cell Viability

The effects of copper complexes and cisplatin on cell viability were analyzed in GBM cell lines using the 3-[4,5-dimethylthiazole-2-yl]-2,5-dimethyltetrazolium bromide (MTT) assay. U87 MG and LN18 cells were seeded in 96-well plates (at a density of 1.5 × 10^4^ and 1.8 × 10^4^ cells/well, respectively). Copper complexes and cisplatin were dissolved in DMSO to obtain 10 mM stock solutions and diluted in culture medium to obtain the maximum DMSO concentration of 0.5% (*v*/*v*) in cell cultures. After 24 h of adhesion, each cell line was treated with L^Ad^, cisplatin, and complexes **1**–**7** (concentrations ranging from 1 to 50 µM) for 24, 48, and 72 h. Cells were then incubated with a 0.5 mg/mL solution of MTT (Sigma–Aldrich) for 3 h at 37 °C. Then, supernatants were removed, and formazan was solubilized with 100 μL DMSO. The absorbance was read at 570 nm using a Varioskan™ LUX multimode plate spectrophotometer (Thermo Fischer Scientific). Results are expressed as mean ± standard deviation (SD) of three independent experiments performed in triplicate. The IC_50_ inhibitory concentrations for tested compounds was calculated from the nonlinear regression line using GraphPad Prism software (version 10, San Diego, CA, USA).

#### 3.3.3. Intracellular Reactive Oxygen Species (ROS) Level Evaluation

Intracellular ROS levels were measured by DCFDA assay. DCFDA (carboxy-2′,7′-dichlorofluorescein diacetate or H_2_DCFDA) is generally used as a cell permeable probe to detect intracellular ROS species and determine the overall oxidative stress degree. This probe, upon cleavage by intracellular esterases, produces the non-fluorescent H_2_DCF which is then oxidized by ROS to produce the highly fluorescent 2’,7’-dichlorofluorescein (DCF). U87 MG (2 × 10^5^ cells/well) and LN18 cells (2.5 × 10^5^ cells/well) were seeded in 6-well plates. After 24 h, the IC_50_ concentrations of the Cu complexes (in DMSO for control samples) were added to the medium. After 24-, 48-, and 72-h incubation times, Cu compound-containing media were removed; cells were washed with PBS and pulsed with DCFDA (2.5 µM) for 10 min at 37 °C. To use H_2_O_2_ as a positive control for ROS measurements, a concentrated stock (100 mM) from 30% H_2_O_2_ was prepared by dilution in culture medium. Then, glioblastoma cells were treated with H_2_O_2_ (final concentration 100 µM) for 1 h and pulsed with DCFDA as above. Samples were then detached with EDTA and trypsin, resuspended in ice-cold PBS, and immediately analyzed by an LSRII flow cytometer (Becton, Dickinson and Company, Franklin Lakes, New Jersey, USA). Fluorescence emission was collected through a 530 nm band-pass filter to analyze DCFDA signal. At least 10,000 cells per sample were acquired in log mode. The MFI values were calculated through the FACS Diva software (Becton, v5.0.3). Data are expressed as fold obtained by the ratio of MFI values from treated cells and those from control ones. Values (means ± SD) represent the average from three independent experiments.

#### 3.3.4. Intracellular Glutathione (GSH) Level Evaluation

To evaluate intracellular GSH levels, U87 MG (2 × 10^5^ cells/well) and LN18 (2.4 × 10^4^ cells/well) cells were seeded in 6-well plates. Cells were then treated with compounds **4** (5 and 10 μM for U87 MG; 4 and 8 μM for LN18) and **5** (2.5 and 5 μM for U87 MG; 1.75 and 3.5 μM for LN18) for 4, 24, 48, and 72 h. To use H_2_O_2_ as a positive control for GSH measurements, a concentrated stock (100 mM) from 30% H_2_O_2_ was prepared by dilution in culture medium. Then, glioblastoma cells were treated with 100 µM H_2_O_2_ for 1 h. After treatment, cells were incubated with monochlorobimane (MCB, Molecular Probes) at a final concentration of 25 µg/mL. Staining was performed at 37 °C for 10 min prior to flow cytometry analysis. Fluorescence intensity, indicative of intracellular GSH content, was measured using a CytoFLEX LX flow cytometer (Beckman Coulter, Brea, CA, USA) equipped with a UV laser (355 nm). MCB fluorescence was detected in the UV525-A channel. Data were acquired using CytExpert v2.4 software, and the MFI was calculated from the major cell population peak. At least 10,000 viable cells were acquired per sample. Results are reported as mean ± SD from three independent experiments. The gating strategy for intracellular GSH analysis is shown in Supporting Information Figure S29. Representative fluorescence histograms of GSH levels at 24 and 48 h are provided in Supporting Information Figure S34.

#### 3.3.5. Cell Cycle Analysis

The cell cycle distribution in U87 MG and LN18 glioma cell lines was assessed following treatment with Cu compounds **4** and **5**. U87 MG (2 × 10^5^ cells/well) and LN18 (2.5 × 10^5^ cells/well) cells were seeded in 6-well plates. After 24 h of adhesion, the cells were treated with compounds **4** (5 and 10 μM for U87 MG; 4 and 8 μM for LN18) and **5** (2.5 and 5 μM for U87 MG; 1.75 and 3.5 μM for LN18). Following 24, 48, and 72 h of incubation, both untreated and treated cells were collected, washed with cold PBS, and fixed in 70% ethanol for 1 h at 4 °C. After fixation, the cells were washed again with cold PBS and stained with 40 μg/mL PI and 100 μg/mL RNase in PBS for 1 h at 37 °C. DNA content was analyzed using a Gallios flow cytometer (Beckman Coulter), and cell cycle distribution (G0/G1, S, and G2/M phases) was determined using Kaluza Analysis 2.4 software (Beckman Coulter). At least 10,000 viable cells were acquired per sample. The gating strategy for cell cycle analysis is shown in Supporting Information Figure S29. Representative histograms of cell cycle profiles at 48 h are shown in Supporting Information Figure S30, while additional histograms at 72 h are reported in Supporting Information Figure S35.

#### 3.3.6. Apoptosis Assessment

Apoptosis evaluation was performed using a fluorescein isothiocyanate (FITC)-conjugated annexin V (AV) and propidium iodide (PI) detection kit according to the manufacturer’s protocol (Annexin V-FITC Apoptosis Detection Kit, AB14085, Abcam, Cambridge, UK). U87 MG and LN18 cell lines were seeded in 6-well plates, as described above. After 24 h of adhesion, the cells were treated with compounds **4** (5 and 10 μM for U87 MG; 4 and 8 μM for LN18) and **5** (2.5 and 5 μM for U87 MG; 1.75 and 3.75 μM for LN18). Following 24, 48, and 72 h of incubation, both untreated and treated cells were collected, washed with PBS, and resuspended in 500 μL of binding buffer containing 5 μL of an annexin V-FITC solution and an equal volume of PI solution. Subsequently, cells were then incubated for 5 min at room temperature in the dark and analyzed using a Gallios flow cytometer (Beckman Coulter). Data were analyzed using Kaluza Analysis 2.4 software, allowing discrimination between viable, early apoptotic, late apoptotic, and necrotic cells. At least 10,000 viable cells were acquired per sample. Results are reported as mean ± SD from three independent experiments. The gating strategy for apoptosis analysis is shown in Supporting Information Figure S29. Representative Annexin V/PI dot plots at 48 h are provided in Supporting Information Figure S30.

#### 3.3.7. Western Blot (WB) Analysis

The cells were lysed in RIPA lysis buffer containing 50 mM Tris/HCl (pH 8.0), 150 mM NaCl, 1% NP40 nonidet, 0.5% sodium deoxycholate, 0.1% SDS, 1 mM PMSF, 2 mM Na_3_VO_4_, 20 mM NaF, and 1% protease inhibitor cocktail (Sigma–Aldrich). Lysates were clarified by centrifugation, and the protein content was determined using the Bradford reagent (Bio-Rad, Segrate, Italy). A total of 40 µg of each cell extract was separated on a 10–14% gel by SDS-PAGE, transferred to a nitrocellulose membrane (Bio-Rad), blocked with 5% *w*/*v* non-fat milk (Bio-Rad) in Tris-buffered saline with Tween (TBS-T; 20 mM Tris, 150 mM NaCl (pH 7.6), and 0.1% Tween-20) and probed with primary antibodies against cleaved caspase-3, cleaved caspase-6, cleaved caspase-7, cleaved PARP and Bcl-xl (1:1000, Cell Signaling Technology, Milan, Italy) at 4 °C with gentle shaking, overnight. The primary antibodies were detected using peroxidase-conjugated anti-rabbit secondary antibody (1:2000, Cell Signaling Technology). The ChemiDoc XRS+ imager (Bio-Rad) was used for membrane exposure and image acquisition. The densities of the bands were quantified using the Image LabTM Software version 6.1.0 (Bio-Rad). The values, normalized to GAPDH, are reported as fold change vs. untreated control. The results were from at least three independent experiments presented as the mean ± SD.

#### 3.3.8. Phase Contrast Microscopy

Cells were seeded in six-well plates (2 × 10^5^ cells/well for U87 MG and 2.5 × 10^5^ cells/well for LN18) and incubated for 24 h to allow for initial adhesion. Cells were then treated with compounds **4** and **5** for 24 h. Images were acquired using an inverted phase contrast microscope equipped with an Olympus digital camera.

#### 3.3.9. Immunofluorescence Microscopy

U87 MG and LN18 cells were seeded on 12 mm diameter coverslips in 24-well plates at a density of 5.5 × 10^4^ cells/well. After incubation for 24 h at 37 °C in a humidified 5% CO_2_ atmosphere cultures were treated with compounds **4** and **5** for an additional 24 h. Cells were subsequently fixed with 4% paraformaldehyde for 30 min and permeabilized with 0.5% Triton X-100 (Sigma–Aldrich) for 5 min. For actin detection, cells were incubated with FITC-conjugated phalloidin (Sigma–Aldrich) for 30 min at room temperature. Nuclear staining was performed using Hoechst 33,258 (Sigma–Aldrich, #861405) at 37 °C for 15 min. After washing with PBS, coverslips were mounted using a glycerol–phosphate mounting medium. Fluorescence images were acquired using a Nikon Eclipse Ti2 microscope (Amstelveen, The Netherlands) equipped with a 60× objective.

#### 3.3.10. Laser Scanning Confocal Microscopy Analysis

To investigate the mitochondrial and pro-apoptotic effects of compound **5**, LN18 glioblastoma cells were cultured on 12 mm glass coverslips in 24-well plates at a density of 5.5 × 10^4^ cells/well and incubated for 24 h at 37 °C in a humidified 5% CO_2_ atmosphere. Cells were then treated with compound **5** at sub-cytotoxic concentration (0.9 µM) for 6 h to assess early mitochondrial and apoptotic changes. After treatment, cells were incubated with MitoTracker^®^ Red CMXRos (1:1000 dilution from 1 mM stock) for 30 min at 37 °C. Cells were then washed with PBS, fixed with 3.7% paraformaldehyde for 15 min at room temperature, permeabilized with 0.5% Triton X-100 for 10 min, and blocked for 30 min with PBS containing 1% BSA and 10% FBS. Next, cells were incubated for 30 min at room temperature with primary antibodies targeting BAX (6A7), BCL2 (Santa Cruz Biotechnology, Dallas, TX, USA), or cytochrome c (Becton Dickinson), all diluted 1:100 in PBS with 1% BSA. After washing, cells were incubated for 30 min at 37 °C with Alexa Fluor^®^ 488-conjugated anti-mouse IgG secondary antibodies (1:100) and Hoechst 33,342 nuclear stain (1:1000). Finally, coverslips were mounted onto microscope slides using a 1:1 PBS: glycerol solution, and fluorescence imaging was performed using a Leica Stellaris Plus laser scanning confocal microscope (Leica Microsystems, Wetzlar, Germany), with appropriate excitation/emission settings for MitoTracker Red, Alexa Fluor 488, and Hoechst.

#### 3.3.11. Scanning Electron Microscopy Analysis

Scanning electron microscopy (SEM) was employed to analyze the ultrastructural features and alterations of glioblastoma cell lines following Cu compound treatments. U87 MG and LN18 cells were seeded on 12 mm glass coverslips in 24-well plates (65 × 10^3^ and 75 × 10^3^ cells/well, respectively). After 24 h of adhesion, cells were treated with IC_50_ concentrations of compounds that showed the most promising results from the MTT assay (compound **4**: 10 μM for U87 MG; 8 μM for LN18; compound **5**: 5 μM for U87 MG; 3.5 μM for LN18). After 24, 48, and 72 h, cells were fixed in 2.5% glutaraldehyde in 0.2 M Na-cacodylate buffer (pH 7.4) for 2 h at room temperature. Following three washes with the same buffer, cells were fixed with 1% (*w*/*w*) OsO_4_ for 1 h, dehydrated through an ethanol gradient, and treated to dry using hexamethyldisilazane (HDMS, Sigma–Aldrich). Samples were then coated with gold using a sputter coater and analyzed using the FEI Quanta Inspect FeG scanning electron microscope (FEI Company, Inc., Waltham, MA, USA).

### 3.4. Statistical Analysis

Statistical analysis was performed using the two-tailed Student’s *t*-test and one-way ANOVA with Tukey’s multiple comparison test, with GraphPad Prism 10 and Microsoft Excel. Data represents the mean ± SD of at least three independent experiments. All experiments were performed with at least three technical replicates per independent experiment. A *p*-value < 0.05 was considered statistically significant (* *p* < 0.05, ** *p* < 0.01, *** *p* < 0.001, **** *p* < 0.0001).

## 4. Conclusions

In this study, we reported the synthesis, structural characterization, and biological evaluation of a new series of Cu^I^ and Cu^II^ complexes supported by the amantadine-functionalized bis(pyrazol-1-yl)acetate ligand L^Ad^, with the aim of assessing their potential as anticancer agents against GBM. Spectroscopic investigations confirmed the successful coordination of the ligands to copper centers in both oxidation states, and the formation of well-defined molecular architectures featuring distinct copper coordination geometries, as evidenced by SR-XPS and XANES/EXAFS analyses.

Among all synthesized species, the Cu^I^ complexes **4** and **5**, bearing lipophilic triphenylphosphine co-ligands, showed the most potent cytotoxic activity toward U87 MG and LN18 GBM cell lines, with IC_50_ values lower than those of cisplatin. In contrast, Cu^II^ complexes and the Cu^I^ species containing hydrophilic PTA co-ligands were inactive under the tested conditions, highlighting the importance of ligand lipophilicity and metal-centered environment in driving cellular uptake and biological response.

Biological assays demonstrated that compounds **4** and **5** triggered a marked redox imbalance, characterized by increased ROS production and GSH depletion, ultimately resulting in G2/M cell-cycle arrest and cell death. Mechanistic studies revealed cell-line-dependent pathways. Indeed, in LN18 cells, both compounds activated caspase-dependent intrinsic apoptosis, as indicated by caspase-3 and PARP cleavage, Bcl-xL downregulation, Bax mitochondrial translocation, and cytochrome c release.

Taken together, our findings identified lipophilic Cu^I^ complexes incorporating the amantadine-based ligand L^Ad^ as promising anticancer candidates capable of exploiting redox imbalance and mitochondrial vulnerability in GBM cells. Further studies will be carried out to clarify the molecular mechanisms underlying the differential responses of GBM cell lines to the treatments and to strengthen the notion that copper complexes may represent a promising therapeutic strategy for selectively targeting TMZ-resistant GBM cells.

## Figures and Tables

**Table 1:** EXAFS data refinement, best fit results for complexes **1**–**7**. S02 was fixed to 0.9 for all the samples, while ΔE0 (energy scale shift) was fixed to 8 eV and 11 eV for complexes **1**–**3** and **4**–**7**, respectively. Each scattering path is reported in terms of the type of neighbor (single or multiple scattering) N, multiplicity M, average distance R, and mean square displacement σ2. Multiplicities were fixed to the theoretical value for each coordination shell.

Compd.		Shell 1				Shell 2				Shell 3				Shell 4			
	Goodness of Fit	N	M	R [Å]	σ^2^ ×10^−2^	N	M	R [Å]	σ^2^ ×10^−2^	N	M	R [Å]	σ^2^ ×10^−2^	N	M	R [Å]	σ^2^ ×10^−2^
**1**	1.7 × 10^−2^	N	2	2.02(1)	0.27(1)	Cl	2	2.14(1)	2.3(3)	N	2	2.99(3)	0.79(3)	N_1_-C_2_	4	4.33(3)	1.2(5)
**2**	2.7 × 10^−2^	N	2	2.02(1)	0.55(1)	Br	2	2.38(1)	0.65(2)	N	2	3.02(3)	0.58(4)	N_1_-C_2_	4	4.35(4)	0.92(5)
**3**	1.3 × 10^−2^	N	4	2.00(1)	0.83(1)	O	2	2.43(1)	0.52(2)	N	4	2.97(2)	0.97(3)	N_1_-C_5_-N_1_	8	4.27(3)	0.76(6)
**4**	4.3 × 10^−2^	N	2	2.04(1)	1.03(3)	P	1	2.15(1)	0.39(1)	N	2	2.95(3)	1.55(4)	P-C_3_	6	3.53(4)	1.68(7)
**5**	2.2 × 10^−2^	N	2	2.00(1)	0.19(1)	P	2	2.16(2)	2.52(4)	N	2	2.91(3)	0.74(5)	N_1_-C_2_	4	4.35(6)	0.3(1)
**6**	2.3 × 10^−2^	N	2	1.98(2)	0.93(2)	P	1	2.25(1)	0.26(1)	N	2	3.01(2)	1.3(3)	-	-	-	-
**7**	1.8 × 10^−2^	N	2	2.04(1)	0.60(1)	P	2	2.27(1)	0.71(1)	N	2	3.01(3)	1.6(1)	-	-	-	-

**Table 2:** IC_50_ values of L^Ad^, Cu compounds **1**–**7** and cisplatin in U87 MG and LN18 GBM cell lines. MTT assay was used to evaluate cell viability after 72 h of treatment with seven increasing concentrations of each compound (0–50 µM). Values represent the mean ± SD from three independent experiments.

Compounds	IC_50_ (µM) ± SD	
	U87 MG Cell Line	LN18 Cell Line
L^Ad^	>50.0	>50.0
[Cu(L^Ad^)Cl_2_] (**1**)	>50.0	>50.0
[Cu(L^Ad^)Br_2_] (**2**)	>50.0	>50.0
[Cu(L^Ad^)_2_Br_2_] (**3**)	>50.0	>50.0
[Cu(L^Ad^)(PPh_3_)]PF_6_ (**4**)	10.0 ± 1.0	8.0 ± 0.8
[Cu(L^Ad^)(PPh_3_)_2_]PF_6_ (**5**)	5.0 ± 0.5	3.5 ± 0.2
[Cu(L^Ad^)(PTA)]PF_6_ (**6**)	>50.0	>50.0
[Cu(L^Ad^)(PTA)_2_]PF_6_ (**7**)	>50.0	>50.0
Cisplatin	>50.0	15.0 ± 1.8

## Data Availability

The data presented in this study are available from the authors on request.

## Supplementary Materials

The supporting information can be downloaded at https://www.mdpi.com/article/10.3390/ijms27031531/s1.
