# Supplementary material for: Synthesis, Structural Studies, and Biological Evaluation of Copper(I) and Copper(II) Complexes Supported by Bis(pyrazol-1-yl)acetate Ligand Functionalized with Amantadine for the Treatment of Glioblastoma
Source: Int J Mol Sci. 2026 Feb 4;27(3):1531. doi: 10.3390/ijms27031531 (PMC12898142; doi:10.3390/ijms27031531)
Supplement: Supplementary file 1 [file ijms-27-01531-s001.zip › ijms-4060848-supplementary.pdf]

Supporting Information

Synthesis, Structural Studies, and Biological Evaluation of Copper(I) and Copper(II) Complexes Supported by Bis(pyrazol-1-yl)acetate Ligand Functionalized with Amantadine for the Treatment of Glioblastoma

Sofia Migani [1,2], Giuseppina Bozzuto [2], Annarica Calcabrini [2], Marisa Colone [2], Maria Luisa Dupuis [2], Miriam Caviglia [1,2], Cristina Aguzzi [3], Maria Beatrice Morelli [3], Fabio Del Bello [4], Wilma Quaglia [4], Maura Pellei [1], Carlo Santini [1,*], Chiara Battocchio [5], Giovanna Iucci [5], Iole Venditti [5], Carlo Meneghini [5], Simone Amatori [5] and Annarita Stringaro [2,*]

[1] School of Science and Technology, Chemistry Division, University of Camerino, via Madonna delle Carceri (ChIP), Camerino, 62032, Italy; sofia.migani@unicam.it (S.M.); miriam.caviglia@unicam.it (M.C.); maura.pellei@unicam.it (M.P.)
[2] National Center for Drug Research and Evaluation, Italian National Institute of Health, Viale Regina Elena 299, Roma, 00161, Italy; giuseppina.bozzuto@iss.it (G.B.); annarica.calcabrini@iss.it (A.C.); marisa.colone@iss.it (M.C.); marialuisa.dupuis@iss.it (M.L.D.)
[3] School of Pharmacy, Immunopathology and Molecular Medicine Unit, University of Camerino, via Madonna delle Carceri 9, Camerino, 62032, Italy; cristina.aguzzi@unicam.it (C.A.); mariabeatrice.morelli@unicam.it (M.B.M.)
[4] School of Pharmacy, Medicinal Chemistry Unit, University of Camerino, via Madonna delle Carceri (ChIP), Camerino, 62032, Italy; fabio.delbello@unicam.it (F.D.B.); wilma.quaglia@unicam.it (W.Q.)
[5] Department of Science, Roma Tre University, Via della Vasca Navale 79, Roma, 00146, Italy; chiara.battocchio@uniroma3.it (C.B.); giovanna.iucci@uniroma3.it (G.I.); iole.venditti@uniroma3.it (I.V.); carlo.meneghini@uniroma3.it (C.M.); simone.amatori@uniroma3.it (S.A.)
* Correspondence: carlo.santini@unicam.it (C.S.); annarita.stringaro@iss.it (A.S.)

Table of Contents:

Figure S1. FT-IR spectrum of $[Cu(L^{Ad})Cl_2]$ (**1**).

Figure S2. Far-Infrared FT-IR spectrum of $[Cu(L^{Ad})Cl_2]$ (**1**).

Figure S3. FT-IR spectrum of $[Cu(L^{Ad})Br_2]$ (**2**).

Figure S4. Far-Infrared FT-IR spectrum of $[Cu(L^{Ad})Br_2]$ (**2**).

Figure S5. FT-IR spectrum of $[Cu(L^{Ad})_2Br_2]$ (**3**).

Figure S6. Far-Infrared FT-IR spectrum of $[Cu(L^{Ad})_2Br_2]$ (**3**).

Figure S7. FT-IR spectrum of $[Cu(L^{Ad})(PPh_3)]PF_6$ (**4**).

Figure S8. ^{1}H-NMR spectrum of $[Cu(L^{Ad})(PPh_3)]PF_6$ (**4**) in CD_3CN.

Figure S9. ^{13}C{^{1}H}-NMR spectrum of $[Cu(L^{Ad})(PPh_3)]PF_6$ (**4**) in CD_3CN.

Figure S10. ^{31}P{^{1}H}-NMR spectrum of $[Cu(L^{Ad})(PPh_3)]PF_6$ (**4**) in CD_3CN.

Figure S11. FT-IR spectrum of $[Cu(L^{Ad})(PPh_3)_2]PF_6$ (**5**).

Figure S12. ^{1}H-NMR spectrum of $[Cu(L^{Ad})(PPh_3)_2]PF_6$ in CD_3CN (**5**).

Figure S13. $^{13}C\{^1H\}$-NMR spectrum of $[Cu(L^{Ad})(PPh_3)_2]PF_6$ (**5**) in CD_3CN.

Figure S14. $^{31}P\{^1H\}$-NMR spectrum of $[Cu(L^{Ad})(PPh_3)_2]PF_6$ (**5**) in CD_3CN.

Figure S15. FT-IR spectrum of $[Cu(L^{Ad})(PTA)]PF_6$ (**6**).

Figure S16. ^{1}H-NMR spectrum of $[Cu(L^{Ad})(PTA)]PF_6$ (**6**) in CD_3CN.

Figure S17. $^{13}C\{^1H\}$-NMR spectrum of in $[Cu(L^{Ad})(PTA)]PF_6$ (**6**) in $CDCl_3$.

Figure S18. $^{31}P\{^1H\}$-NMR spectrum of $[Cu(L^{Ad})(PTA)]PF_6$ (**6**) in CD_3CN.

Figure S19. FT-IR spectrum of $[Cu(L^{Ad})(PTA)_2]PF_6$ (**7**).

Figure S20. ^{1}H-NMR spectrum of $[Cu(L^{Ad})(PTA)_2]PF_6$ (**7**) in CD_3CN.

Figure S21. $^{13}C\{^1H\}$-NMR spectrum of in $[Cu(L^{Ad})(PTA)_2]PF_6$ (**7**) in CD_3CN.

Figure S22. $^{31}P\{^1H\}$-NMR spectrum of $[Cu(L^{Ad})(PTA)_2]PF_6$ (**7**) in CD_3CN.

Figure S23. C1s (left) and N1s (right) spectra collected on the Cu(II) coordination compounds **1**, **2** and **3** (from top to bottom).

Figure S24. Cl2p or Br3d (left) and Cu2p (right) spectra collected on the Cu(II) coordination compounds **1**, **2** and **3** (from top to bottom).

Figure S25. C1s (left) and N1s (right) spectra collected on the Cu(I) coordination compounds **4**, **5**, **6** and **7** (from top to bottom).

Figure S26. P2p (left), F1s (middle) and Cu2p (right) spectra collected on the Cu(I) coordination compounds **4**, **5**, **6** and **7** (from top to bottom).

Figure S27. Stability studies. 2 mmol of L^{Ad} and complexes **1**-**7** were dissolved in 25 mL of DMSO. UV-Visible spectra were recorded at t = 0 min, t = 24 h, t = 48 h and t = 72 h.

Figure S28. Effects of Cu compounds **1, 2, 3**, **6**, and **7** on cell viability evaluated by MTT assay. Histograms show the viability of GBM cell lines after treatment at increasing concentrations (up to 50 μM) for 24, 48, and 72 hours. Data represent the % of cell viability with respect to the control (set at 100% value). Results are expressed as mean ± SD values from three independent experiments. *$p < 0.05$, **$p < 0.01$, ***$p < 0.001$ (two-tailed Student's t-test).

Figure S29. Gating strategy of the cell population for cell cycle, apoptosis, and intracellular GSH level evaluation study in cytofluorimetry. Cells were first gated on forward scattering area (FSC-A) versus side scattering area (SSC-A) signals to exclude debris (Panel A). FSC-A versus FSC height (Panel B) and FSC width versus SSC height (Panel C) gating strategies were used to exclude doublet events and cell clumps. In addition, an FSC versus V450-PBH dot plot (Panel C) was used to identify and exclude non-viable cells stained with Sytox Green.

In Panel D, an FL-2 area versus FL-2 height gate dot plot was used to define the total cell population in the different phases of the cell cycle.

Figure S30. Effects of the Cu compounds **4** and **5** on the cell cycle distribution and apoptosis after 48 hours. Representative histograms and dot plots show cell cycle profiles and apoptosis analysis in U87 MG and LN18 glioblastoma cell lines after 48 hours of treatment with compounds **4** and **5**.

Figure S31. Western blot analysis of key proteins involved in apoptotic signaling and its regulation in U87 treated cells. Analysis of cleaved caspase-3 and cleaved PARP-1 protein expression in U87 MG cells after 24 and 48 h of treatment with compounds **4** and **5**. GAPDH was used as a loading control.

Figure S32. Phase-contrast microscopy of U87 MG and LN18 cells. Control cells maintained their normal morphology and confluency, whereas treatment with compounds **4** and **5** at their respective IC_{50} doses for 24 hours resulted in cell rounding, detachment, and reduced cell density. Scale bar: 50 μm

Figure S33. Immunofluorescence staining of actin and nuclei in U87 MG and LN18 cells. After 24 h of treatment with compounds **4** and **5** at their respective IC_{50} doses, actin filaments (phalloidin-FITC, green) and nuclei (Hoechst, blue) show cytoskeletal disruption and nuclear condensation in treated cells. Scale bar: 20 μm.

Figure S34. Effects of the compounds **4** and **5** on intracellular GSH levels after 24 and 48 hours. Representative histograms of intracellular GSH levels in U87 MG and LN18 glioblastoma cell lines following 24 and 48 hours of treatment with compounds **4** and **5**. Mean fluorescence intensity (MFI) values are normalized to the untreated control (set to 1). GSH levels were measured by MCB staining.

Figure S35. Effects of compounds **4** and **5** on the cell cycle distribution after 72 hours. Representative histograms showing cell cycle profiles of U87 MG and LN18 glioblastoma cell lines after 72 hours of treatment with compounds **4** and **5**.

Table S1. XPS data analysis results (BE, FWHM, experimental and calculated atomic ratio values and proposed assignments) for samples **1**-**7**.

Table S2. Optimized geometries for complexes **1**-**7** in xyz format.

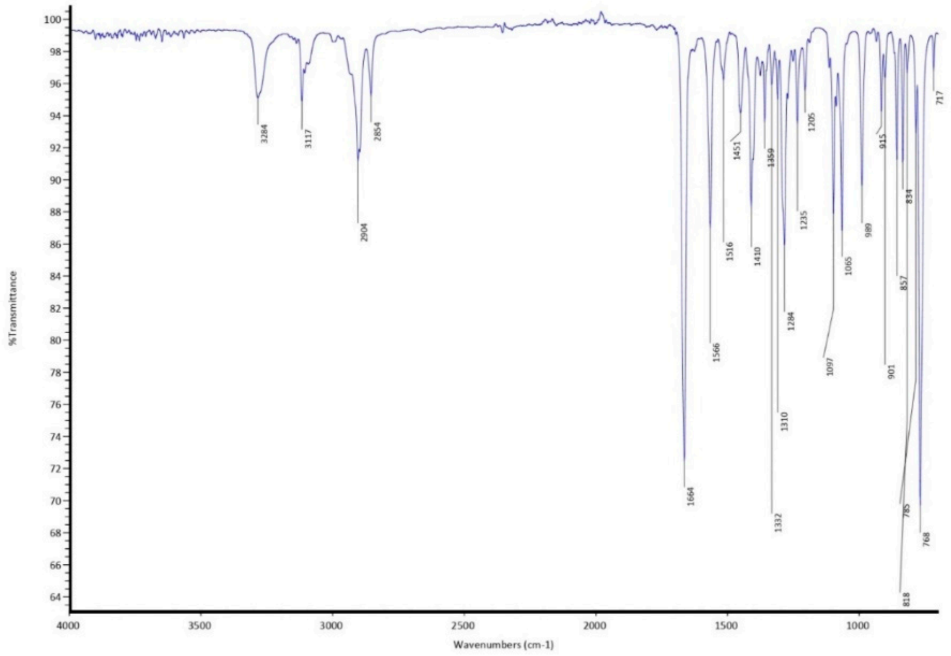

Figure S1. FT-IR spectrum of $[Cu(L^{Ad})Cl_2]$ (**1**).

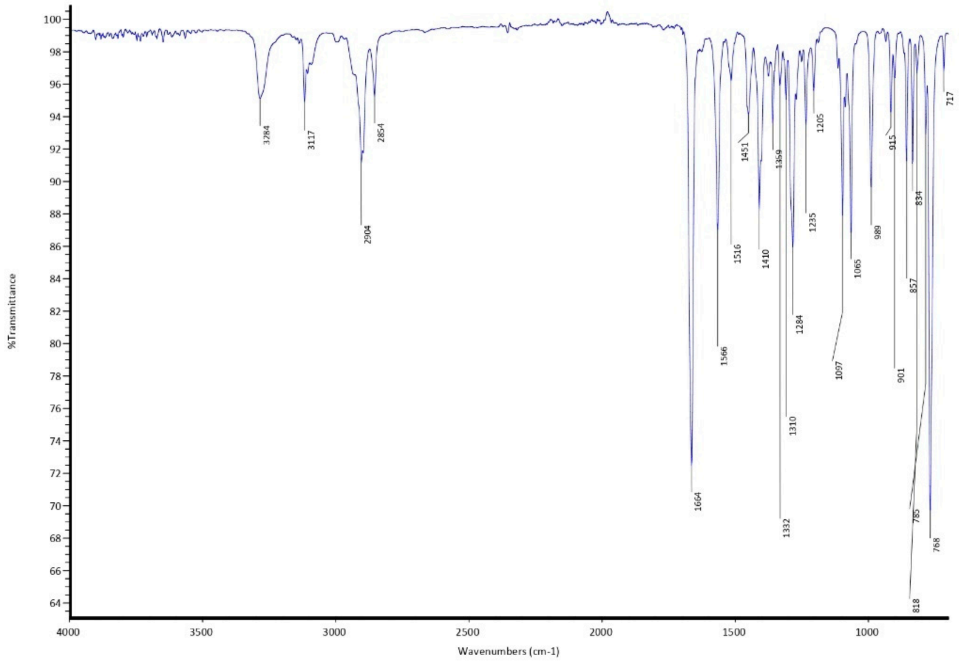

Figure S2. Far-Infrared FT-IR spectrum of $[Cu(L^{Ad})Cl_2]$ (**1**).

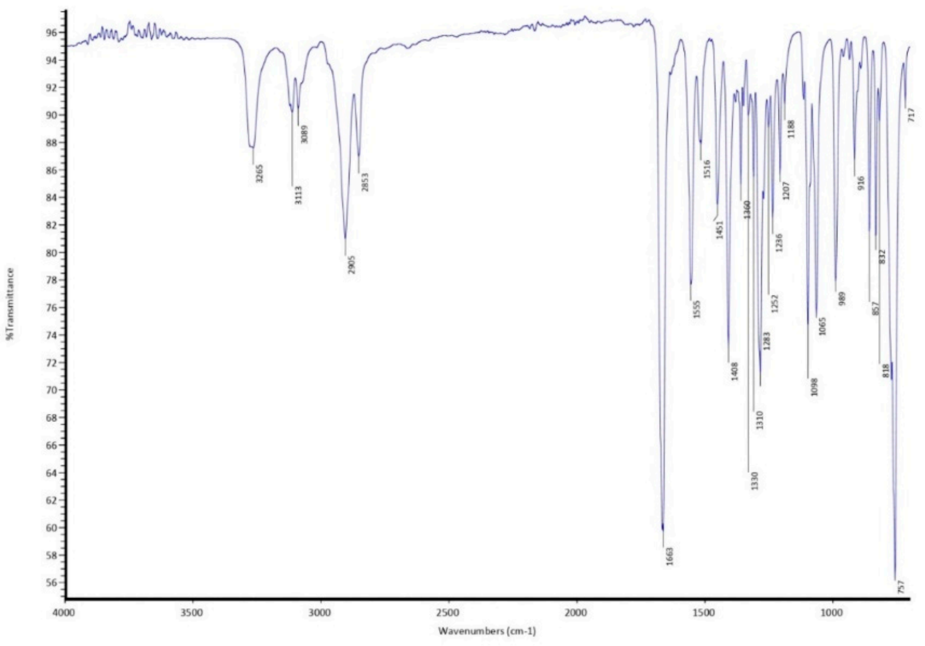

Figure S3. FT-IR spectrum of [Cu(L^{Ad})Br$_2$] (**2**).

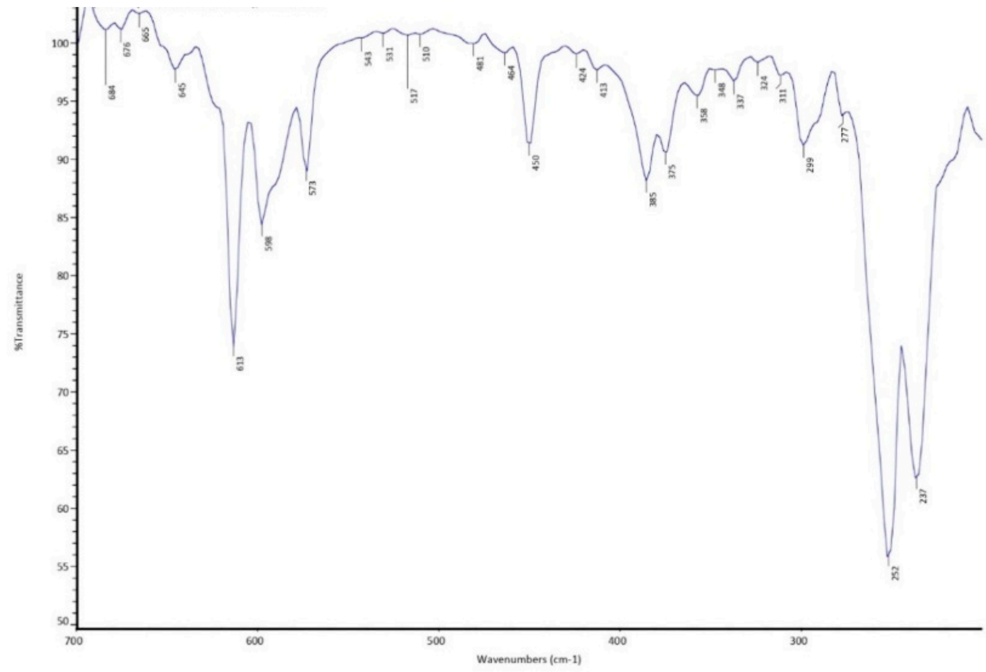

Figure S4. Far-Infrared FT-IR spectrum of [Cu(L^{Ad})Br$_2$] (**2**).

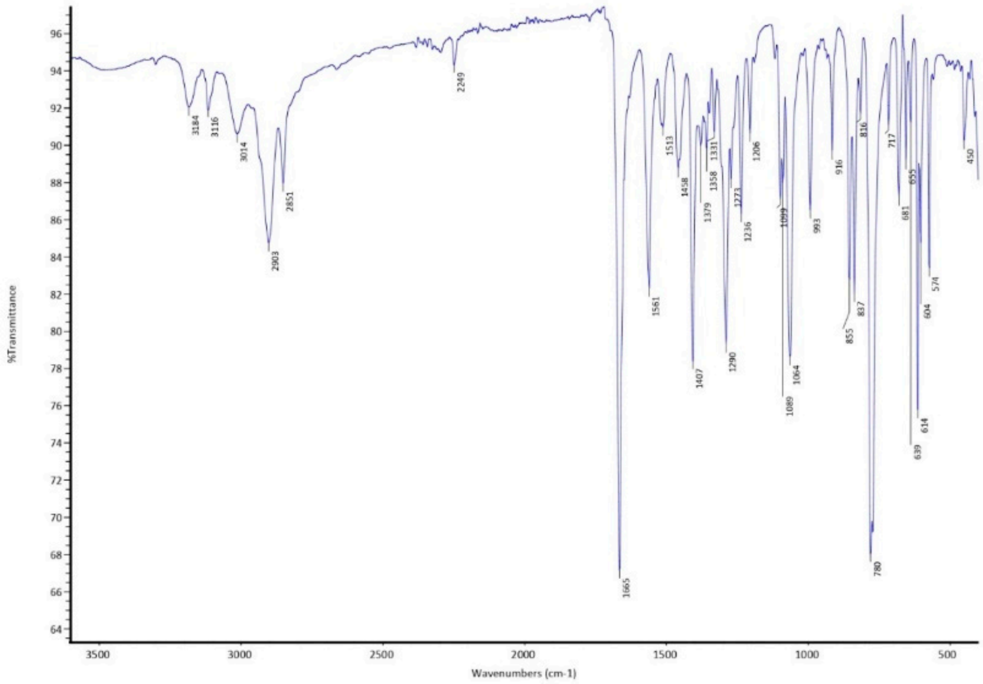

Figure S5. FT-IR spectrum of [Cu(L^{Ad})$_2$Br$_2$] (**3**).

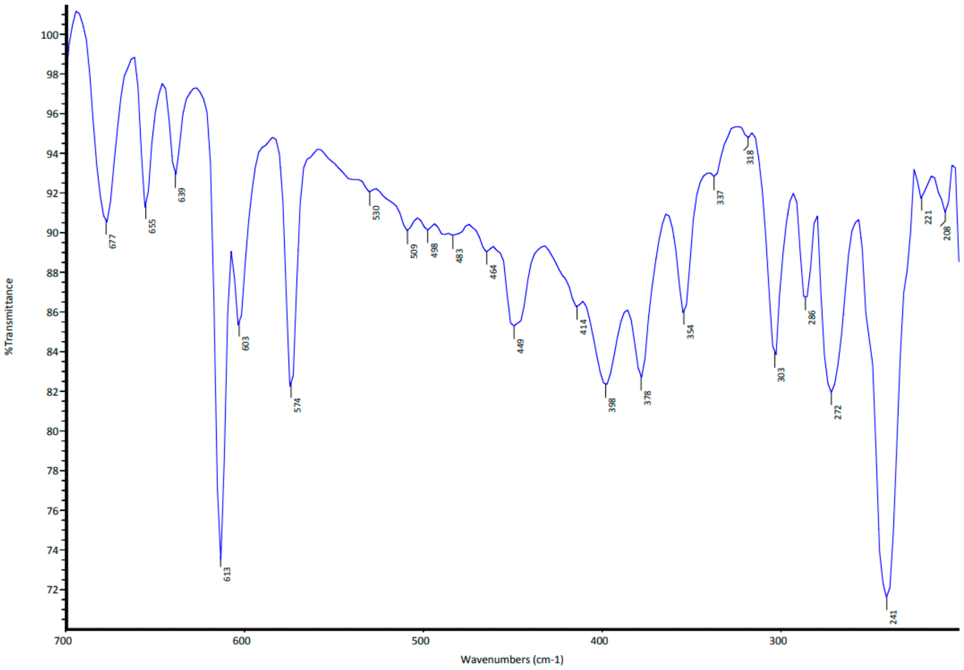

Figure S6. Far-Infrared FT-IR spectrum of [Cu(L^{Ad})$_2$Br$_2$] (**3**).

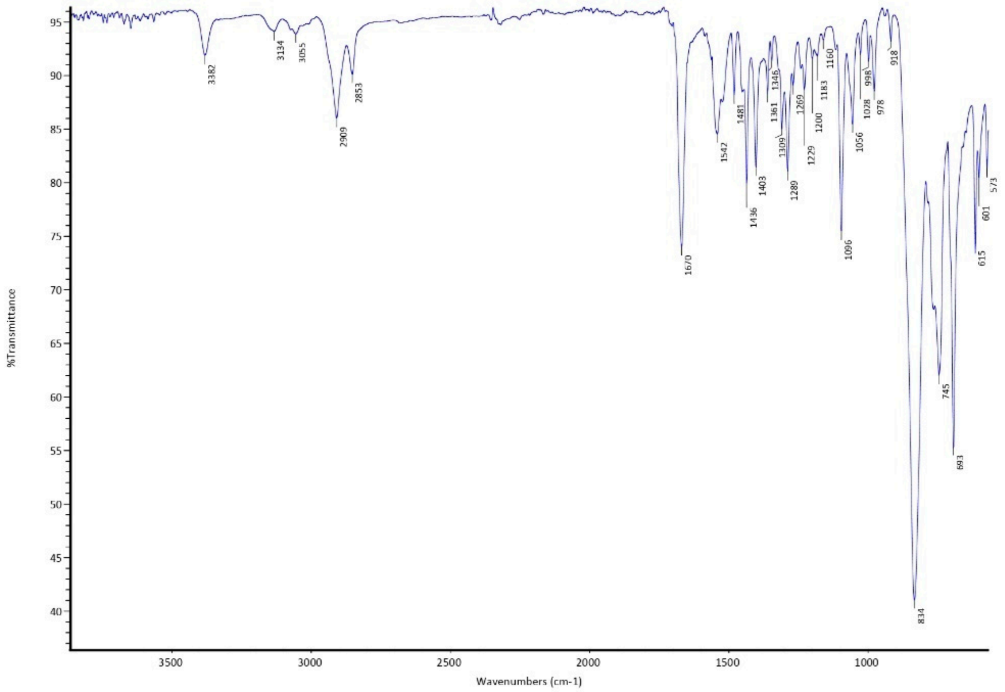

Figure S7. FT-IR spectrum of $[Cu(L^{Ad})(PPh_3)PF_6]$ (**4**).

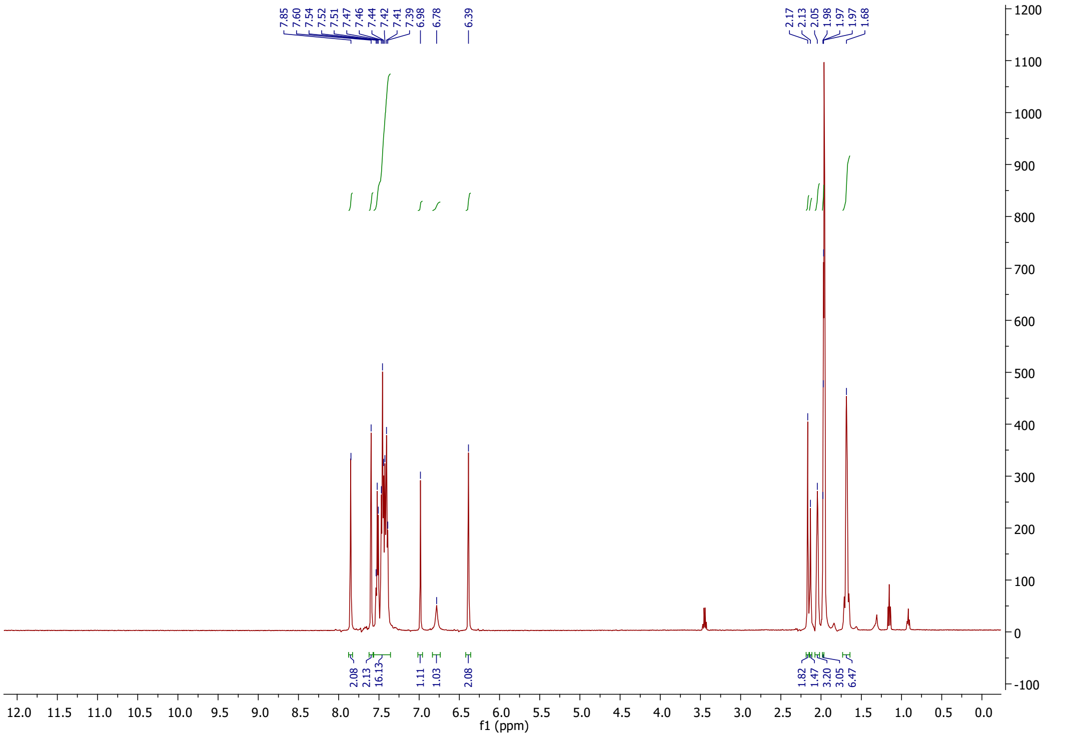

Figure S8. ^{1}H-NMR spectrum of $[Cu(L^{Ad})(PPh_3)]PF_6$ (**4**) in CD_3CN.

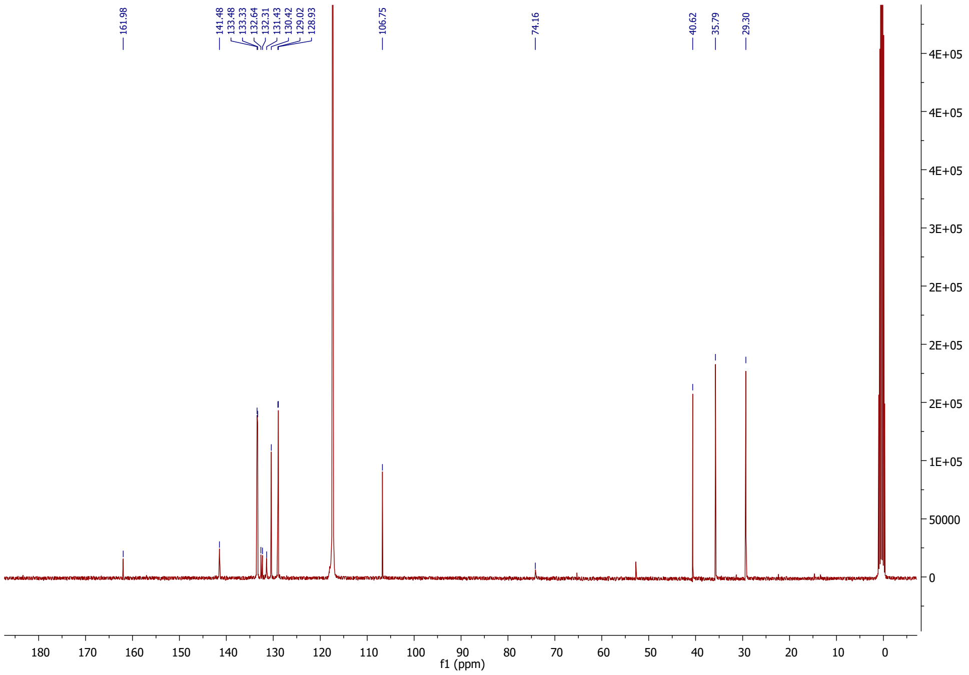

Figure S9. $^{13}C\{^1H\}$-NMR spectrum of $[Cu(L^{Ad})(PPh_3)]PF_6$ (**4**) in CD_3CN.

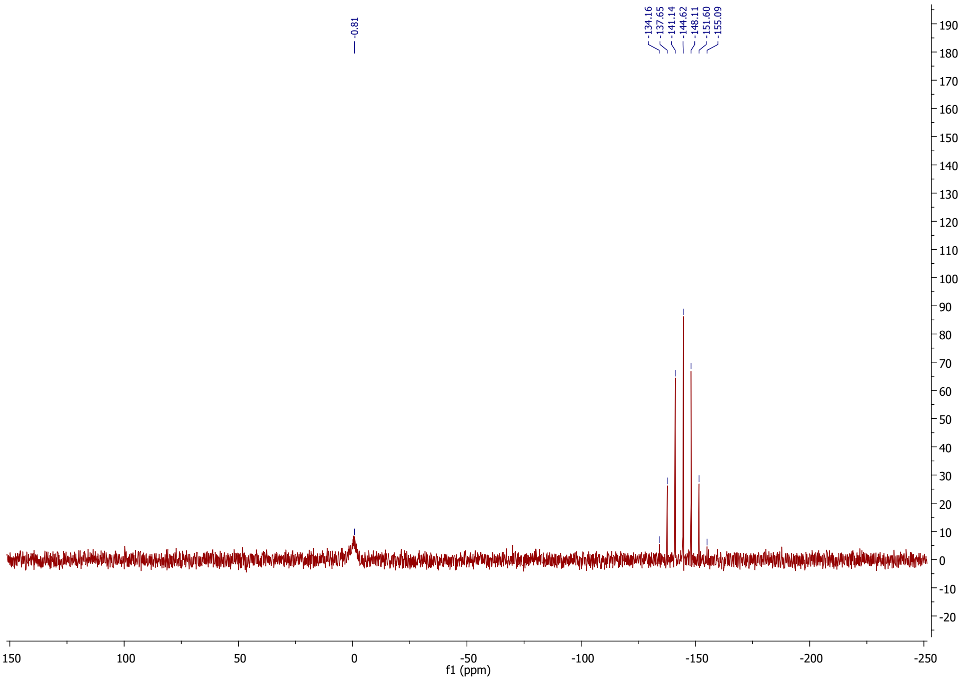

Figure S10. $^{31}P\{^1H\}$-NMR spectrum of $[Cu(L^{Ad})(PPh_3)]PF_6$ (**4**) in CD_3CN.

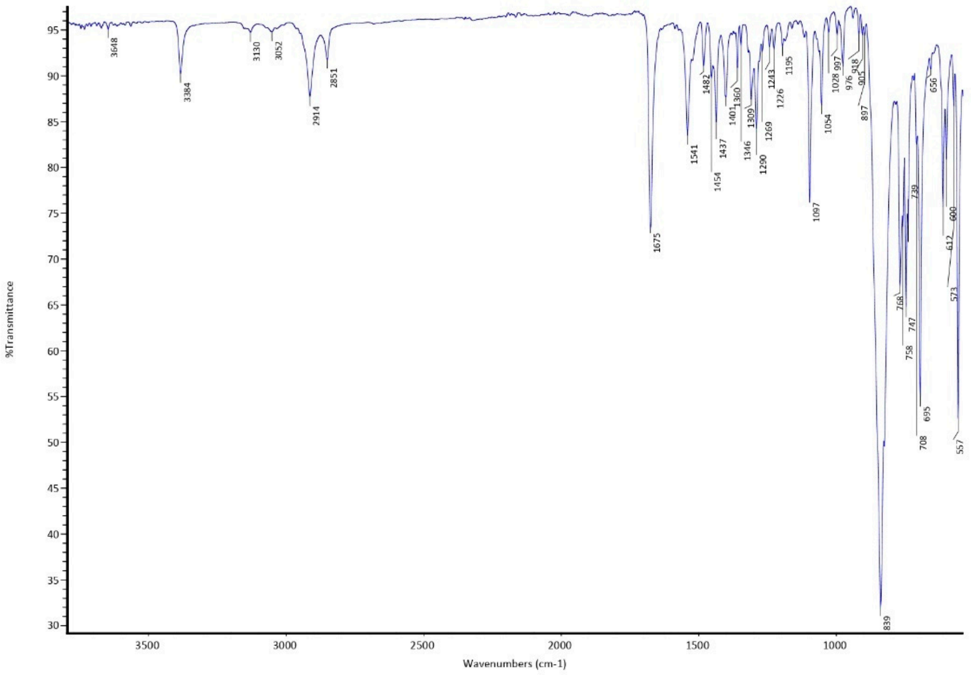

Figure S11. FT-IR spectrum of [Cu(L^{Ad})(PPh$_3$)$_2$]PF$_6$ (**5**).

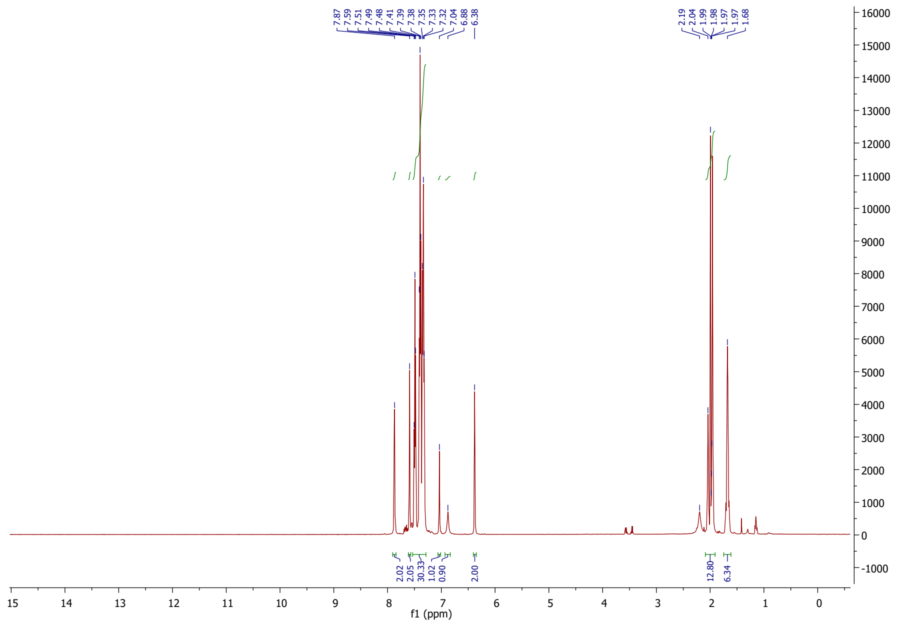

Figure S12. ^{1}H-NMR spectrum of [Cu(L^{Ad})(PPh$_3$)$_2$]PF$_6$ (**5**) in CD$_3$CN.

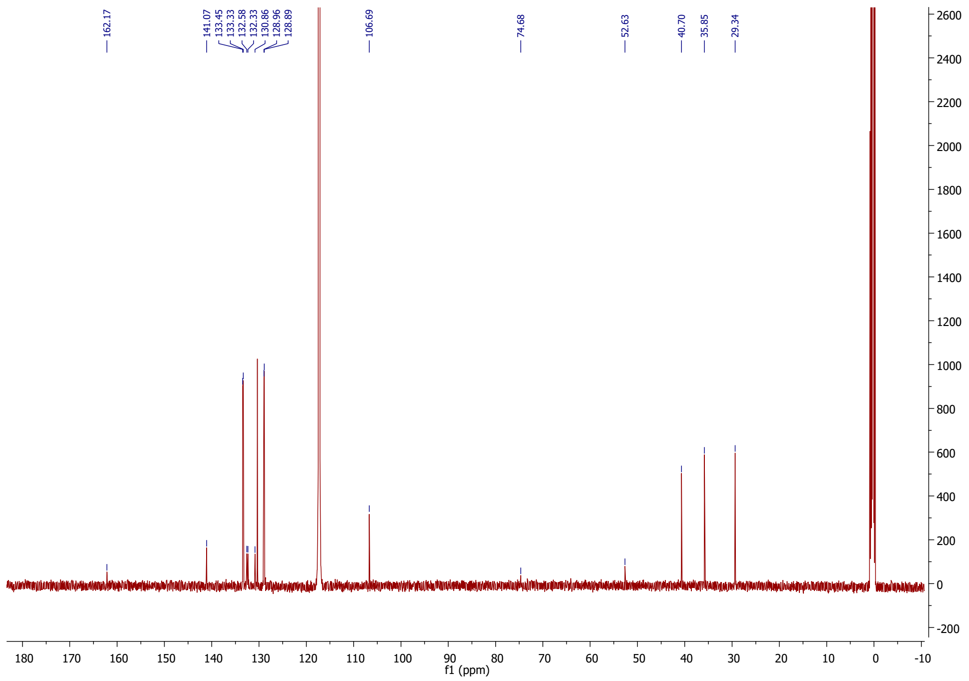

Figure S13. $^{13}C\{^1H\}$-NMR spectrum of in $[Cu(L^{Ad})(PPh_3)_2]PF_6$ (**5**) in CD_3CN.

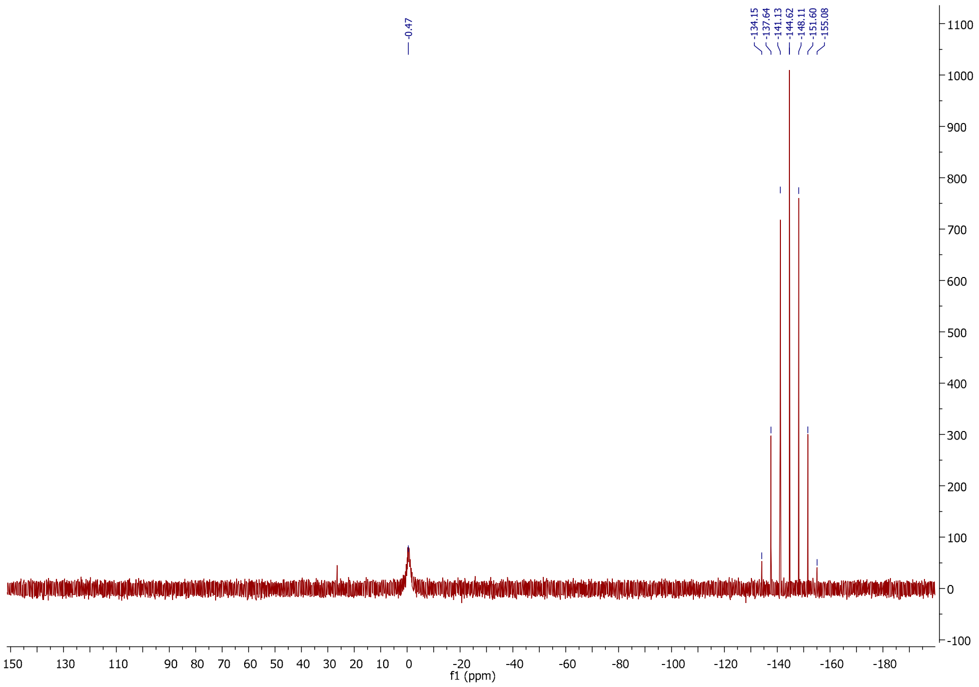

Figure S14. $^{31}P\{^1H\}$-NMR spectrum of $[Cu(L^{Ad})(PPh_3)_2]PF_6$ (**5**) in CD_3CN.

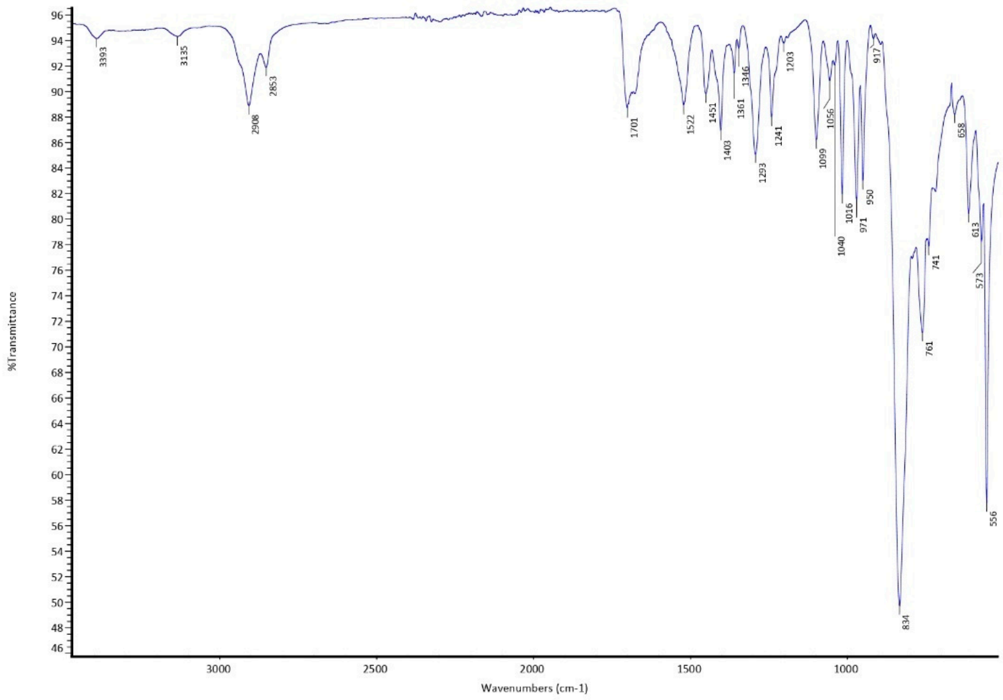

Figure S15. FT-IR spectrum of $[Cu(L^{Ad})(PTA)]PF_6$ (**6**).

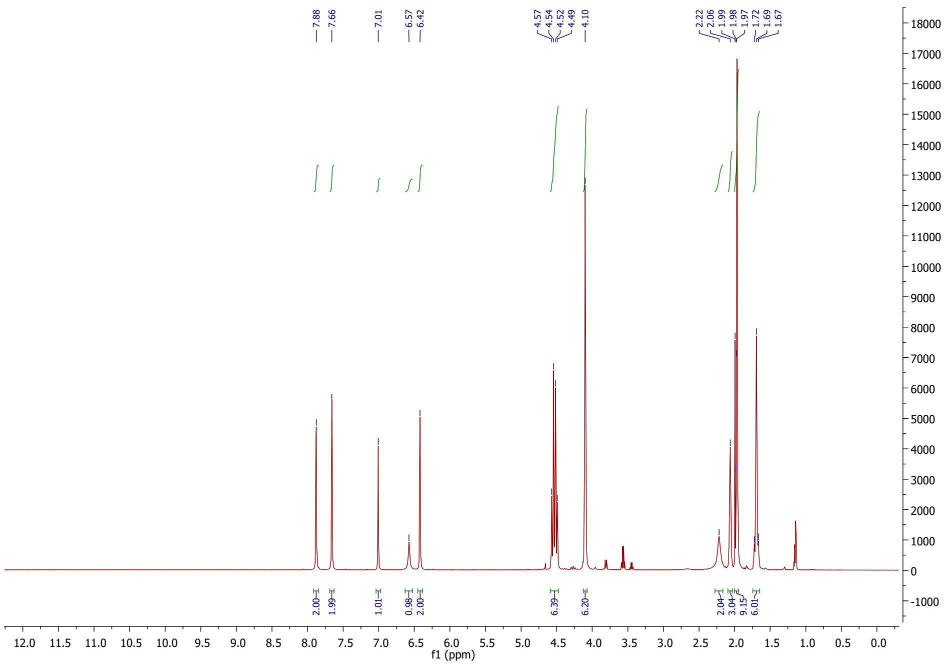

Figure S16. ^{1}H-NMR spectrum of $[Cu(L^{Ad})(PTA)]PF_6$ (**6**) in CD_3CN.

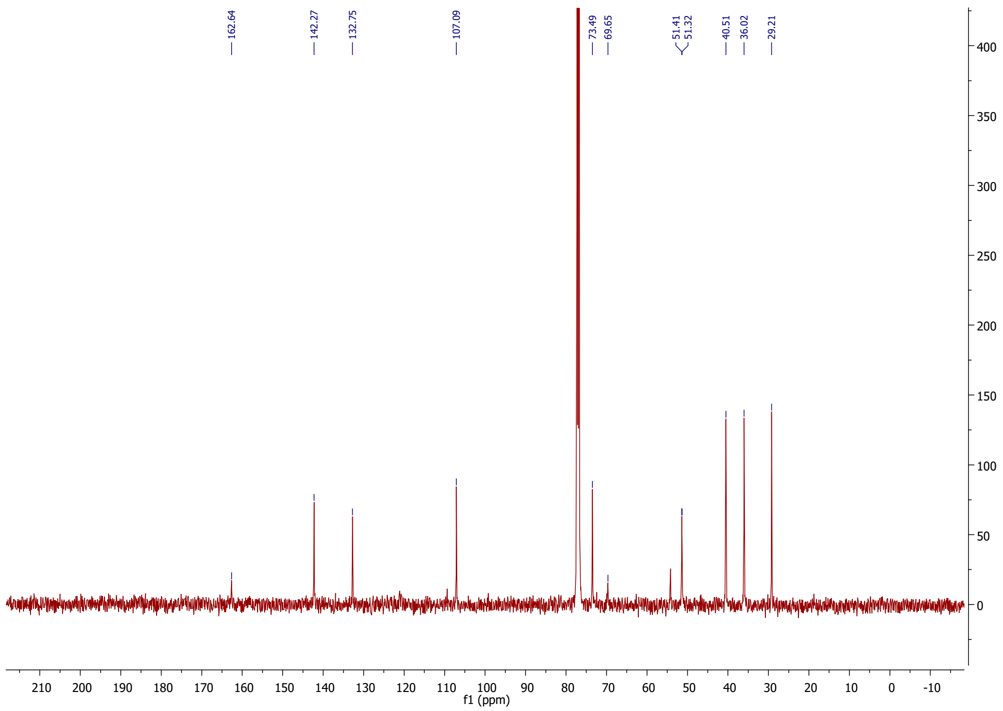

Figure S17. $^{13}C\{^1H\}$-NMR spectrum of in [Cu(L^{Ad})(PTA)]PF_6 (**6**) in $CDCl_3$.

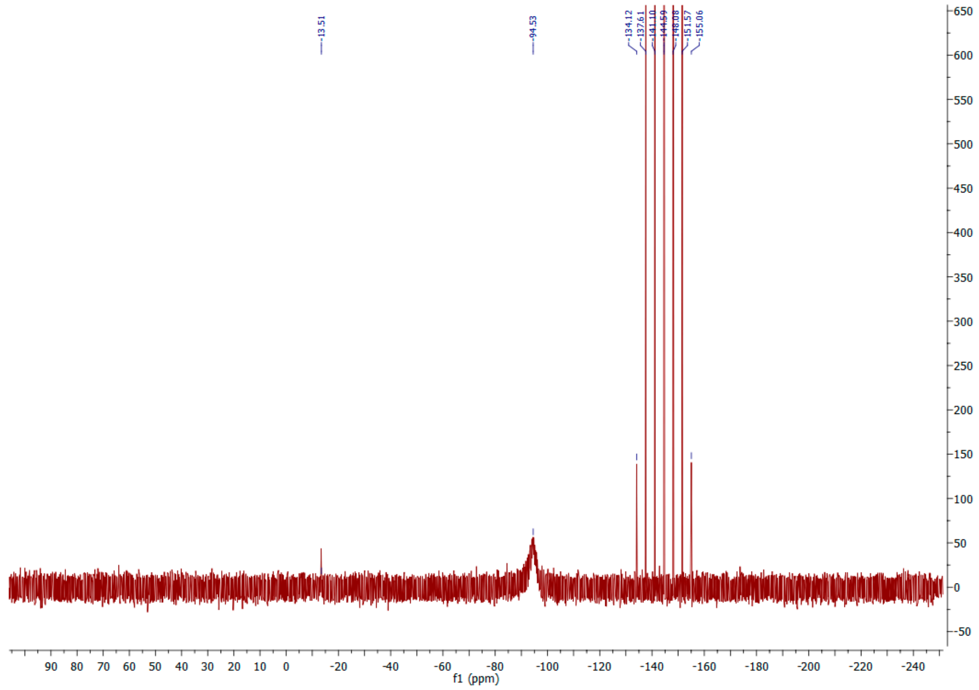

Figure S18. $^{31}P\{^1H\}$-NMR spectrum of [Cu(L^{Ad})(PTA)]PF_6 (**6**) in CD_3CN.

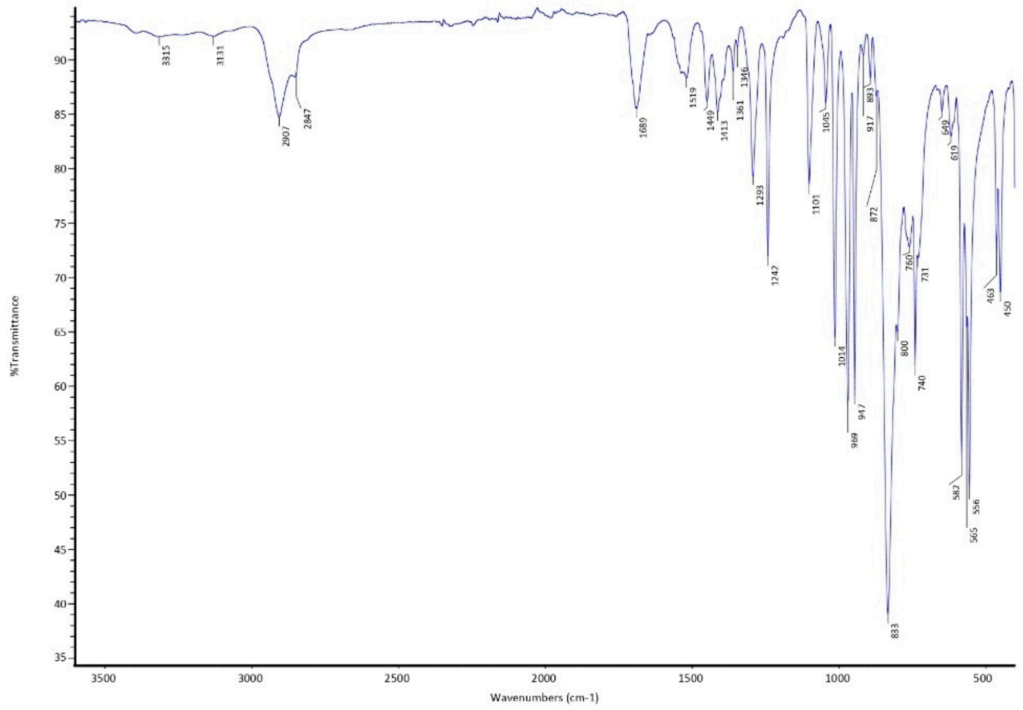

Figure S19. FT-IR spectrum of [Cu(L^{Ad})($PTA)_2$]PF_6 (**7**).

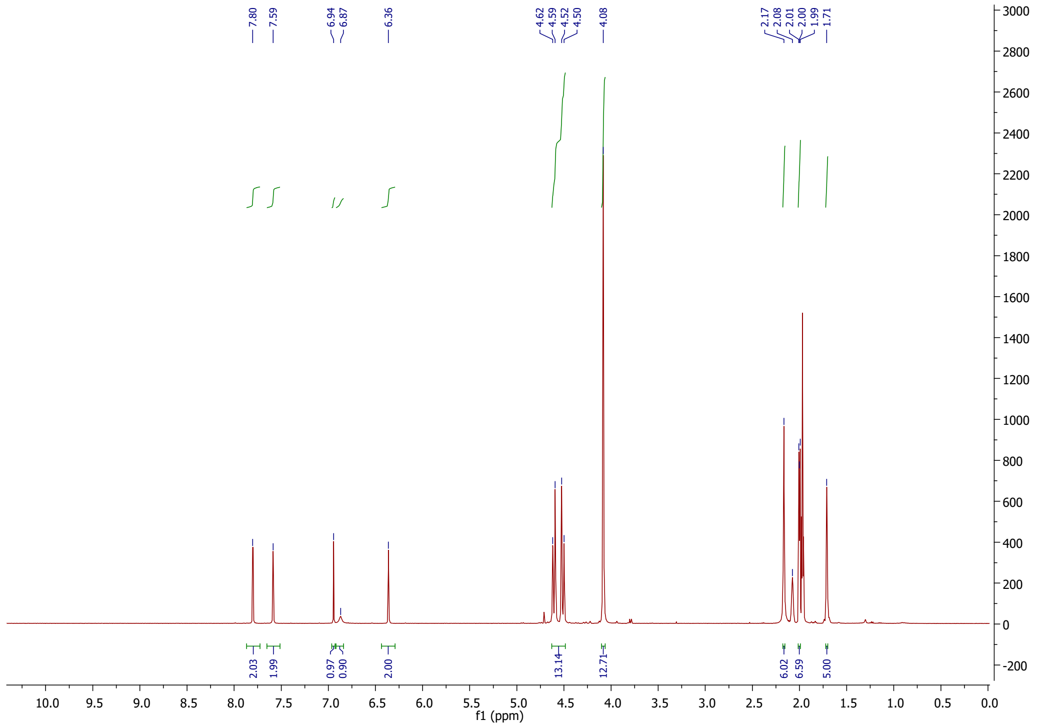

Figure S20. ^{1}H-NMR spectrum of [Cu(L^{Ad})($PTA)_2$]PF_6 (**7**) in CD_3CN.

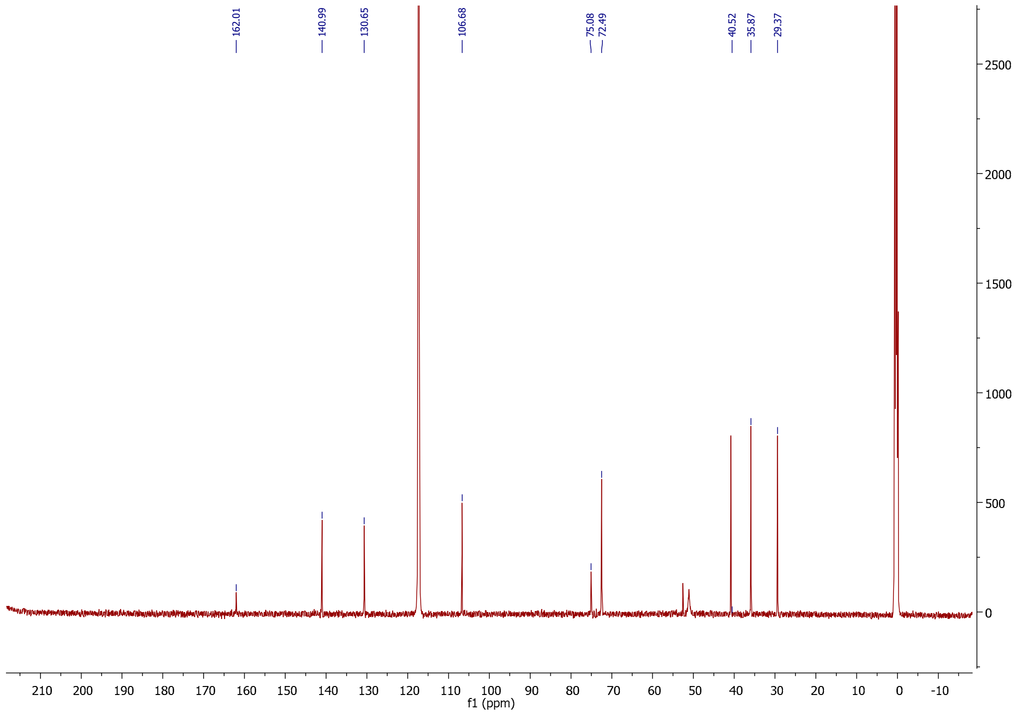

Figure S21. $^{13}C\{^{1}H\}$-NMR spectrum of in $[Cu(L^{Ad})(PTA)_2]PF_6$ (**7**) in CD_3CN.

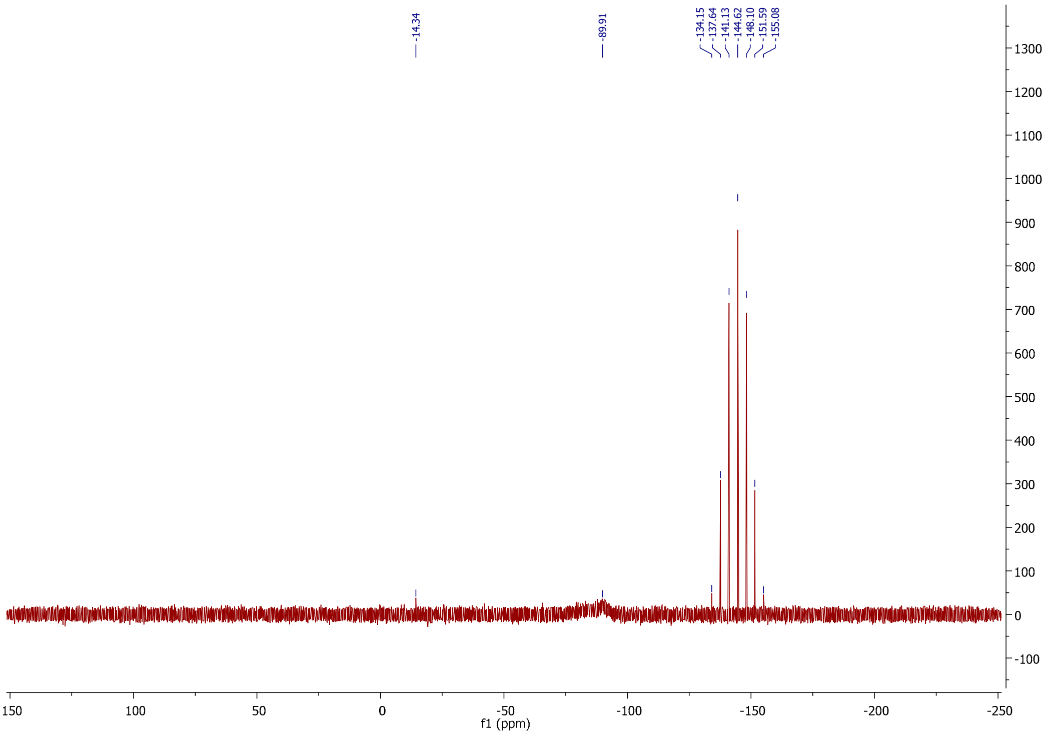

Figure S22. $^{31}P\{^{1}H\}$-NMR spectrum of $[Cu(L^{Ad})(PTA)_2]PF_6$ (**7**) in CD_3CN.

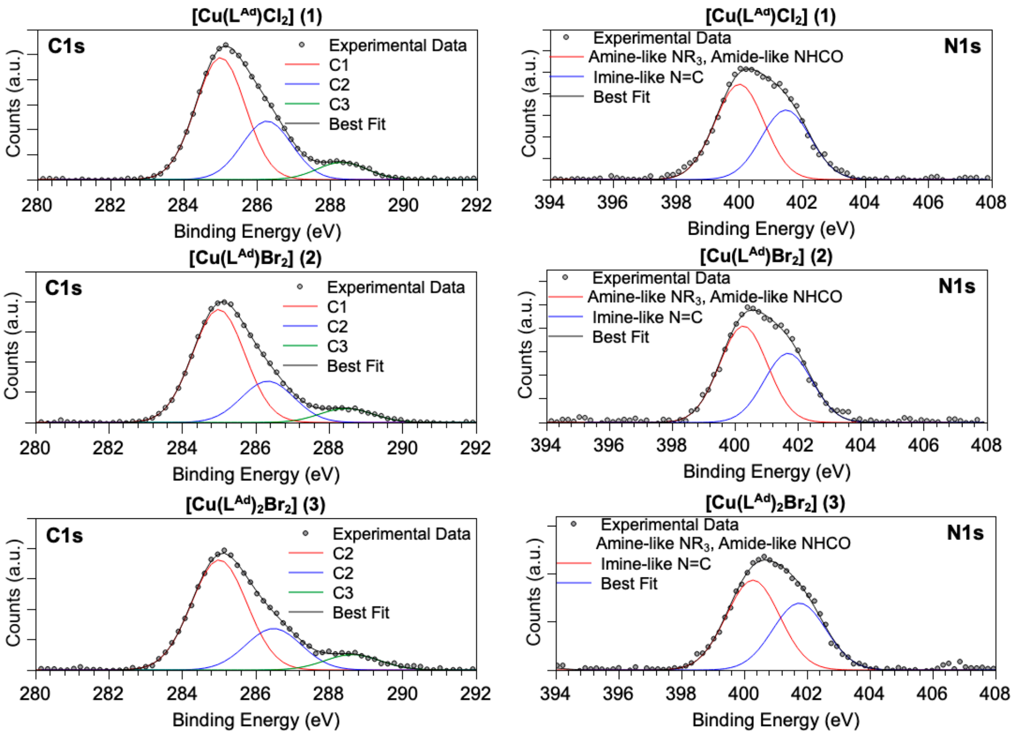

Figure S23. C1s (left) and N1s (right) spectra collected on the Cu(II) coordination compounds **1**, **2** and **3** (from top to bottom).

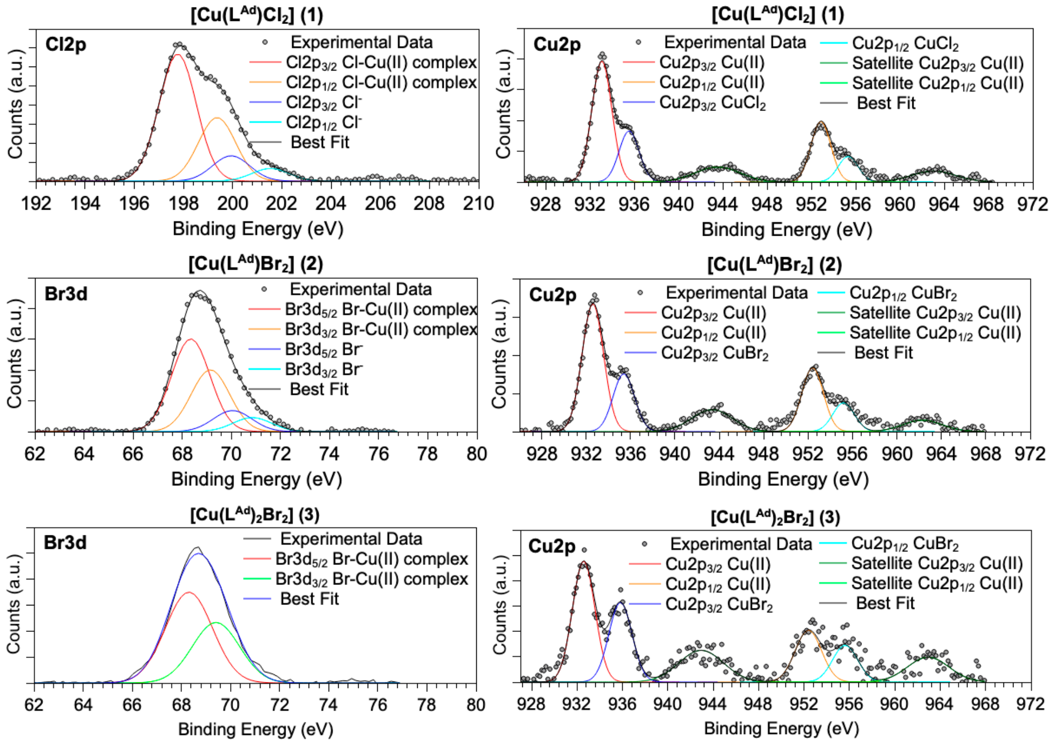

Figure S24. Cl2p or Br3d (left) and Cu2p (right) spectra collected on the Cu(II) coordination compounds **1**, **2** and **3** (from top to bottom).

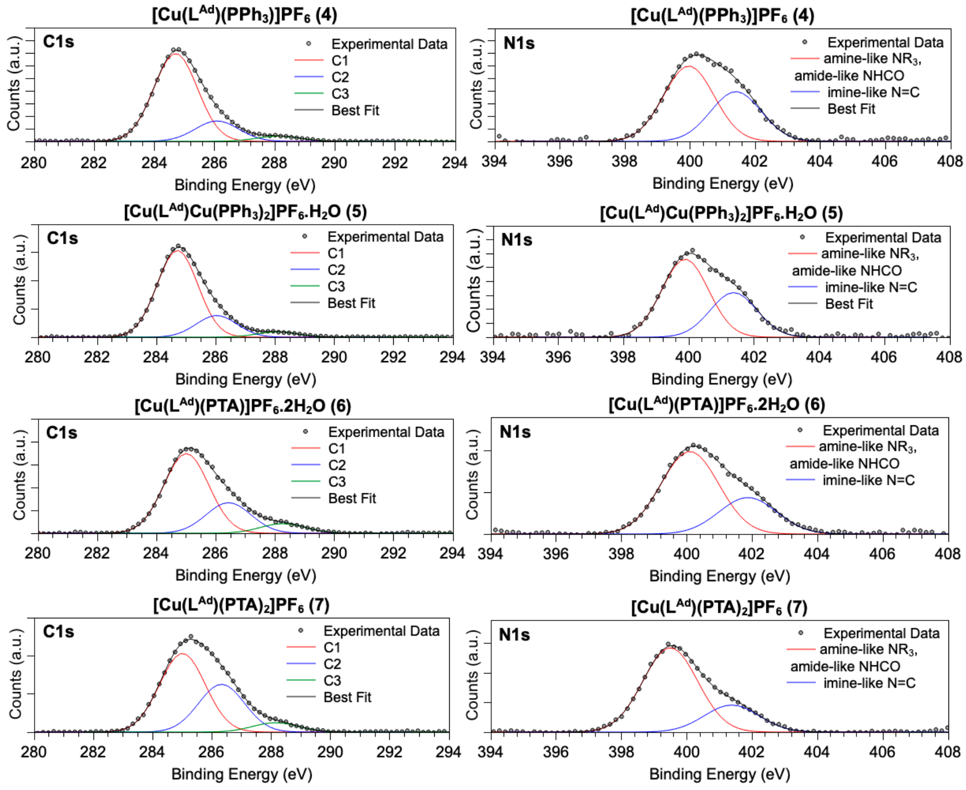

Figure S25. C1s (left) and N1s (right) spectra collected on the Cu(I) coordination compounds **4**, **5**, **6** and **7** (from top to bottom).

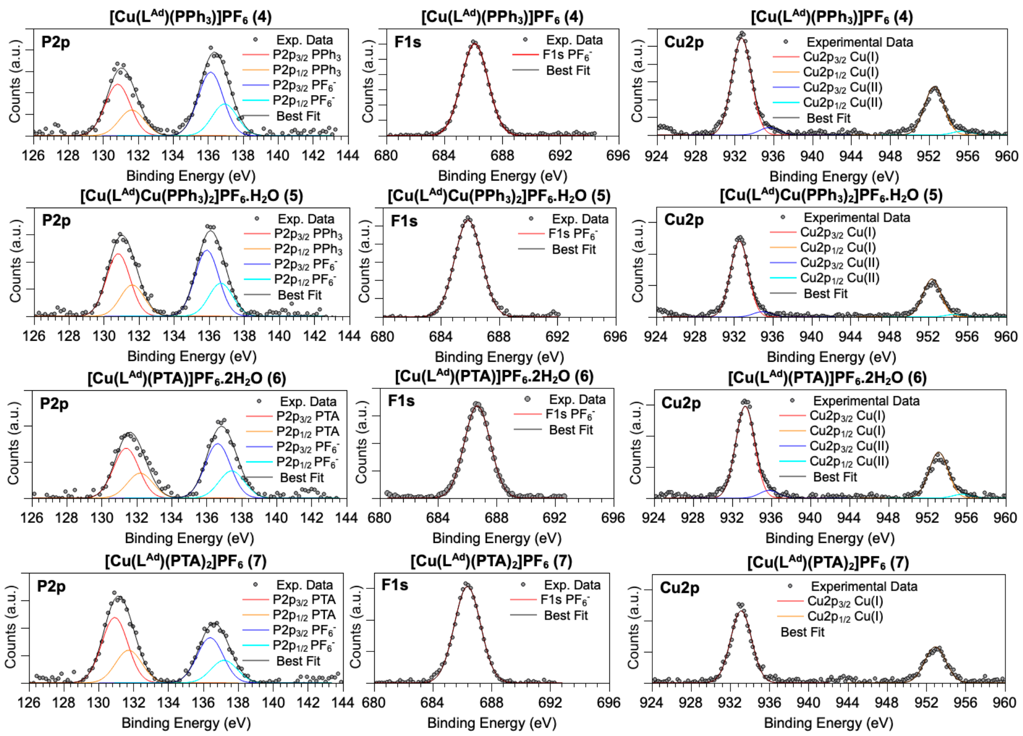

Figure S26. P2p (left), F1s (middle) and Cu2p (right) spectra collected on the Cu(I) coordination compounds **4**, **5**, **6** and **7** (from top to bottom).

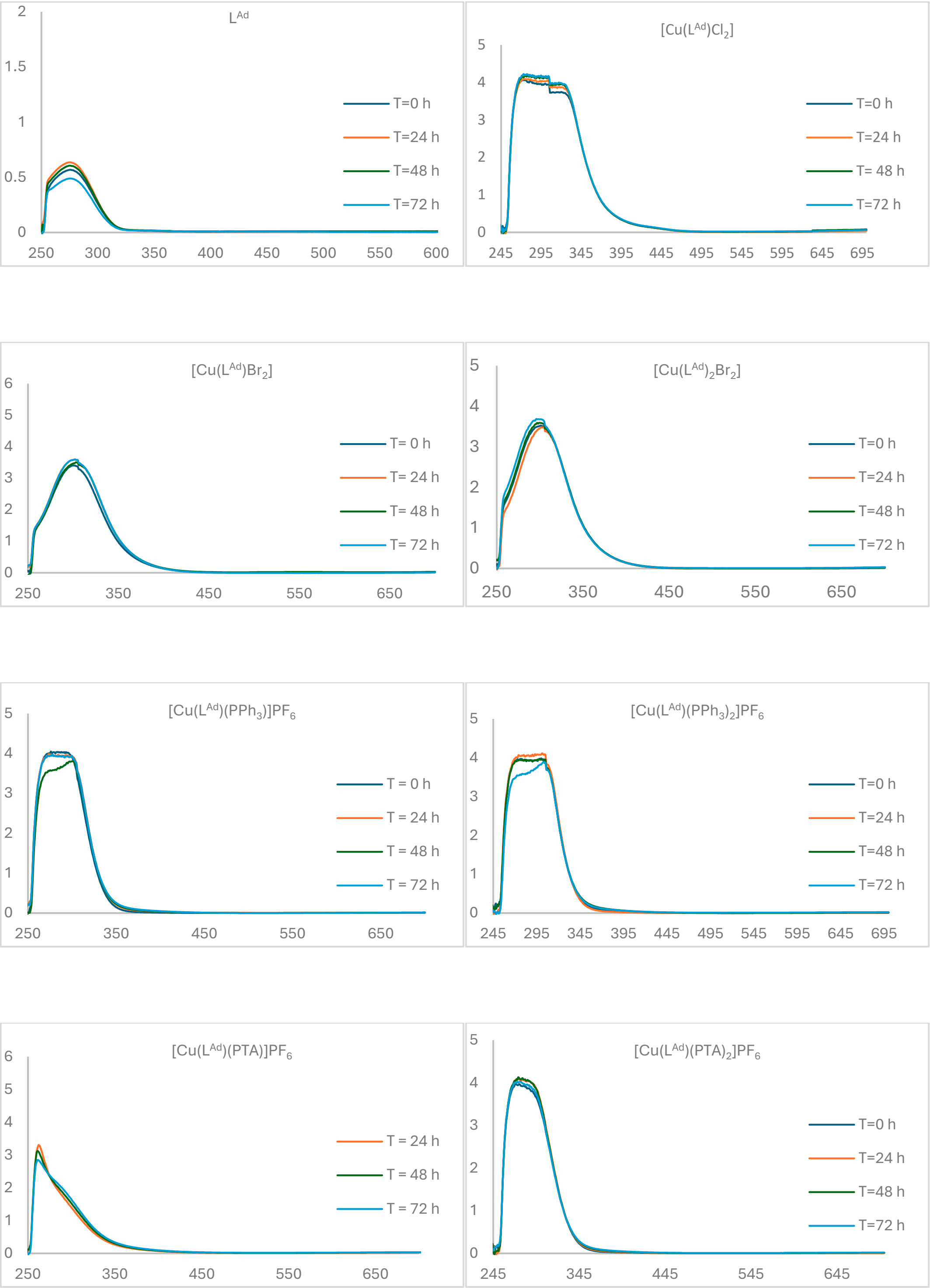

Figure S27. Stability studies. 2 mmol of L^{Ad} and complexes **1-7** were dissolved in 25 mL of DMSO. UV-Visible spectra were recorded at t = 0 min, t = 24 h, t = 48 h and t = 72 h.

A

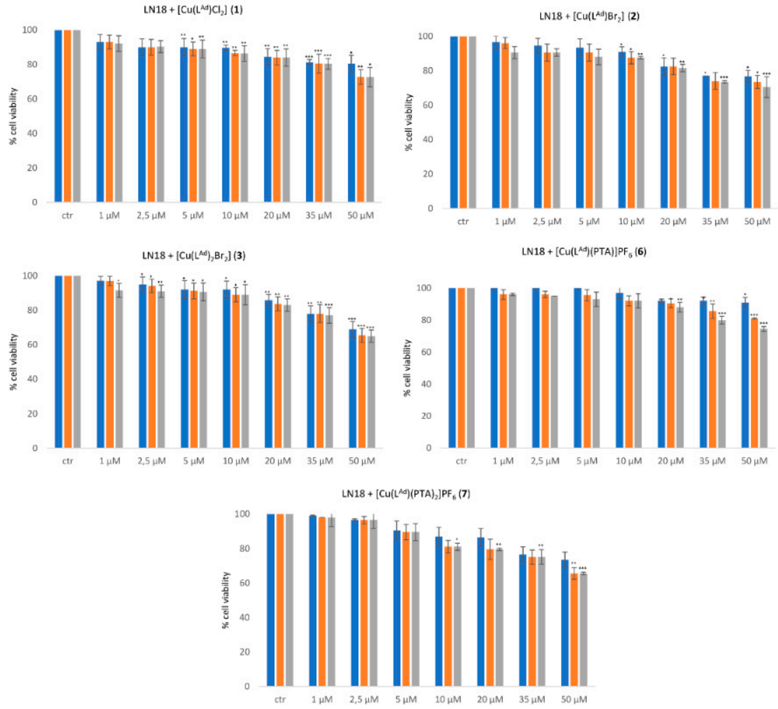

B

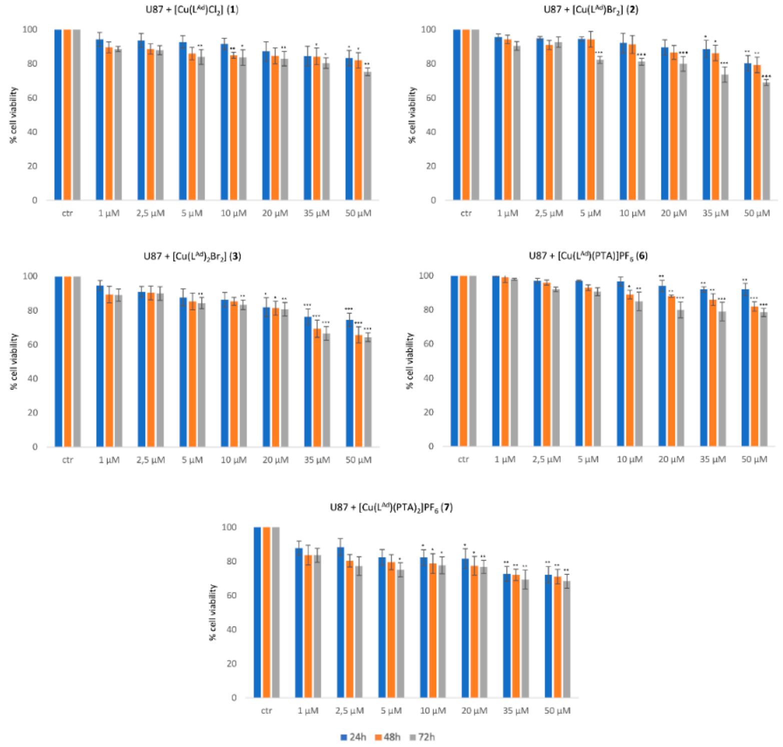

Figure S28. Effects of Cu compounds **1**, **2**, **3**, **6**, and **7** on cell viability evaluated by MTT assay. Histograms show the viability of LN18 (a) and U87 (b) cell lines after treatment at increasing concentrations (up to 50 µM) for 24, 48, and 72 hours. Data represent the % of cell viability with respect to the control (set at 100% value). Results are expressed as mean ± SD values from three independent experiments. *$p < 0.05$, **$p < 0.01$, ***$p < 0.001$ (two-tailed Student's t-test).

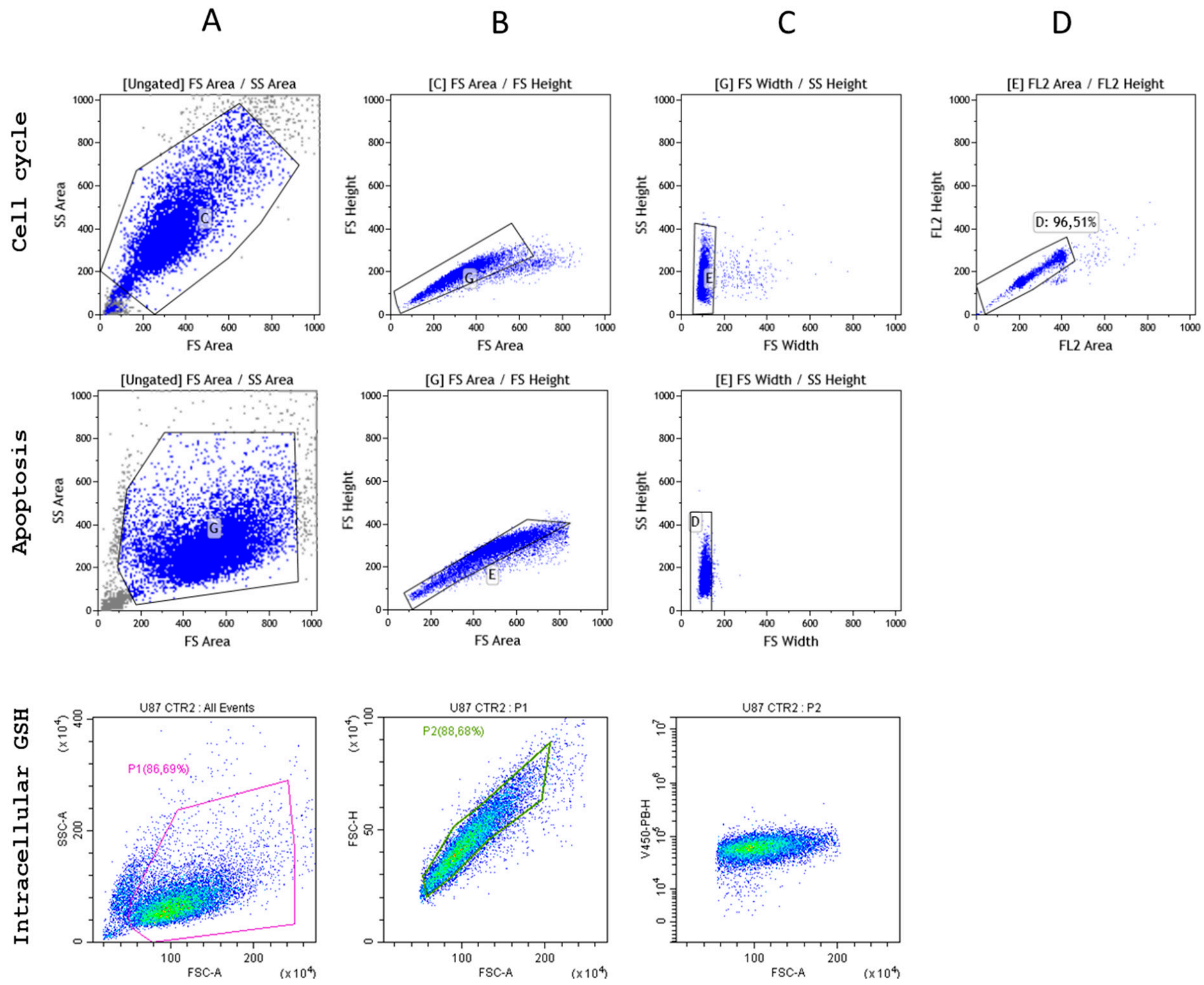

Figure S29. Gating strategy of the cell population for cell cycle, apoptosis, and intracellular GSH level evaluation study in cytofluorimetry. Cells were first gated on forward scattering area (FSC-A) versus side scattering area (SSC-A) signals to exclude debris (Panel A). FSC-A versus FSC height (Panel B) and FSC width versus SSC height (Panel C) gating strategies were used to exclude doublet events and cell clumps. In addition, an FSC versus V450-PBH dot plot (Panel C) was used to identify and exclude non-viable cells stained with Sytox Green. In Panel D, an FL-2 area versus FL-2 height gate dot plot was used to define the total cell population in the different phases of the cell cycle.

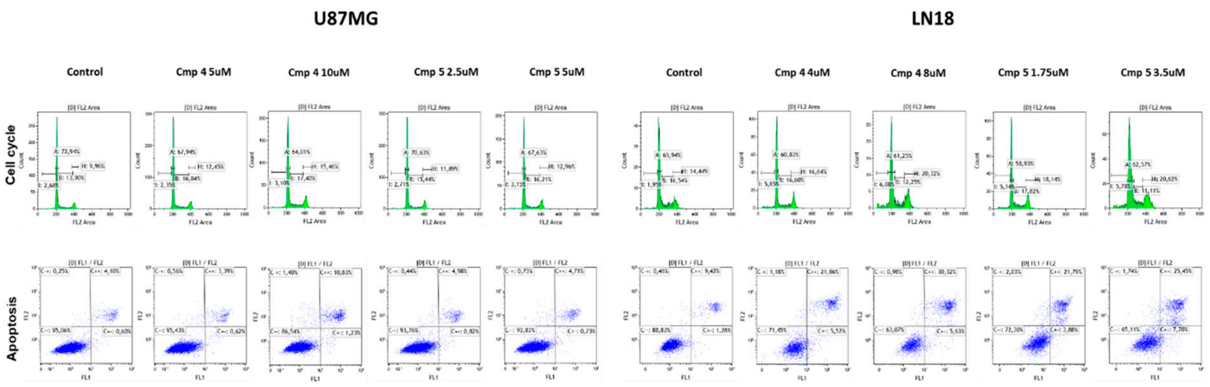

Figure S30. Effects of the Cu compounds **4** and **5** on the cell cycle distribution and apoptosis after 48 hours. Representative histograms and dot plots show cell cycle profiles and apoptosis analysis in U87 MG and LN18 glioblastoma cell lines after 48 hours of treatment with compounds **4** and **5**.

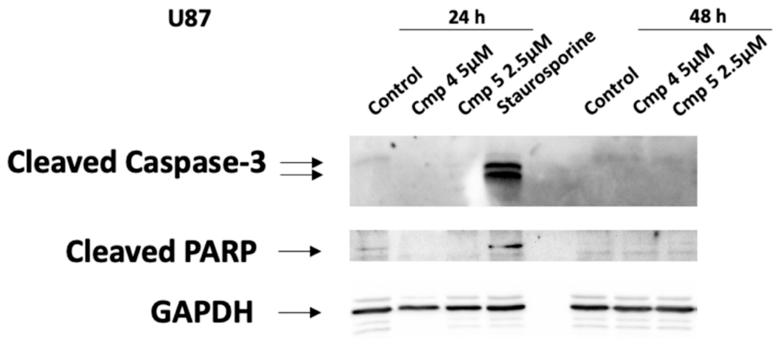

Figure S31. Western blot analysis of key proteins involved in apoptotic signaling and its regulation in U87 treated cells. Analysis of cleaved caspase-3 and cleaved PARP-1 protein expression in U87 MG cells after 24 and 48 h of treatment with compounds **4** and **5**. GAPDH was used as a loading control.

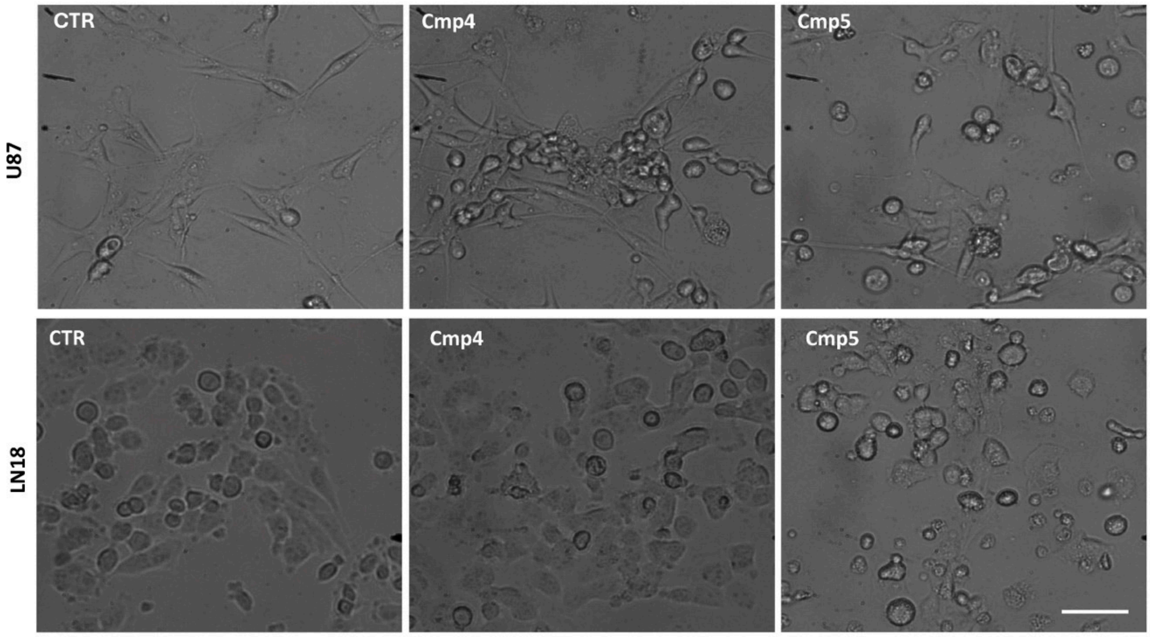

Figure S32. Phase-contrast microscopy of U87 MG and LN18 cells. Control cells maintained their normal morphology and confluency, whereas treatment with compounds **4** and **5** at their respective IC_{50} doses for 24 hours resulted in cell rounding, detachment, and reduced cell density. Scale bar: 50 µm.

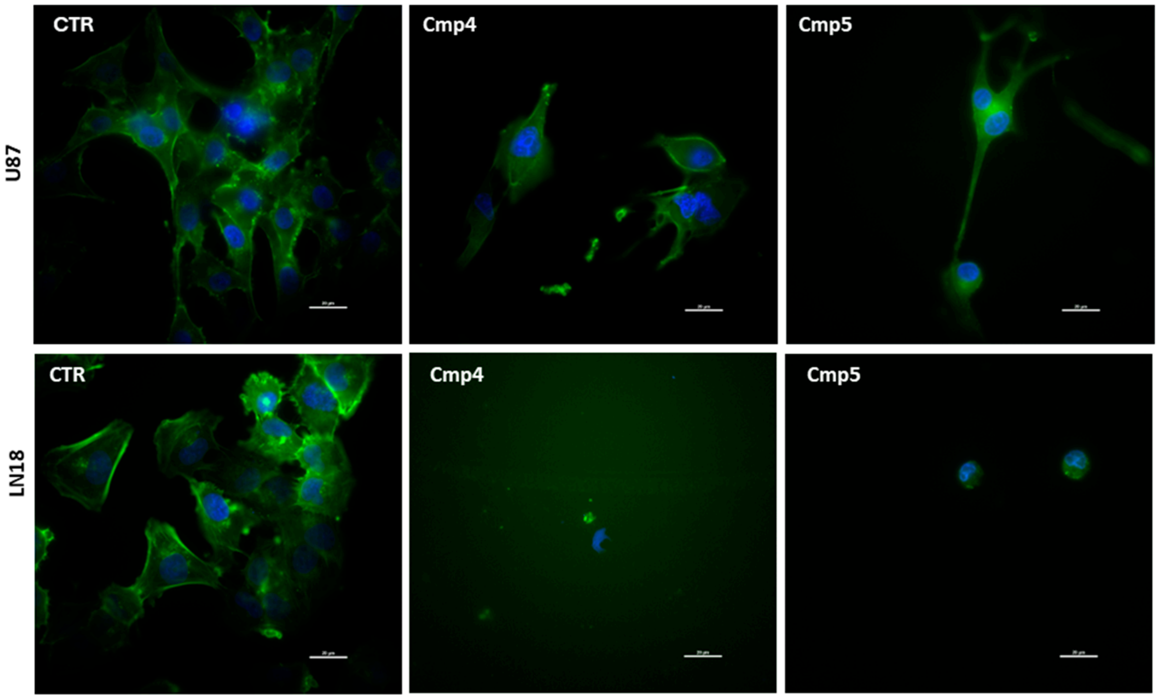

Figure S33. Immunofluorescence staining of actin and nuclei in U87 MG and LN18 cells. After 24 h of treatment with compounds **4** and **5** at their respective IC_{50} doses, actin filaments (phalloidin-FITC, green) and nuclei (Hoechst, blue) show cytoskeletal disruption and nuclear condensation in treated cells. Scale bar: 20 µm.

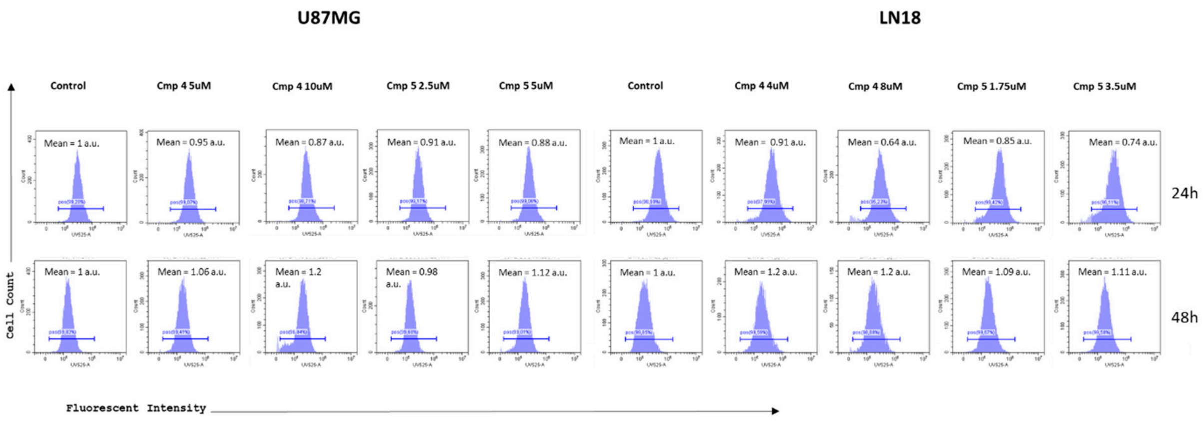

Figure S34. Effects of the compounds **4** and **5** on intracellular GSH levels after 24 and 48 hours. Representative histograms of intracellular GSH levels in U87 MG and LN18 glioblastoma cell lines following 24 and 48 hours of treatment with compounds **4** and **5**. Mean fluorescence intensity (MFI) values are normalized to the untreated control (set to 1). GSH levels were measured by MCB staining.

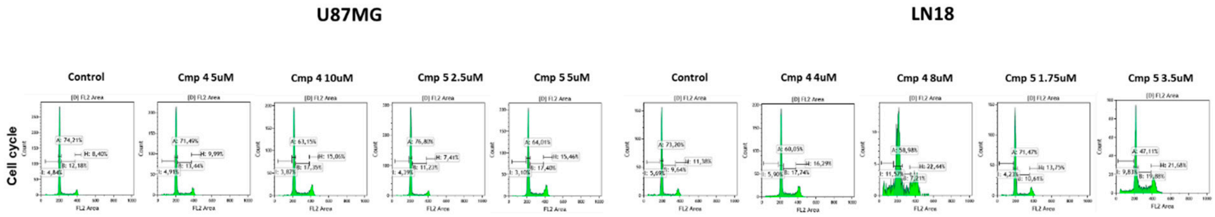

Figure S35. Effects of compounds **4** and **5** on the cell cycle distribution after 72 hours. Representative histograms showing cell cycle profiles of U87 MG and LN18 glioblastoma cell lines after 72 hours of treatment with compounds **4** and **5**.

Table S1. XPS data analysis results (BE, FWHM, experimental and calculated atomic ratio values, and proposed assignments) for samples **1-7**.

Sample	Signal	BE (eV)	FWHM (eV)	atomic ratios (exp.)	atomic ratios (theor.)	Assignment
**1**	C1s	285.00<br>286.27<br>288.31	1.59<br>1.59<br>1.59	7.0<br>3.4<br>1.0	15<br>6<br>1	C-C + C=C (C1)<br>C-N (C2)<br>NC=O. (C3)
	N1s	400.00<br>401.48	1.83<br>1.83	1.4<br>1.0	1.5<br>1	N-C amine-like<br>N=C imine-like
	Cl2p$_{3/2}$	197.77<br>199.94	1.78<br>1.78	1.0 Cl/Cu(II) = 1.9<br>0.2	Cl/Cu(II) = 2	Cl-Cu complex<br>$CuCl_2$
	Cu2p$_{3/2}$	933.00<br>935.54<br><br>943.40	2.31<br>2.31<br><br>5.05	1.0 N/Cu(II) = 5.4<br>0.4	1 N/Cu(II) = 5<br>0	Cu(II) complex<br>Cu(II) $CuCl_2$<br><br>Cu(II) satellite
	O1s	531.70<br>533.36	1.73<br>1.73	1.0<br>0.2	1<br>0	O=C (+ impurities)<br>O-C (impurities)
**2**	C1s	285.00<br>286.34<br>288.47	1.67<br>1.67<br>1.67	8.0<br>3.0<br>1.0	15<br>6<br>1	C-C + C=C (C1)<br>C-N (C2)<br>NC=O + imp. (C3)
	N1s	400.27<br>401.69	1.78<br>1.78	1.4<br>1.0	1.5<br>1	N-C amine-like<br>N=C imine-like
	Br3d$_{5/2}$	68.36<br>70.06	1.84<br>1.84	1.0 Br/Cu(I) = 2.5<br>0.2	Br/Cu(II) = 2	Br-Cu complex<br>$BrCl_2$
	Cu2p$_{3/2}$	932.62<br>935.40<br><br>943.36	2.28<br>2.28<br><br>4.32	1.0 N/Cu(II) = 5.9<br>0.4	1 N/Cu(II) = 5<br>0	Cu(II) complex<br>Cu(II) $CuBr_2$<br><br>Cu(II) satellite
	O1s	532.09<br>533.40	2.00<br>2.00	1.0	1	O=C (+ impurities)<br>Phys. H_2O
**3**	C1s	285.00<br>286.49<br>288.63	1.78<br>1.78<br>1.78	7.1<br>2.7<br>1.0	11<br>5<br>1	C-C + C=C (C1)<br>C-N (C2)<br>NC=O + imp. (C3)
	N1s	400.28<br>401.76	1.99<br>1.99	1.3<br>1.0	1.4<br>1.0	
	Br3d$_{5/2}$	68.30	2.25	1.0 Br/Cu(II) = 3.7	Br/Cu(II) = 2	Br-Cu complex
	Cu2p$_{3/2}$	932.58<br>935.82<br><br>942.93	2.34<br>2.34<br><br>4.63	1.0 N/Cu(II) = 8<br>0.7	N/Cu(II) = 10	Cu(II) complex<br>Cu(II) $CuBr_2$<br><br>Cu(II) satellite
	O1s	531.86<br>533.45	1.96<br>1.96	1.8<br>1.0	1,0<br>0	O=C (+ impurities)<br>O-C (impurities)
**4**	C1s	284.70<br>286.08<br>288.13	1.70<br>1.70<br>1.70	17.0<br>3.9<br>1.0	29<br>6<br>1	C-C+C=C+C-P (C1)<br>C-N (C2)<br>NC=O + imp. (C3)
	N1s	399.96<br>401.40	1.82<br>1.82	1.5<br>1.0<br>N/P1 = 4.2	1.5<br>1.0<br>N/P1 = 5	N-C amine-like<br>N=C imine-like

Sample	Signal	BE (eV)	FWHM (eV)	atomic ratios (exp.)	atomic ratios (theor.)	Assignment
	$P2p_{3/2}$	130.83<br>136.14	1.68<br>1.68	P1/Cu = 0.6	P1/Cu = 1	P-C PPh_3 (P1)<br>P-F PF_6 (P2)
	F1s	686.08	2.17	F/P2 = 6.0	F/P2 = 6	F-P PF_6
	$Cu2p_{3/2}$	932.70<br>935.56	2.36<br>2.36	1 N/Cu(I) = 2.5<br>0.07	1 N/Cu(I) = 5<br>0	Cu(I)<br>Cu(II)
	O1s	531.45<br>533.04	2.43<br>2.43	1.0<br>0.4	1<br>0	O=C (+ impurities)<br>O-C (impurities)
**5**	C1s	284.70<br>286.02<br>288.07	1.68<br>1.68<br>1.68	17.8<br>4.0<br>1.0	47<br>6<br>1	C-C+C=C+C-P (C1)<br>C-N (C2)<br>NC=O + imp. (C3)
	N1s	399.88<br>401.37	1.68<br>1.68	1.7<br>1.0<br>N/P1 = 3.8	1.5<br>1.0<br>N/P1 = 2.5	N-C amine-like<br>N=C imine-like
	$P2p_{3/2}$	130.80<br>135.86	1.59<br>1.59	P1/Cu = 0.7	P1/Cu = 2	P-C PPh_3 (P1)<br>P-F PF_6 (P2)
	F1s	685.87	2.20	F/P2 = 5.9	F/P2 = 6	F-P PF_6
	$Cu2p_{3/2}$	932.55<br>934.90	2.17<br>2.17	1 N/Cu(I) = 2.5<br>0.07	1 N/Cu(I) = 5<br>0	Cu(I)<br>Cu(II)
	O1s	531.66<br>533.05	1.82<br>1.82	1.0<br>0.5	1<br>0	O=C (+ impurities)<br>O-C (impurities)
**6**	C1s	285.00<br>286.42<br>288.26	1.77<br>1.77<br>1.77	8.3<br>3.2<br>1.0	14<br>9<br>1	C-C+C=C+C-P (C1)<br>C-N (C2)<br>NC=O + imp. (C3)
	N1s	400.09<br>401.86	2.07<br>2.07	2.2<br>1.0<br>N/P1 = 7.2	2.5<br>1.0<br>N/P1 = 8	N-C amine-like<br>N=C imine-like
	$P2p_{3/2}$	131.39<br>136.63	1.71<br>1.71	P1/Cu = 0.7	P1/Cu = 1	P-C PTA (P1)<br>P-F PF_6 (P2)
	F1s	686.66	2.09	F/P2 = 5.3	F/P2 = 6	F-P PF_6
	$Cu2p_{3/2}$	933.32<br>935.77	1.71<br>1.71	1 N/Cu(I) = 4.8<br>0.08	1 N/Cu(I) = 8<br>0	Cu(I)<br>Cu(II)
	O1s	532.17<br>534.16	2.17<br>2.17	1.0	1	O=C (+ impurities)<br>Phys. H_2O
**7**	C1s	285.00<br>286.32<br>288.15	1.81<br>1.81<br>1.81	8.4<br>5.0<br>1.0	17<br>12<br>1	C-C+C=C+C-P (C1)<br>C-N (C2)<br>NC=O + imp. (C3)
	N1s	399.49<br>401.37	1.96<br>1.96	3.1<br>1.0<br>N/P1 = 4.1	4.5<br>1.0<br>N/P1 = 5.5	N-C amine-like<br>N=C imine-like
	$P2p_{3/2}$	130.91<br>136.38	1.76<br>1.76	P1/Cu = 1.4	P1/Cu = 2	P-C PTA (P1)<br>P-F PF_6 (P2)
	F1s	686.39	2.24	F/P2 = 4.8	F/P2 = 6	F-P PF_6
	$Cu2p_{3/2}$	933.09	2.53	1 N/Cu(I) = 5.7	1 N/Cu(I) = 11	Cu(I)
	O1s	531.71<br>533.18	2.82<br>2.82	1.0<br>0.1	1<br>0	O=C (+ impurities)<br>O-C (impurities)

Table S2. Optimized geometries for complexes **1-7** in xyz format.

#Cu complex **1**

26

N	-0.86842	-1.02354	-2.23111
H	-0.80523	1.36496	-4.52408
C	-1.42177	0.07081	-2.78003
C	0.44233	-0.39317	-3.97116
C	-0.62596	0.49936	-3.87667
H	-2.36604	0.45933	-2.39551
H	1.31193	-0.35593	-4.62674
N	0.27031	-1.29352	-2.94234
H	-0.89240	-6.21617	-4.82699
N	-0.81805	-4.08989	-2.29234
N	0.26273	-3.71508	-3.05505
C	-1.39388	-5.13230	-2.91290
C	0.36222	-4.49533	-4.18972
C	-0.67942	-5.41976	-4.10535
H	1.11826	-4.36475	-4.96328
Cu	-1.58938	-2.60295	-0.97504
C	0.96020	-2.52672	-2.57915
C	2.48784	-2.54492	-2.71504
O	3.13827	-3.53748	-2.97937
N	2.98845	-1.35800	-2.33225
H	0.84723	-2.57557	-1.45721
Cl	0.00363	-2.60209	0.59365
Cl	-3.78154	-2.61992	-1.21350
H	3.52432	-1.47639	-1.46063
H	2.21645	-0.69494	-2.17328
H	-2.22421	-5.68924	-2.47286

#Cu complex **2**

26

N	-0.86842	-1.02354	-2.23111
H	-0.80523	1.36496	-4.52408
C	-1.42177	0.07081	-2.78003
C	0.44233	-0.39317	-3.97116
C	-0.62596	0.49936	-3.87667
H	3.52433	-1.47640	-1.46063
H	1.26403	-0.41867	-4.69712
N	0.27031	-1.29352	-2.94234
H	-0.89240	-6.21617	-4.82699
N	-0.81805	-4.08989	-2.29234
N	0.26273	-3.71508	-3.05505
C	-1.39388	-5.13230	-2.91290

C	0.36222	-4.49533	-4.18972
C	-0.67942	-5.41976	-4.10535
Br	-3.98292	-2.62433	-1.27844
H	-2.33540	0.54023	-2.35973
C	0.96020	-2.52672	-2.57915
C	2.48784	-2.54492	-2.71504
O	3.13827	-3.53748	-2.97937
N	2.98845	-1.35800	-2.33225
H	0.84723	-2.57557	-1.45721
H	1.15390	-4.37807	-4.95214
Br	0.15081	-2.63346	0.72341
H	2.21646	-0.69494	-2.17328
Cu	-1.58938	-2.60295	-0.97504
H	-2.27580	-5.57464	-2.42747

#Cu complex **3**

48

Cu	6.07683	5.69825	16.84927
H	7.78069	2.71502	16.95400
O	7.85133	5.69825	15.31481
N	6.90594	4.31494	18.06562
N	3.78634	5.57718	20.56884
H	8.37704	4.06219	13.25132
H	2.82329	5.59032	20.20379
N	5.36307	6.89431	14.27824
C	7.51605	5.69825	14.13030
C	4.28208	8.76505	14.51480
H	7.80234	8.65439	16.94531
H	7.50443	6.74673	13.91708
N	6.90594	7.08156	18.06562
H	3.83107	9.56039	14.34557
H	9.45505	5.04513	14.08845
N	5.24771	7.08156	15.63292
H	8.32259	1.83611	19.35297
H	4.73144	3.49276	12.49057
H	3.95594	4.68680	21.05807
N	6.79059	6.89431	19.42030
C	7.87157	2.63145	19.18374
C	6.01109	5.69825	13.76468
N	5.24771	4.31494	15.63292
H	6.22366	5.69824	20.91060
H	7.43676	3.52677	21.21319
O	4.30232	5.69825	18.38373
C	4.58006	3.16937	15.77294
C	4.28208	2.63145	14.51480
H	4.38808	8.66465	16.75733
C	4.77910	3.49645	13.58557

C	4.63760	5.69825	19.56824
H	4.68982	7.90553	12.48501
C	7.57360	3.16937	17.92560
C	7.57360	8.22713	17.92560
C	4.58006	8.22713	15.77294
C	4.77910	7.90005	13.58557
N	5.36307	4.50219	14.27824
C	7.37455	7.90005	20.11297
N	6.79059	4.50219	19.42030
H	8.32259	9.56039	19.35297
H	5.93000	5.69826	12.78794
C	7.37455	3.49645	20.11297
H	4.31112	2.73054	16.74770
N	8.52381	5.00022	13.65065
C	7.87157	8.76505	19.18374
C	6.14257	5.69825	19.93387
H	7.43463	7.89380	21.20722
H	3.83107	1.83611	14.34557

#Cu complex **4**

58

C	-2.24507	-14.19802	3.41014
C	-1.12472	-13.67490	4.10332
C	0.00311	-13.95168	3.32932
N	-0.46736	-14.59537	2.20072
C	-3.14709	-17.98321	-3.41013
H	-1.12810	-13.14398	5.06144
N	-1.83407	-14.76691	2.25923
C	-6.31752	-12.96538	0.23072
C	0.15622	-15.26015	1.06317
C	1.32856	-17.49723	1.69938
H	1.32502	-19.66666	2.18643
C	0.78152	-18.76907	1.87201
C	1.35516	-14.51710	0.42605
O	1.64400	-13.36959	0.69394
C	-5.40273	-14.02849	0.17776
N	0.29054	-16.69691	1.27323
H	-0.61498	-15.18393	0.26157
N	-0.88784	-17.40122	1.20024
C	-0.59849	-18.66968	1.55575
H	-2.48196	-17.95867	-1.33874
H	-6.36139	-16.15244	-2.12703
N	1.88435	-15.27023	-0.54314
H	-9.18094	-14.73741	0.89709
H	-8.39263	-12.38309	0.53801
P	-4.59178	-16.71178	0.24340
C	-7.67674	-13.21781	0.48960

Cu	-2.62049	-16.30692	1.18532
H	-4.33630	-13.82513	-0.01295
H	-5.96705	-11.93349	0.07512
C	-7.20838	-15.60601	0.62845
C	-5.84347	-15.35817	0.36844
H	-3.31529	-14.20168	3.65327
H	1.71884	-14.82178	-1.45550
H	1.44419	-16.20140	-0.53339
H	1.04612	-13.76019	3.56444
H	-6.10593	-16.67337	-4.55522
H	-7.56350	-16.63589	0.78554
H	2.35908	-17.12997	1.82353
H	-2.24066	-18.49276	-3.77169
C	-4.16110	-17.62129	-4.31421
H	-1.31770	-19.50318	1.54108
H	-4.05063	-17.84714	-5.38589
C	-3.28419	-17.68805	-2.04433
C	-6.64742	-20.30716	0.71491
C	-6.01845	-19.19561	0.12653
C	-5.43881	-18.19903	0.94015
H	-6.17078	-19.53508	4.02508
C	-8.11850	-14.53617	0.69094
C	-6.70643	-20.43090	2.11286
H	-7.20127	-21.30176	2.56925
C	-5.49144	-18.33503	2.34704
H	-5.97849	-19.10628	-0.96960
H	-7.09635	-21.08038	0.07272
C	-6.12861	-19.44160	2.92904
H	-5.03498	-17.56636	2.99212
C	-5.31300	-16.96405	-3.84917
C	-5.45869	-16.67240	-2.48232
C	-4.44587	-17.03822	-1.56918

#Cu complex **5**

92

N	-1.05322	-0.34231	-1.63497
H	-1.31886	0.35630	-4.88167
C	-1.80365	-0.08147	-2.71960
C	0.31207	-0.04217	-3.41622
C	-0.99530	0.12322	-3.86230
H	-2.89671	-0.06989	-2.63626
H	1.27634	-0.00336	-3.93166
N	0.23933	-0.30870	-2.07219
H	2.13822	-5.12460	-0.29251
N	0.26720	-2.40151	0.14936
N	1.27334	-1.98372	-0.67026
C	0.51546	-3.70403	0.38086

C	2.14176	-3.00182	-0.97112
C	1.68431	-4.12850	-0.29582
H	-2.31419	2.52317	3.82518
H	3.00144	-2.84664	-1.63179
C	1.28287	-0.58284	-1.08651
C	2.69286	-0.19793	-1.58773
O	3.13443	-0.50800	-2.67692
N	3.33684	0.47367	-0.62692
H	1.04542	0.01464	-0.18324
C	-3.58064	3.83775	2.65259
H	-3.06175	1.30371	-0.27776
C	-4.18359	4.07775	1.40401
H	-7.82415	-3.83328	1.79393
C	-2.80153	2.68773	2.85168
C	-3.65820	-1.00768	5.36346
C	-2.63158	1.75197	1.80486
H	-4.45523	3.35838	-0.63193
C	0.57482	2.01290	1.46177
C	-3.99989	3.16648	0.35182
H	2.14034	3.42504	0.94677
H	-3.95377	0.49900	3.83214
H	3.37831	3.10891	3.11343
C	2.45332	2.54554	2.91595
C	0.75290	0.95408	3.64527
H	-4.78904	4.98413	1.25001
Cu	-1.51622	-1.23017	0.23696
C	-3.22708	2.00846	0.55214
C	0.05624	1.12078	2.43082
C	1.94579	1.66152	3.88161
H	-0.16597	-4.28126	1.01693
H	-3.71036	4.55752	3.47539
C	-1.61922	-2.31907	5.21286
H	2.47330	1.52751	4.83871
H	0.36068	0.28585	4.42553
H	0.02349	2.18407	0.52219
C	-3.28627	-0.28636	4.21527
P	-1.54336	0.26580	1.99604
C	1.76188	2.72096	1.70376
C	-2.82315	-2.01494	5.87290
H	-0.32195	-1.88568	3.52967
C	-2.07131	-0.57256	3.55586
C	-1.25299	-1.61633	4.05356
H	-4.60991	-0.77352	5.86460
H	-3.11238	-2.56778	6.77963
H	-0.96120	-3.11253	5.59989
C	-2.62227	-6.82357	0.58272
C	-2.90991	-5.59958	-0.04879
C	-2.92623	-4.40235	0.69710

H	-2.17518	-5.70012	3.79329
C	-6.64070	-0.67113	1.18370
C	-2.35582	-6.86606	1.96087
H	-2.13536	-7.82608	2.45229
C	-2.65377	-4.45071	2.08448
H	-3.12586	-5.58198	-1.12720
H	-2.61417	-7.75110	-0.01037
C	-2.37654	-5.67581	2.71121
H	-2.66181	-3.52456	2.68112
C	-5.06244	-3.37114	-3.76776
C	-4.88409	-3.09030	-2.40131
C	-3.59235	-3.11417	-1.83265
H	-1.79839	-3.95642	-4.64700
H	-4.65961	-0.19797	0.43380
C	-3.95903	-3.68746	-4.57713
H	-4.10340	-3.91374	-5.64470
C	-2.48404	-3.41500	-2.65916
H	-5.75783	-2.85520	-1.77548
H	-6.07533	-3.34858	-4.19861
C	-2.66854	-3.71181	-4.01856
H	-1.46808	-3.42291	-2.23347
C	-7.13189	-3.03307	1.48980
C	-5.85073	-3.36511	1.01984
C	-4.95060	-2.34819	0.62852
H	-6.94345	0.38528	1.24836
P	-3.27152	-2.73717	-0.04674
C	-7.53035	-1.68726	1.57095
H	-8.53542	-1.43087	1.93978
C	-5.35592	-0.99996	0.71989
H	-5.54975	-4.42188	0.95911
H	2.71461	0.59206	0.18531
H	4.17546	-0.05271	-0.34313

#Cu complex **6**

46

C	-2.24507	-14.19802	3.41014
C	-1.12472	-13.67490	4.10332
C	0.00311	-13.95168	3.32932
N	-0.46736	-14.59537	2.20072
N	-4.71834	-16.54687	-2.89768
H	-1.12810	-13.14398	5.06144
N	-1.83407	-14.76691	2.25923
H	2.64166	-13.31617	0.94437
C	0.15622	-15.26015	1.06317
C	1.32856	-17.49723	1.69938
H	1.32502	-19.66666	2.18643
C	0.78152	-18.76907	1.87201

C 1.35516 -14.51710 0.42605
N 1.64400 -13.36959 0.69394
N -6.19133 -18.11575 -1.70266
N 0.29054 -16.69691 1.27323
H -0.61498 -15.18393 0.26157
N -0.88784 -17.40122 1.20024
C -0.59849 -18.66968 1.55575
H -5.36707 -14.52929 -2.85440
C -5.44300 -18.15507 -0.45058
O 1.88435 -15.27023 -0.54314
C -3.74876 -16.35047 -1.82508
C -5.80262 -15.55015 -2.85521
C -5.31503 -17.89363 -2.86603
H -5.65984 -14.32985 -0.45791
H -4.69427 -18.97606 -0.48126
H -2.92259 -17.08838 -1.91650
H -3.29179 -15.34041 -1.90602
H -7.85950 -17.20086 -2.60006
C -6.01748 -15.38203 -0.43794
H -3.29158 -14.17592 3.74413
H -5.92095 -18.02544 -3.78625
H 1.07286 -13.04835 1.48861
H 1.04048 -13.69606 3.54240
P -4.52458 -16.52048 -0.08905
H -6.12854 -18.36877 0.39756
H 2.36956 -17.20644 1.83867
H -7.88621 -17.21570 -0.80397
H -6.41291 -15.66356 -3.77497
H -1.34627 -19.46855 1.58064
Cu -2.62049 -16.30692 1.18532
H -4.50824 -18.65571 -2.87186
N -6.69228 -15.70547 -1.69089
H -6.73076 -15.46681 0.41058
C -7.23470 -17.07579 -1.69149

#Cu complex **7**

68
N -0.97227 -0.95290 -2.45695
H -0.84497 1.25110 -4.93840
C -1.51772 0.07604 -3.13273
C 0.42583 -0.40703 -4.17359
C -0.67469 0.44826 -4.21273
H -4.56951 -0.09912 -0.22386
C -4.47911 -3.80794 0.71547
N 0.21808 -1.22687 -3.08509
H -0.77149 -6.32934 -4.54577
N -0.82362 -3.98217 -2.19854

N	0.25692	-3.64426	-2.98421
C	-1.33927	-5.10108	-2.73853
C	0.40527	-4.51323	-4.04815
C	-0.60082	-5.46536	-3.89417
C	2.34581	-1.62409	2.62289
H	2.50866	-4.19686	-3.06377
C	0.90868	-2.40744	-2.59098
C	2.44395	-2.40937	-2.66069
N	3.11913	-3.40215	-2.82573
O	2.91488	-1.18918	-2.33813
H	0.70534	-2.36775	-1.48972
H	2.92966	-1.43227	3.54643
H	0.04644	-3.63125	4.04566
H	1.21218	-2.52045	4.84476
C	0.63759	-2.70119	3.91269
H	1.09488	-0.14216	4.42777
H	3.07066	-1.72631	1.79080
H	-0.15987	0.51638	3.32275
C	0.93836	-3.32132	1.58317
P	-3.83046	-2.47400	-0.49357
H	1.54357	-0.56770	0.27196
N	-5.91065	-3.69335	0.97054
N	-6.54352	-2.73560	-1.20735
C	-4.66929	-1.00794	0.40669
H	0.18006	0.38142	0.93061
H	-5.01285	-3.65422	-2.34671
H	0.41706	-4.28789	1.75210
C	-6.87074	-1.47605	-0.51862
C	-6.25971	-2.40827	1.59927
H	-5.13506	-1.88318	-2.53713
N	-6.07613	-1.25455	0.70285
H	1.69642	-3.49707	0.78980
C	-1.21756	-1.80165	2.62252
H	-7.32731	-2.44765	1.90020
C	-6.70958	-3.84909	-0.25837
H	-7.77995	-3.90206	0.03041
H	-5.64889	-2.26620	2.51497
H	-4.11160	-0.81285	1.34776
C	-5.20767	-2.70885	-1.79718
H	-6.44004	-4.80359	-0.75640
H	-3.91532	-3.71748	1.66874
H	-6.72318	-0.62448	-1.21506
Cu	-1.59869	-2.38164	-0.97159
H	-7.94258	-1.50564	-0.23210
H	-4.24920	-4.81038	0.29613
H	1.29881	-0.44723	-4.84411
C	0.52012	-0.34330	3.49994
H	-1.91559	-0.94160	2.52683

H	-1.82958	-2.70480	2.83617
H	-2.44004	0.56921	-2.81944
N	1.47180	-0.45833	2.38472
H	1.21326	-4.43110	-4.78536
N	1.59279	-2.87838	2.80880
C	0.79657	-0.52860	1.09365
P	-0.34458	-2.05494	0.94271
H	-2.18471	-5.62266	-2.26753
H	3.78643	-3.23982	-3.59337
N	-0.28088	-1.56373	3.71390
